# Circulating Lipids, Statins, and Metformin in Thoracic and Abdominal Aortic Aneurysm: A Systematic Review and Meta-Analysis

**DOI:** 10.3390/life16101765

**Published:** 2026-10-21

**Authors:** Jose F. Rodríguez-Palomares, Eduard Ródenas-Alesina, Marco Tomasino, Yassin Belahnech, Arwa Mehmood, Toni Soriano-Colomé, Aleix Olivella, Gerard Oristrell, Sergio del Fresno, Leandro Santiago Videla, Ruperto Oliveró Soldevilla, Laura Galian-Gay, Ani Orchanian-Cheff, Farid Foroutan, Andrea Guala, Lydia Dux-Santoy, Gisela Teixidó-Tura, Ignacio Ferreira-González

**Affiliations:** 1 Department of Cardiology, Vall d’Hebron Hospital Universitari, Vall d’Hebron Barcelona Hospital Campus, Passeig de la Vall d’Hebron 119–129, 08035 Barcelona, Spain; eduard.rodenas@vallhebron.cat (E.R.-A.); marcotomasino21@gmail.com (M.T.); yassin.belahnech@vallhebron.cat (Y.B.); arwa.mehmood@vallhebron.cat (A.M.); toni.sorianocolome@vallhebron.cat (T.S.-C.); aleix.olivella@vallhebron.cat (A.O.); gerard.oristrell@vallhebron.cat (G.O.); sergiodfp12@gmail.com (S.d.F.); leandro_santiago_videla@hotmail.com (L.S.V.); rupertocarlos.olivero@vallhebron.cat (R.O.S.); laura.galian@vallhebron.cat (L.G.-G.); ignacio.ferreira@vallhebron.cat (I.F.-G.); 2 Cardiovascular Diseases, Vall d’Hebron Institut de Recerca (VHIR), Vall d’Hebron Barcelona Hospital Campus, Passeig de la Vall d’Hebron 119–129, 08035 Barcelona, Spain; andrea.guala@vhir.org (A.G.); lydia.duxsantoy@vhir.org (L.D.-S.); 3 Department of Medicine, Universitat Autònoma de Barcelona, 08193 Bellaterra, Spain; 4 Centro de Investigación Biomédica en Red de Enfermedades Cardiovasculares (CIBERCV), Instituto de Salud Carlos III, 28029 Madrid, Spain; 5 Library and Information Services, University Health Network, Toronto, ON M5T 2S8, Canada; ani.orchanian-cheff@uhn.ca; 6 Department of Health Research Methods, Evidence, and Impact, McMaster University, Hamilton, ON L8N 3Z5, Canada; farid.foroutan@uhn.ca; 7 Centro de Investigación Biomédica en Red de Epidemiología y Salud Pública (CIBERESP), Instituto de Salud Carlos III, 28029 Madrid, Spain

**Keywords:** abdominal aortic aneurysm, thoracic aortic aneurysm, aneurysm growth, dyslipidemia, statins, metformin, systematic review, meta-analysis

## Abstract

Thoracic and abdominal aortic aneurysms (TAA and AAA) progress silently, and no drug has been shown to slow their growth. Circulating lipids, statins, and metformin are candidate disease modifiers, but the evidence is heterogeneous and largely observational. We searched MEDLINE, Embase, and the Cochrane Library from inception to 24 March 2025, with an update of MEDLINE to 3 October 2026, for randomized and cohort studies of adults with established AAA or TAA. Aneurysm growth was standardized to mm/year and pooled with random-effects models when at least three comparable studies were available. Risk of bias and certainty of evidence were assessed with ROBINS-I and GRADE. Thirty-three studies were included, 32 observational and one randomized; fewer than 20% of participants were women. Circulating lipid fractions showed no consistent association with aneurysm growth. Statin use was associated with slower growth (mean difference −0.78 mm/year; 95% CI −1.13 to −0.43; *I*^2^ = 92.4%; 95% prediction interval −2.02 to 0.46 mm/year), lower all-cause mortality, and fewer aortic complications. In AAA, metformin use was associated with slower growth in observational studies (−0.34 mm/year; 95% CI −0.48 to −0.20; *I*^2^ = 46.3%), while the single randomized trial found no difference. Certainty of evidence was low to very low. In these largely observational data, aneurysm progression was more closely related to statin and metformin use than to circulating lipid levels, a pattern that is hypothesis-generating and from which causality cannot be inferred.

## 1. Introduction

Thoracic and abdominal aortic aneurysms (TAA and AAA, respectively) are common aortic diseases with a prolonged asymptomatic course but potentially catastrophic complications, including dissection and rupture [1,2,3]. Many aneurysms are incidentally detected and then monitored over time, creating a substantial clinical burden, with management largely guided by aneurysm size, growth rate, and risk of acute aortic events (AAE) [4,5,6,7,8]. In the general population, TAA prevalence is estimated at 0.1–0.3% [1], whereas AAA are more common (1–3% overall), rising to 4–8% in men aged 65 years and older [9,10]. In both conditions, elective repair is primarily guided by diameter thresholds (≈5.5–6.0 cm for TAA and ≈5.5 cm in men and ≈5.0 cm in women for AAA) [11,12].

Despite significant advances in surgical and endovascular techniques, no widely accepted pharmacologic therapy has been shown to reliably slow aneurysm growth or prevent AAE. Current medical management primarily targets global cardiovascular risk rather than the mechanisms of aneurysm disease per se [13,14]. This persistent gap has driven interest in therapies targeting inflammation, oxidative stress, extracellular matrix remodeling, and vascular smooth muscle cell phenotype, key processes in aneurysm pathogenesis and progression [13,15,16,17,18,19]. Recent syntheses have confirmed that this gap persists: no agent has shown a reproducible effect on aneurysm growth, and trials of antibiotics and of renin-angiotensin system blockade have been negative or inconclusive [20,21,22].

Dyslipidemia and lipid-lowering therapy could be relevant in aortic aneurysm disease because this condition, mainly AAA but also TAA, shares risk factors with atherosclerosis. In this sense, statins are widely prescribed for established indications in atherosclerosis, with a potential impact on aneurysm progression [13,14]. In addition to reducing low-density lipoprotein cholesterol (LDL-C), statins have anti-inflammatory and endothelial-stabilizing effects that could plausibly influence aneurysm progression [23,24]. Observational data suggest an association between statin use and slower AAA growth and fewer aneurysm-related events, but interpretation is limited by heterogeneity, confounding, and nonrandomized study designs [25]. A recent joint consensus document has set out the genetic, mechanistic and Mendelian randomization evidence implicating LDL cholesterol, triglyceride-rich lipoproteins and lipoprotein(a) in aneurysm biology, while noting that no randomized trial has yet shown that lipid lowering modifies aneurysm progression [26]. Evidence in TAA is more limited and inconsistent, with mixed findings after adjustment for indication and other confounders [27]. In parallel, cardiometabolic therapies, such as metformin, have been proposed as potential modifiers of aneurysm natural history. In this respect, epidemiologic studies have consistently shown smaller aortic diameters in patients with diabetes (DM) and slower aneurysm growth in those with established AAA, supporting a potential protective role of metabolic pathways and/or antidiabetic therapies [28]. However, the first randomized trial in a non-diabetic population failed to demonstrate a clear benefit [29]. In contrast, evidence linking metformin to TAA progression or AAE remains limited and largely observational [30].

Importantly, AAA and TAA represent biologically distinct entities that differ fundamentally in their pathophysiological substrates. AAA is closely linked to atherosclerotic risk factors, systemic inflammation, extracellular matrix degradation, and intraluminal thrombus biology, sharing genetic and environmental determinants with atherosclerosis. In contrast, TAA more frequently arises from medial degeneration, bicuspid aortic valve–associated structural changes, or genetic aortopathies, including Marfan syndrome and other heritable connective tissue disorders, in which atherosclerotic and inflammatory mechanisms play a comparatively limited role. This fundamental biological heterogeneity has direct implications for the interpretation of pooled analyses across aneurysm locations and for the expected effect size of lipid-modifying therapies. Accordingly, in this review, AAA and TAA are analyzed separately throughout.

This systematic review and meta-analysis aims to synthesize the available evidence on the associations between (i) changes in lipid fractions, (ii) statin use, and (iii) metformin use, and aortic aneurysm progression. By separately analyzing AAA and TAA, and integrating both aneurysm growth and clinical outcomes, we seek to clarify whether aneurysm progression is primarily driven by systemic lipid exposure or by downstream biological pathways potentially modifiable by pharmacological interventions.

## 2. Materials and Methods

### 2.1. Search Strategy and Study Selection

This systematic review and meta-analysis was conducted in accordance with the Preferred Reporting Items for Systematic Reviews and Meta-Analyses (PRISMA) 2020 statement. A comprehensive search strategy was developed using a combination of database-specific subject headings and text words for the main concepts of serum lipid levels or cholesterol-lowering agents, and aortic aneurysm or aortic aneurysm surgery. Searches were limited to studies in humans published in English, French, or Spanish. Conference proceedings were removed from Embase. We searched the following databases on 24 March 2025: Ovid MEDLINE; Ovid Embase; Cochrane Database of Systematic Reviews (Ovid); and Cochrane Central Register of Controlled Trials (Ovid). The search was updated in MEDLINE on 3 October 2026 using the same concepts and the same eligibility criteria, and the additional records were screened in duplicate. Grey literature sources (including preprints, dissertations, trial registries, and technical reports) were not systematically searched. Full search strategies are provided in File S1. The reference lists of included publications and of relevant systematic reviews and meta-analyses were also screened for eligible studies.

Eligible studies were required to report the progression of AAA or TAA in patients aged 18 years or older and to provide data on at least one of the following exposures: lipid fractions [total cholesterol, high-density lipoprotein cholesterol (HDL-C), low-density lipoprotein cholesterol (LDL-C), triglycerides, and lipoprotein(a)], statin therapy, or metformin use. Studies of TAA and AAA of any etiology were eligible. AAA cohorts predominantly comprised degenerative aneurysms, whereas TAA studies mainly included degenerative and bicuspid aortic valve–associated forms, with limited reporting of genetic etiologies. Outcomes of interest were clinical events, namely all-cause mortality, acute aortic syndromes, and need for aortic surgery, and/or aneurysm growth. Only randomized controlled trials and cohort studies were included. Studies involving postoperative patients or those assessing aneurysm incidence in individuals without aneurysmal disease at baseline were excluded.

Given the biological heterogeneity between AAA and TAA, all prespecified pooled analyses included subgroup analyses stratified by aneurysm location. Two studies included in the systematic review did not explicitly report aneurysm location and were therefore excluded from location-specific subgroup analyses; their retention in sensitivity analyses confirmed that excluding them did not materially alter overall pooled estimates. Studies that did not report growth as a continuous variable were included in the narrative synthesis of clinical outcomes but excluded from quantitative growth analyses.

Titles and abstracts were screened independently by two investigators, followed by full-text review of potentially eligible studies. Discrepancies were reviewed by a third investigator and resolved by consensus.

### 2.2. Data Extraction

Data were independently extracted by two reviewers using a standardized form, based on Research Electronic Data Capture (REDCap; Vanderbilt University, Nashville, TN, USA; https://www.project-redcap.org/). Extracted variables included study design, geographic location, sample size, aneurysm location, patient characteristics (age, sex, cardiovascular risk factors, comorbidities, and baseline aneurysm diameter), exposure definitions, outcome definitions, duration of follow-up, and effect estimates with corresponding measures of uncertainty. For clinical outcomes, fully adjusted effect estimates were preferentially extracted when available. For aneurysm growth, growth rates were extracted as reported and subsequently harmonized to a standard time scale to provide growth in mm per year.

### 2.3. Outcomes

The prespecified outcomes were: (1) clinical outcomes, including all-cause mortality, acute aortic syndromes, primarily aortic dissection and rupture, and need for aortic surgery; and (2) aneurysm growth, defined as the change in maximal aortic diameter over time, as assessed by ultrasound, echocardiography, or computed tomography. When necessary, aneurysm growth rates were standardized and expressed as millimeters per year (mm/year) to allow comparability across studies.

### 2.4. Risk of Bias Assessment

Risk of bias was assessed independently by two reviewers using the Cochrane RoB 2 tool for the randomized trial and ROBINS-I for the observational studies. Each observational study was appraised across the seven ROBINS-I domains: confounding, selection of participants into the study, classification of interventions, deviations from intended interventions, missing data, measurement of outcomes, and selection of the reported result. Because the exposures of interest were statin or metformin use as recorded in routine care, the domain of bias due to deviations from intended interventions was considered not applicable, and judgments are presented for the remaining six domains (Figure S1). Domain-level and overall judgments were assigned using the standard ROBINS-I categories of low, moderate, serious or critical risk of bias, or no information; no numerical quality score was derived. Disagreements were resolved by consensus.

### 2.5. Quantitative Synthesis

Meta-analyses were performed when at least three studies reported comparable effect measures for the same exposure–outcome association. For aneurysm growth, the effect measure was the mean difference (MD) between exposed and unexposed groups; for all-cause mortality, hazard ratios (HRs) were pooled on the logarithmic scale. Given the anticipated clinical and methodological heterogeneity, a random-effects model using restricted maximum likelihood (REML) estimation was applied. Statistical heterogeneity was assessed using the *I*^2^ statistic. For the primary growth analyses, we additionally report the between-study standard deviation (τ) and the 95% prediction interval, which describes the range within which the effect observed in a future comparable study would be expected to fall and which, unlike the confidence interval around the pooled estimate, does not narrow as studies accumulate.

Clinical outcomes were not meta-analyzed when studies differed substantially in outcome definitions, follow-up duration, or effect measures (e.g., odds ratios versus hazard ratios). In these cases, results were synthesized narratively.

### 2.6. Handling of Incomplete Variance Data

When studies reported mean differences with 95% confidence intervals, standard errors were derived using standard formulas [31]. For studies reporting pre- and post-intervention aneurysm diameters without direct measures of change variability, standard deviations of change were estimated assuming a within-group correlation coefficient of *r* = 0.7 between baseline and follow-up measurements [31]. When medians and interquartile ranges were reported, standard deviations were approximated using established methods [32,33].

### 2.7. Subgroup and Meta-Regression Analyses

Prespecified subgroup analyses were conducted according to aneurysm location (TAA versus AAA). Differences between subgroups were assessed using an interaction test. To explore potential sources of heterogeneity, random-effects meta-regression analyses were performed using study-level covariates, including mean age, sex distribution, history of coronary artery disease or myocardial infarction, and baseline aortic diameter, when available. Meta-regression analyses were considered exploratory.

### 2.8. Sensitivity Analyses

Sensitivity analyses for aneurysm growth included leave-one-out analyses to evaluate the influence of individual studies on pooled estimates. Additional analyses were conducted to assess the robustness of results to assumptions made during variance estimation.

### 2.9. Assessment of Small-Study Effects

Potential small-study effects and publication bias were assessed through visual inspection of funnel plots and formally evaluated using Egger’s test when at least ten studies were available. These analyses were considered exploratory.

### 2.10. Certainty of Evidence

The certainty of evidence for each outcome was assessed using the GRADE framework, considering study design, risk of bias, inconsistency, indirectness, imprecision, and potential publication bias (Table S1).

### 2.11. Protocol Registration and Statistical Software

This systematic review and meta-analysis was not prospectively registered in PROSPERO or in any other protocol registry. All analyses were performed in Stata version 19 (StataCorp LLC, College Station, TX, USA). The completed PRISMA 2020 checklist is provided in Table S2.

## 3. Results

### 3.1. Study Selection

**Figure 1:**
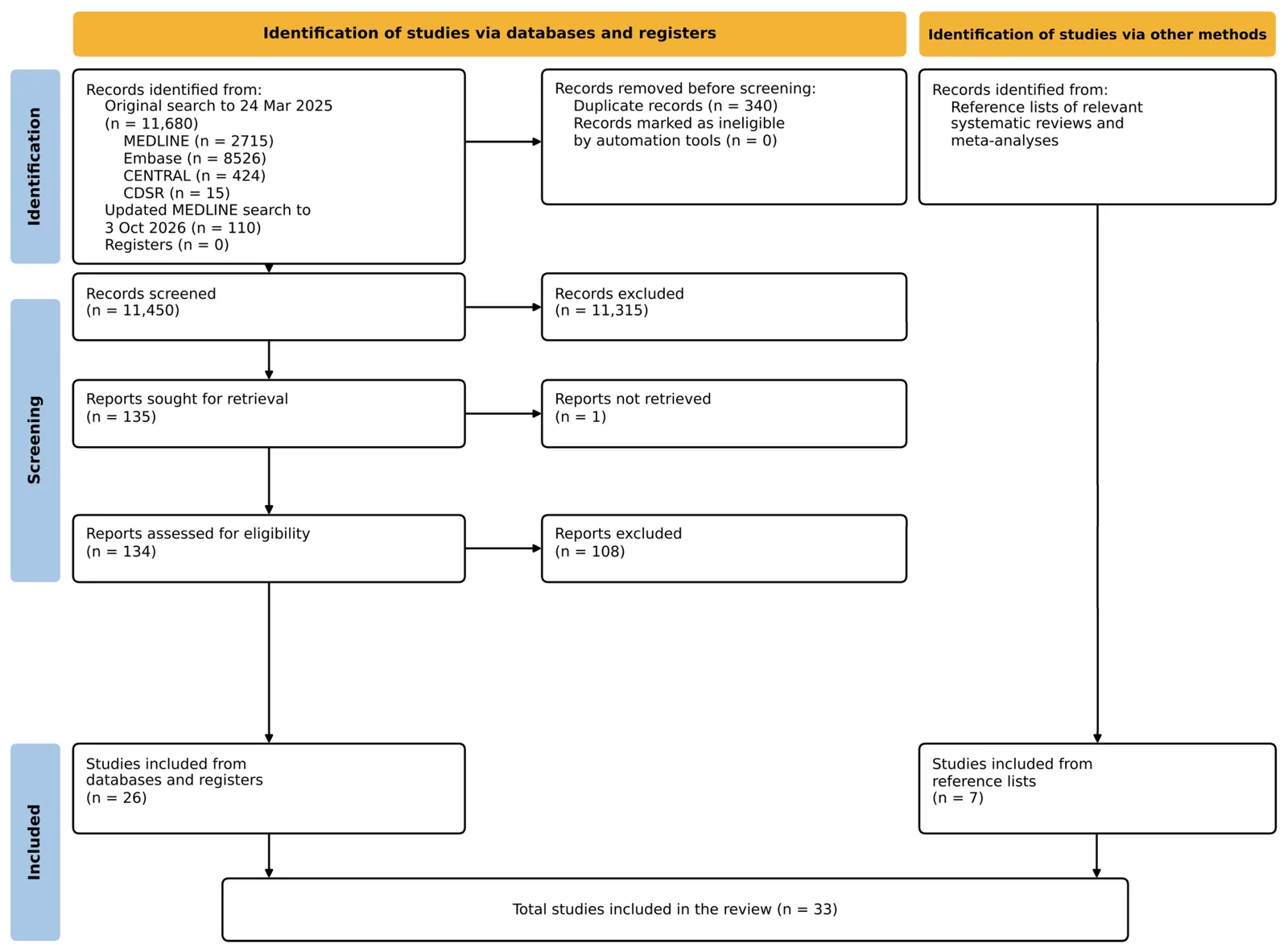
PRISMA 2020 flow diagram of study identification, screening, and inclusion. Counts include the updated MEDLINE search to 3 October 2026.

The original and updated searches yielded 11,790 records, of which 11,450 were screened after removal of duplicates. Of these, 135 reports were sought for retrieval and 134 were assessed in full text, since in one case the full text could not be retrieved. Twenty-six studies met the predefined inclusion criteria and were included in the systematic review. To further enhance the completeness of the search strategy, reference lists of relevant systematic reviews and meta-analyses were manually screened, yielding 7 additional eligible studies. Three of the included studies came from the updated search to 3 October 2026: a randomized placebo-controlled trial of metformin in AAA [29], a prospective cohort relating statin dose to AAA growth and clinical outcomes [34], and a propensity-matched cohort of metformin and AAA growth [35]. Thirty-three studies were therefore included in the present systematic review (Figure 1).

### 3.2. Characteristics of Included Studies

None of the 30 studies included from the original search were randomized clinical trials; all were observational, predominantly with retrospective cohort designs, and approximately half were conducted across multiple centers. Most studies (23, 77%) were published after 2010, although they frequently included older cohorts (median start of the inclusion period: 2002; interquartile range [IQR]: 1996–2005). Sixteen studies were conducted in Europe, eight in the United States, two in Asia, and four in other regions. Overall, the studies comprised 127,276 patients, with the majority contributed by a single large nationwide cohort [36], of which the 80,367 patients in the extracted comparison (metformin-treated patients with diabetes and patients without diabetes) were counted. Sample size was modest (<1000 patients) in 63% of studies. Regarding aneurysm location, 24 studies focused exclusively on AAA, four on TAA, and aneurysm location was not reported in 2 studies. The included article/cohort information is summarized in Table 1 and Table 2. The three studies added by the updated search comprised one randomized placebo-controlled trial [29] and two cohort studies [34,35].

### 3.3. Risk of Bias and Certainty of Evidence

The risk of bias assessment using the ROBINS-I tool is presented in Figure S1. Overall, most studies were judged to be at moderate to serious risk of bias, mainly driven by confounding and selection bias inherent to the observational design. According to the GRADE framework, the certainty of evidence for all outcomes was rated as low to very low (Table S1).

### 3.4. Lipid Fractions

#### 3.4.1. Lipid Fractions and Clinical Outcomes

No full-text article was found reporting the association between cholesterol fractions and clinical outcomes in patients with aortic aneurysms.

#### 3.4.2. Lipid Fractions and Aneurysm Growth

The association between lipid fractions and aneurysm growth was investigated in four studies. In a cohort of 451 patients with AAA, Ferguson et al. [49] reported no significant correlation between LDL-C levels and aneurysm growth rate, either among statin users (Pearson’s *r* = 0.072, *p* = 0.23) or non-users (*r* = −0.012, *p* = 0.87). Similarly, total cholesterol was not correlated with AAA growth in either group (*r* = 0.035, *p* = 0.56 in statin users; *r* = −0.026, *p* = 0.73 in non-users). Lindholt et al. [45] also found no significant correlations between AAA growth rate and LDL-C (*r* = 0.03, *p* = 0.97), HDL-C (*r* = 0.01, *p* = 0.91), total cholesterol (*r* = 0.17, *p* = 0.14), or lipoprotein(a) (*r* = 0.11, *p* = 0.31). Similarly, Schlösser et al. [47] reported no association between aneurysm growth and either LDL-C (*r* = 0.00, *p* = 0.98) or HDL-C (*r* = −0.01, *p* = 0.57). Consistently, Deeg et al. [59] did not demonstrate an association between aneurysm growth and conventional LDL-C or HDL-C levels; however, in the placebo arm of a doxycycline trial (*n* = 49), aneurysm expansion correlated with baseline total cholesterol and apolipoprotein B.

### 3.5. Statin Therapy

#### 3.5.1. Statins and Clinical Outcomes

Eight retrospective studies [27,37,38,39,40,41,42,43] evaluated the association between statins and aortic outcomes. Mean age ranged from 62 to 76 years, and women accounted for fewer than 20% of participants overall. Three studies [27,37,38] specifically reported outcomes in patients with TAA.

The association between statin use and aortic complications, including aortic dissection, aortic rupture, and the need for aortic surgical repair, was evaluated across 5 studies [27,37,38,41,42]. Owing to heterogeneity in follow-up duration and effect measures (odds ratios, hazard ratios, or unadjusted significance only), a quantitative meta-analysis of aortic events was not feasible. However, all five studies reported associations in the direction of a lower risk of aortic dissection, rupture, or need for surgical repair with statin therapy, although in one [27] the association did not reach statistical significance. Angeloni et al. [27] reported a nonsignificant trend toward fewer aortic events in patients with ascending aortic aneurysms (HR 0.69, 95% CI 0.47–1.01). Significant reductions were observed by Jovin et al. [37] in TAA (adjusted HR 0.58, 95% CI 0.42–0.80, mainly through fewer aneurysm repairs) and in large European [42] and US cohorts [38], with odds ratios ranging from 0.43 to 0.50. In addition, Mosorin et al. [41] demonstrated improved survival free from aneurysm repair or rupture among statin users.

**Figure 2:**
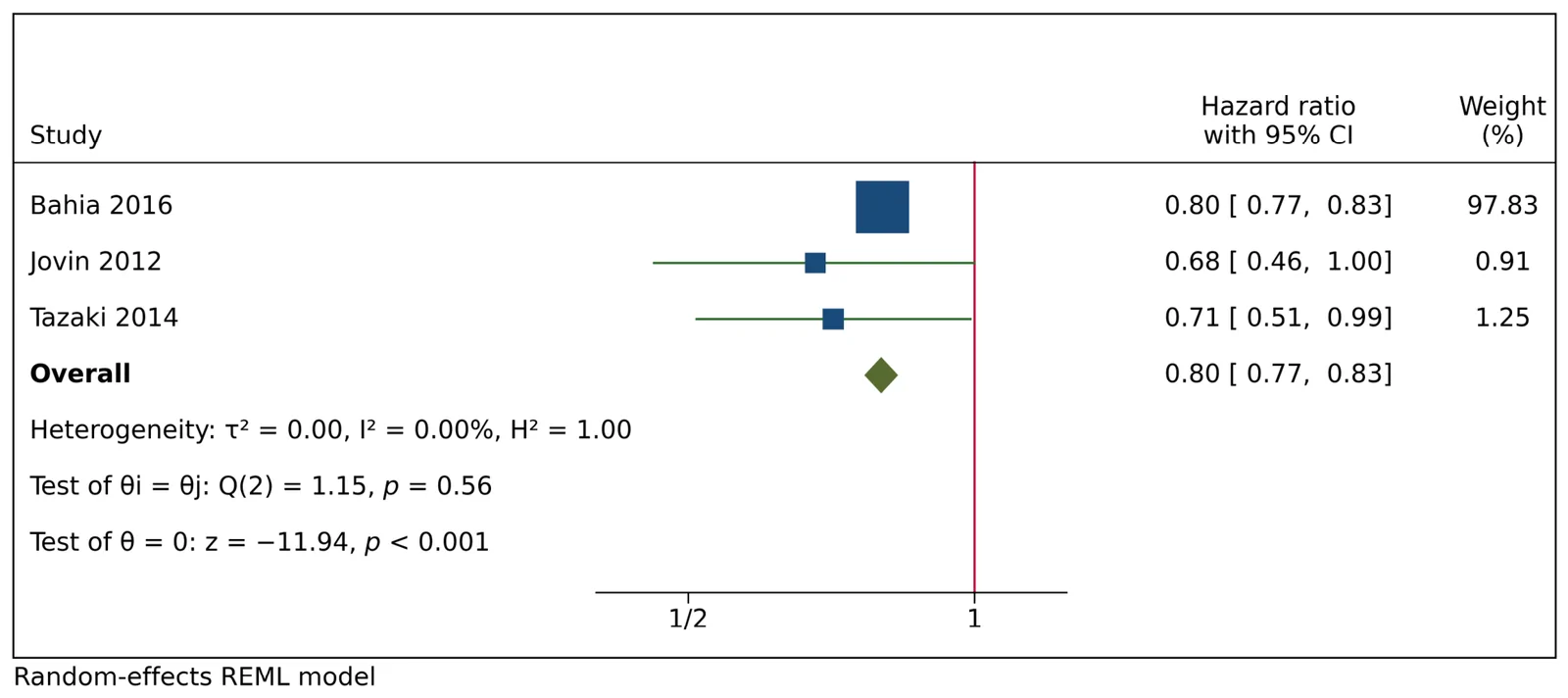
Forest plot of pooled hazard ratios for the association between statin use and all-cause mortality. Two of the three studies reported death from any cause; the third reported a composite of all-cause death, myocardial infarction and stroke, and the sensitivity analysis excluding it is reported in the text. Squares represent study-specific estimates, with size proportional to study weight, and horizontal lines their 95% CIs; the diamond represents the pooled estimate, and the vertical line indicates no effect. Studies are labeled by first author and year of publication: Bahia 2016 [43], Jovin 2012 [37], and Tazaki 2014 [39]. Random-effects model with restricted maximum likelihood (REML) estimation. CI, confidence interval.

Four retrospective studies [37,39,40,43] evaluated the association between statin therapy and all-cause mortality, three of which [37,39,43] reported hazard ratios: one AAA cohort [43], one TAA cohort [37], and one registry of patients with coronary artery disease and aortic aneurysm or dissection [39]. These three studies were meta-analyzed, showing a significant association between statin use and reduced all-cause mortality (pooled HR 0.80, 95% CI 0.77–0.83, *I*^2^ = 0.0%; Figure 2). This association was driven almost entirely (97.8% of the weight) by a UK primary care cohort of 12,485 patients with AAA [43], in which all-cause death was ascertained from general practice records and modeled with time-dependent covariates. Outcome definitions were not identical across these three studies. The AAA cohort [43] and the TAA cohort [37] both reported death from any cause, the latter as a crude hazard ratio over a median of 3.6 years. The third study [39] was a registry of patients undergoing coronary revascularization, in which 31% of those with aortic aneurysm or dissection had already undergone aneurysm repair, and its reported endpoint was a composite of all-cause death, myocardial infarction and stroke (adjusted HR 0.71, 95% CI 0.51–0.99) rather than mortality alone. In a sensitivity analysis excluding that registry, the pooled estimate was unchanged (HR 0.80, 95% CI 0.77–0.83, *I*^2^ = 0.0%), with the UK cohort then contributing 99.1% of the weight. In the three-study analysis, replacing the crude HR of the TAA cohort [37] with its adjusted HR (1.03, 95% CI 0.62–1.72) also left the pooled estimate unchanged (HR 0.80, 95% CI 0.77–0.83, *I*^2^ = 0.0%). Because only two studies remain once the registry is removed, the estimate without the registry is reported for completeness rather than as an independent pooled result, and the mortality analysis should be read throughout as resting on one large cohort with two smaller studies pointing in the same direction. The fourth study, by Sukhija et al. [40], reported a protective association for all-cause mortality among patients with AAA (OR 0.29, *p* = 0.049). For aortic events (dissection, rupture, need for surgical repair), heterogeneity in outcome definitions across studies, including differences in whether elective and emergent repair were combined, and whether rupture and dissection were pooled or separated, precluded formal meta-analysis; results for this endpoint are therefore based on narrative synthesis of five observational studies, all reporting a consistent protective direction for statin use (detailed outcome definitions per study are provided in Table S1).

Overall, although the certainty of evidence was low, the consistent direction of associations suggests a beneficial effect of statin therapy on aortic events, particularly in patients with TAA, and a potential reduction in all-cause mortality, although the pooled estimate relies almost exclusively on a single AAA cohort (Table 1).

#### 3.5.2. Statins and Aneurysm Growth

**Figure 3:**
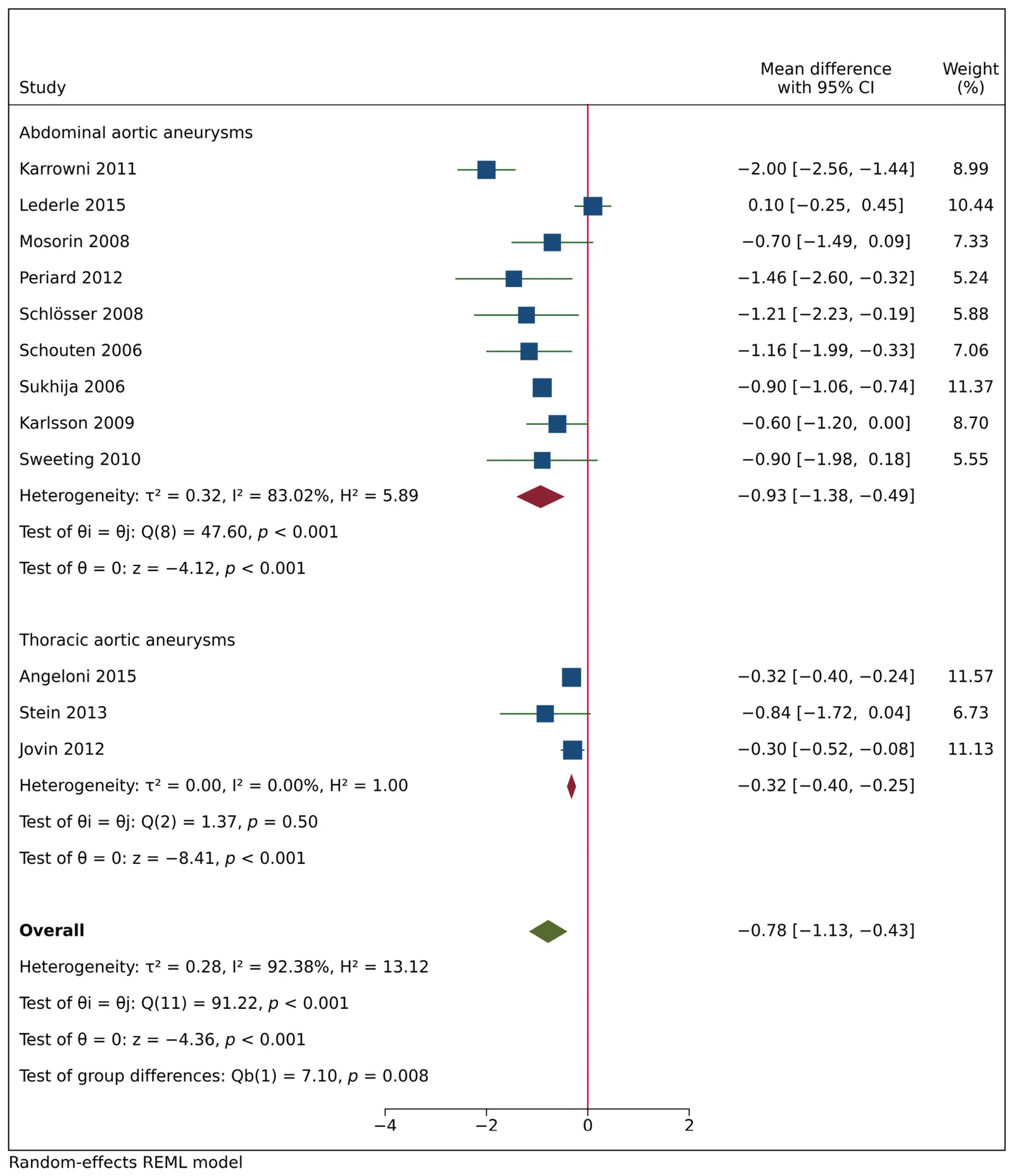
Forest plot of mean differences in annual aneurysm growth (mm/year) associated with statin use, stratified by aneurysm location. Negative values favor statin use. Squares represent study-specific estimates, with size proportional to study weight, and horizontal lines their 95% CIs; red diamonds represent subgroup pooled estimates, the green diamond the overall pooled estimate, and the vertical line no effect. Studies are labeled by first author and year of publication: Karrowni 2011 [51], Lederle 2015 [53], Mosorin 2008 [41], Periard 2012 [52], Schlösser 2008 [47], Schouten 2006 [46], Sukhija 2006 [40], Karlsson 2009 [48], Sweeting 2010 [50], Angeloni 2015 [27], Stein 2013 [38], and Jovin 2012 [37]. Random-effects REML model. CI, confidence interval.

Twelve studies evaluating the association between statin therapy and aneurysm growth were included in the quantitative synthesis (Table 2) [27,37,38,40,41,46,47,48,50,51,52,53]. Statin use was associated with a significant reduction in aneurysm growth, with a pooled mean difference (MD) of −0.78 mm/year (95% CI −1.13 to −0.43, *p* < 0.001; Figure 3). However, substantial heterogeneity was observed (*I*^2^ = 92.4%), indicating marked variability in effect estimates across studies. Potential sources of this heterogeneity include differences in imaging modality (ultrasound versus computed tomography versus echocardiography), measurement intervals, intra- and inter-reader variability, case-mix, and varying proportions of concomitant cardiovascular therapies across cohorts. To express this variability on the scale of the outcome, the between-study standard deviation was 0.53 mm/year and the 95% prediction interval ran from −2.02 to 0.46 mm/year, that is, it included the null, so a future comparable study could plausibly observe no slowing of growth at all. Heterogeneity was also unevenly distributed across aneurysm location: it was high in the abdominal subgroup (nine studies, *I*^2^ = 83.0%, pooled MD −0.93 mm/year, 95% CI −1.38 to −0.49) and absent in the thoracic subgroup (three studies, *I*^2^ = 0.0%, pooled MD −0.32 mm/year, 95% CI −0.40 to −0.25), and aneurysm location was the only covariate significantly associated with the effect estimate in meta-regression. Design, publication era, the definition of statin exposure (ever use, use at baseline, or dose) and the duration of follow-up also differed systematically between these two groups of studies, so aneurysm location and study methodology are confounded with one another and cannot be separated at the study level. Given the very high level of statistical heterogeneity (*I*^2^ = 92.4%), the pooled estimate should not be interpreted as a precise measure of the treatment effect, but rather as an exploratory summary of the direction of effect across studies. The consistency in the direction of association across heterogeneous cohorts, despite variability in magnitude, suggests a potential signal; however, the quantitative estimate itself is highly unstable and largely driven by differences in study design, imaging methodology, and population characteristics.

In subgroup analyses, studies including patients with AAA showed a pooled MD of −0.93 mm/year (95% CI −1.38 to −0.49, *p* < 0.001; *I*^2^ = 83.0%), whereas studies including patients with TAA showed a smaller reduction in growth (MD −0.32 mm/year, 95% CI −0.40 to −0.25, *p* < 0.001; *I*^2^ = 0.0%). The test for subgroup differences was significant (*p* = 0.008), indicating a larger association in AAA than in TAA. In leave-one-out analyses, the pooled estimate ranged from −0.61 to −0.87 mm/year, and all confidence intervals excluded the null. The updated search identified a prospective cohort of 998 men with screening-detected AAA in which statin exposure was quantified as defined daily doses [34]. Because the effect measure was a dose–response coefficient rather than a comparison of users with non-users, it could not be pooled with the studies above. In that cohort, each additional defined daily dose per day was associated with a change in growth rate of −0.22 mm/year (95% CI −0.39 to −0.06), and each doubling of statin dose with a lower risk of repair (HR 0.82, 95% CI 0.70–0.97) and of the composite of repair, rupture and death (HR 0.83, 95% CI 0.73–0.94). Exploratory meta-regression did not identify mean age (β = −0.019 mm/year per year of age, *p* = 0.83), the proportion of male participants (β = −0.016, *p* = 0.31), or baseline aortic diameter (β = 0.039 mm/year per mm, *p* = 0.34) as significant modifiers of the association between statin use and aneurysm growth; one study did not report these characteristics and was excluded from this analysis [53]. Aneurysm location was the only covariate significantly associated with the effect estimate (β = 0.71, *p* = 0.008).

Visual inspection of the funnel plot suggested asymmetry, particularly among smaller studies, raising the possibility of small-study effects or publication bias (Figure S2). However, Egger’s test did not provide statistical evidence of small-study effects (*p* = 0.13). Given the limited number of studies and substantial heterogeneity, these findings should be interpreted with caution.

### 3.6. Metformin

#### 3.6.1. Metformin and Clinical Outcomes

Two studies evaluated the association between metformin use and clinical outcomes [36,44]. In a nationwide cohort of US veterans with AAA, Sutton et al. [36] reported lower all-cause mortality among metformin-treated patients with diabetes than among patients without diabetes during the first 10 years after diagnosis (adjusted HR 0.88, 95% CI 0.86–0.90). Among patients with AAA, Golledge et al. [44] observed that metformin was associated with a reduction in a composite endpoint of aortic rupture or the need for endovascular repair or open surgery (HR 0.63, 95% CI 0.44–0.93, *p* = 0.018) (Table 1).

Importantly, outcome definitions varied substantially across studies. While some cohorts defined aortic events as a composite of rupture, dissection, and surgical repair, others reported these endpoints separately or included elective procedures. This heterogeneity limits comparability and reinforces that conclusions regarding clinical outcomes are based on directionally consistent but non-uniform observational evidence.

#### 3.6.2. Metformin and Aneurysm Growth

Six observational studies, comprising eight distinct cohorts of patients with abdominal aortic aneurysm (AAA), examined the association between metformin use and aneurysm growth [44,54,55,56,57,58]. When studies compared aneurysm growth among metformin-treated diabetic patients with both diabetic patients not receiving metformin and non-diabetic patients, only the comparison between metformin-treated diabetics and diabetic non-users was included in the analysis to minimize confounding (Table 2).

**Figure 4:**
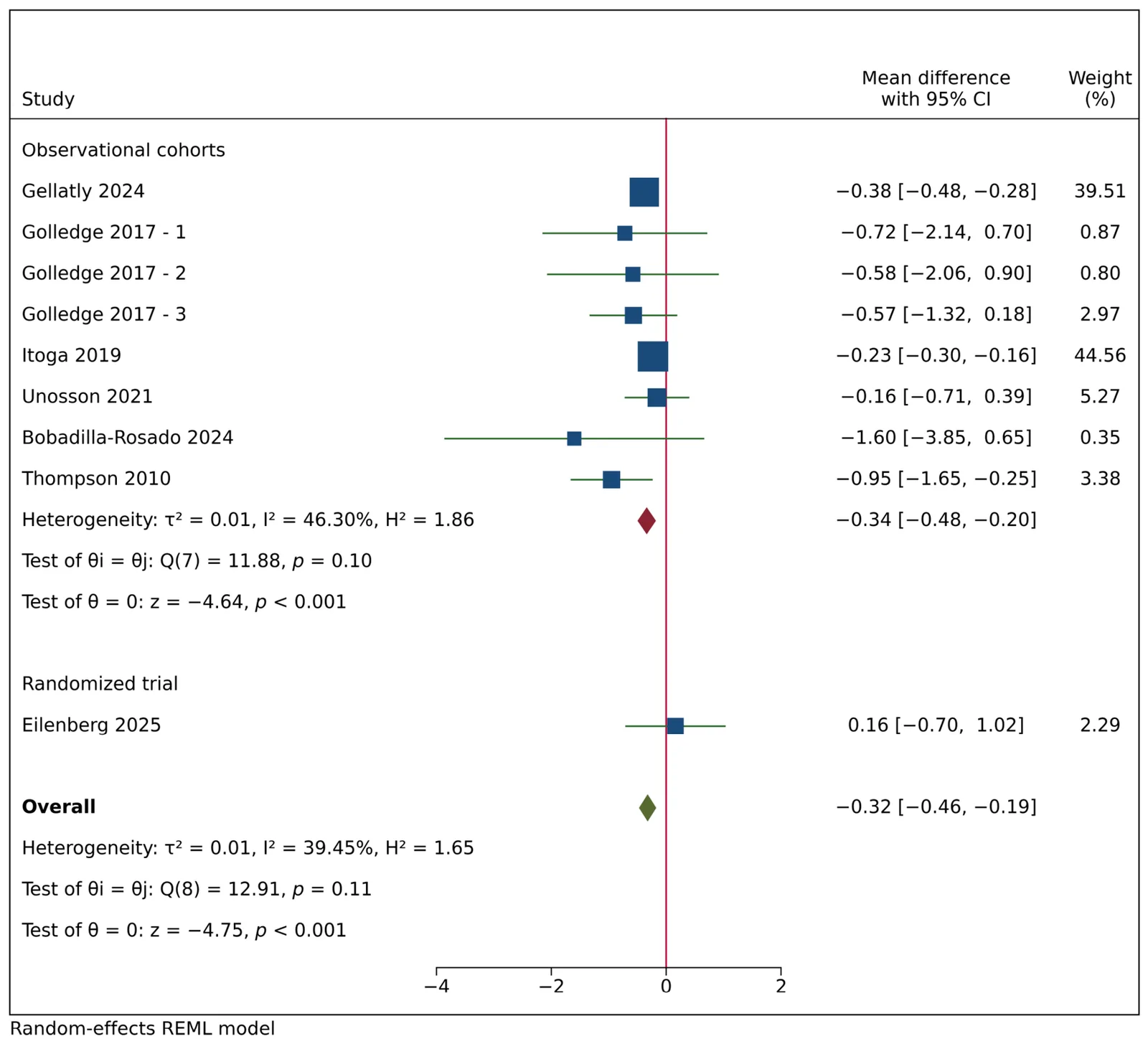
Forest plot of pooled mean differences in annual aneurysm growth (mm/year) associated with metformin use in abdominal aortic aneurysm. Negative values favor metformin use. The upper diamond pools the observational cohorts and is the primary estimate; the overall diamond additionally includes the randomized trial. Squares represent study-specific estimates, with size proportional to study weight, and horizontal lines their 95% CIs; the red diamond represents the pooled estimate of the observational cohorts, the green diamond the overall pooled estimate, and the vertical line no effect. Studies are labeled by first author and year of publication: Gellatly 2024 [57], Golledge 2017 [44] (cohorts 1 to 3 are the three cohorts reported in that study), Itoga 2019 [55], Unosson 2021 [56], Bobadilla-Rosado 2024 [58], Thompson 2010 [54], and Eilenberg 2025 [29]. Random-effects REML model. CI, confidence interval.

Most studies reported mean differences between groups with associated *p*-values or 95% CI or provided pre- and post-treatment aneurysm diameters in both groups, allowing calculation of standard errors. In the pooled analysis (Figure 4), metformin use was associated with a reduced annual AAA growth rate, with a pooled MD of −0.34 mm/year (95% CI −0.48 to −0.20), with moderate heterogeneity (*I*^2^ = 46.3%; τ = 0.10 mm/year; 95% prediction interval −0.65 to −0.03 mm/year). The updated search identified the first randomized evidence in this field: in 58 non-diabetic patients with small AAA, metformin did not significantly change the maximal aortic diameter at 12 months compared with placebo (between-group difference 0.16 mm, 95% CI −0.70 to 1.02), although the trial did not reach its recruitment target [29]. Adding this trial to the pooled analysis did not materially alter the estimate (MD −0.32 mm/year, 95% CI −0.46 to −0.19; *I*^2^ = 39.4%). A further propensity-matched cohort of 986 patients under AAA surveillance reported lower growth among metformin users (1.40 mm/year, 95% CI 1.05–1.88) than among patients taking neither metformin nor renin-angiotensin system blockade (1.94 mm/year, 95% CI 1.78–2.12) [35].

Meta-regression analysis showed that age did not modify the association between metformin use and aneurysm growth. In contrast, baseline aortic diameter was significantly associated with the observed effect, suggesting a greater benefit of metformin at smaller aneurysm diameters (β-coefficient 0.12, *p* = 0.023).

Visual inspection of the funnel plot suggested possible publication bias, as small studies with neutral or negative results appeared underrepresented (Figure S3). However, this asymmetry was not confirmed by Egger’s test (*p* = 0.12), which was considered exploratory because fewer than ten cohorts were available. The pooled reduction of 0.34 mm/year, while statistically significant, is modest and approaches the lower limit of ultrasound measurement precision in routine clinical practice. Its clinical relevance should therefore be interpreted cautiously, particularly given that the pooled estimate rests on observational cohorts, and that significant residual confounding is expected: patients receiving metformin differ systematically from untreated controls in terms of diabetes severity, glycemic control (HbA1c data were not reported in any included study), renal function (which may preclude metformin use in more severe disease), and concomitant antidiabetic and cardiovascular therapies. These confounders could plausibly inflate or attenuate the observed association. Overall, despite low certainty in the evidence, the consistent direction of findings across cohorts supports a potential association between metformin use and reduced aneurysm growth, warranting further prospective investigation.

## 4. Discussion

This systematic review and meta-analysis describes a discordance within the available observational evidence: circulating lipid fractions were not reliably associated with aneurysm growth, whereas statin and metformin use were consistently associated with slower progression and better clinical outcomes. This is a description of what the included studies report rather than a demonstrated mechanism, since lipid levels and drug exposure were examined with different designs and carry different susceptibility to confounding. This finding suggests that aneurysm progression may be driven primarily by local vascular processes, including inflammation, extracellular matrix remodeling, and metabolic signaling, rather than by systemic lipid levels alone.

Although there is biological plausibility linking dyslipidemia to aneurysm development (particularly for AAA, which shares multiple risk factors with atherosclerosis) [60,61,62], this review did not identify convincing evidence that circulating cholesterol fractions are independently associated with aneurysm growth. Across different observational cohorts, LDL-C, HDL-C, and total cholesterol showed no consistent association with aneurysm expansion rates [45,47,49]. Only one small study, restricted to the placebo arm of a doxycycline trial, found associations of baseline total cholesterol and apolipoprotein B with aneurysm growth [59]. Importantly, the apparent lack of association between circulating lipid levels and aneurysm growth should not be interpreted as evidence of the absence of a biological relationship. Rather, it likely reflects methodological limitations inherent to the available studies, including reliance on single baseline lipid measurements, regression dilution bias, lack of information on lipid-lowering therapy intensity and LDL-C target attainment, and limited statistical power. These factors may substantially attenuate true associations and contribute to the observed discrepancy between lipid levels and treatment effects.

This interpretation is consistent with the role of local vascular processes rather than systemic lipid levels. In contrast, the association between lipid-lowering and metformin therapies and aneurysm growth suggests that aneurysm progression could be primarily governed by local aortic wall inflammation, extracellular matrix degradation, intraluminal thrombus activity, and biomechanical stress, processes that are only weakly reflected in circulating lipid concentrations [16,63,64]. In any case, the limited number of studies, modest sample sizes, and the reliance on simple correlation analyses restrict the strength of these conclusions. Importantly, no studies linked cholesterol fractions directly to hard clinical outcomes, highlighting a substantial evidence gap. Taken together, these considerations suggest that the current evidence base is insufficient to exclude a biologically meaningful role of lipid fractions in aneurysm progression, and that the apparent lack of association should be interpreted with caution.

Statin therapy is consistently associated with more favorable clinical outcomes in observational studies, with reductions in adverse aortic events and need for surgical repair, and lower all-cause mortality, although the adjusted mortality association in TAA was null [37]. These benefits are unlikely to be mediated solely by LDL-C reduction and appear to arise primarily from pleiotropic effects on vascular biology, including anti-fibrotic actions on vascular smooth muscle cells [65], inhibition of vascular inflammation [66,67], and mitigation of oxidative stress via inhibition of the mevalonate pathway [68]. The pooled reduction of approximately 0.8 mm/year in aneurysm growth (primarily in AAA) is modest but potentially clinically meaningful in a slowly progressive disease, as it delays surgical intervention thresholds [25,34]. The magnitude of benefit was greater in AAA than in TAA (test for subgroup differences, *p* = 0.008), reflecting the more substantial contribution of modifiable inflammatory and atherosclerotic processes in abdominal disease. In contrast, TAA more frequently results from genetic or structural etiologies, in which these mechanisms play a limited role [65,69], and current evidence does not support routine statin use to slow aneurysm progression in genetic aortopathies such as bicuspid aortic valve–associated disease [70,71], or Marfan syndrome, where experimental data have not translated into human benefit [72,73]. An important caveat in interpreting the statin-outcomes associations is the potential for healthy user and treatment intensity bias. Patients prescribed statins are more likely to be receiving aggressive global cardiovascular risk management, and may benefit from more frequent clinical surveillance and lower thresholds for imaging or aortic repair. Without individual-level data on concomitant therapies and the intensity of cardiovascular risk management, it is not possible to attribute the observed outcome benefits specifically to statin therapy. This confounding by indication cannot be resolved by the available observational data and represents a fundamental limitation of the current evidence base, which can only be overcome by adequately powered randomized trials.

The distinction between a statistical association and a clinical effect deserves to be made explicit. A difference of 0.78 mm/year, if real and sustained, would amount to roughly 4 mm over five years of surveillance, which is of the same order as the gap between successive diameter thresholds and could therefore postpone the point at which repair is considered. Three caveats make that extrapolation fragile. First, the estimate comes from between-group comparisons in observational cohorts rather than from within-patient change after starting treatment, so it describes how statin users differed from non-users and not what happens when a statin is prescribed. Second, its prediction interval includes no difference at all. Third, a difference of this size is close to the measurement error of ultrasound, for which intra- and inter-observer repeatability coefficients of about 2 to 4 mm are commonly reported [74,75], so it is not detectable in an individual patient and becomes visible only across cohorts. The practical consequence is that these data do not support prescribing a statin for the purpose of slowing aneurysm growth, and do not justify lengthening surveillance intervals in patients already taking one. What they do reinforce is the existing recommendation to treat the atherosclerotic risk that most of these patients carry, an indication that stands independently of aneurysm behavior.

Metformin emerged as a promising candidate therapy, particularly for AAA [14]. Across multiple observational cohorts, metformin use was associated with reduced aneurysm growth and improved clinical outcomes, including lower mortality and fewer aneurysm-related events. However, in contrast to statins, which act on key structural and inflammatory drivers of aortic wall degeneration, metformin appears to have a more context-dependent effect, potentially explaining the smaller magnitude of benefit observed. The pooled reduction in growth rate, though smaller than that observed for statins, was consistent and accompanied by only moderate heterogeneity. A contemporary synthesis of glucose-lowering therapy in AAA reached the same conclusion, that the epidemiological signal is consistent and biologically plausible but that randomized confirmation is still lacking, and found no human evidence for newer agents such as SGLT2 inhibitors or GLP-1 receptor agonists [76].

The inverse association between diabetes and AAA progression has long been observed, and metformin may represent a key mediator of this phenomenon. Proposed mechanisms include modulation of inflammatory signaling, reduced matrix metalloproteinase activity, and favorable effects on vascular metabolism [77,78]. The observation that metformin’s effect was greater in patients with smaller baseline aneurysm diameters suggests a potential role in early disease modification.

Although observational studies suggest that metformin is associated with slower aneurysm growth and fewer aneurysm-related events, the available evidence remains nonrandomized and limited primarily to patients with AAA, predominantly those with diabetes. Whether these effects extend to non-diabetic patients or to patients with TAA remains uncertain, and the only randomized trial in non-diabetic patients has not demonstrated a clear benefit, highlighting the challenges of translating observational associations into clinical efficacy. In this context, the MetAAA trial, which randomized non-diabetic patients with AAA but did not reach its recruitment target, found no significant difference in aneurysm growth at 12 or 18 months [29,79]. Larger ongoing randomized trials, including MAT [80], MAAAGI [81], and LIMIT [82], are designed to provide more definitive evidence on the effects of metformin on aneurysm growth and clinical outcomes. These results will be critical in determining whether metformin has a proper disease-modifying role in AAA or whether prior findings primarily reflect residual confounding rather than a direct therapeutic effect.

### Limitations

Several limitations must be acknowledged. Randomized evidence remains almost absent, which is the central constraint of this analysis; 32 of the 33 included studies were observational, predominantly retrospective cohort designs, and thus prone to confounding, selection bias, and indication bias (causal inference is not possible from this evidence base). The updated search allowed the only randomized trial of metformin in AAA (MetAAA) to be included [29]; it did not reach its recruitment target, carries little weight in the pooled analysis, and does not by itself resolve the question. In addition, one study contributing to the pooled mortality analysis enrolled patients with coronary artery disease and aortic aneurysm or dissection, some of whom had undergone prior aortic repair [39]. The high statistical heterogeneity in the statin–growth meta-analysis (*I*^2^ = 92.4%) substantially limits the interpretability of the pooled estimate, which should be considered exploratory; sources include differences in imaging modality, measurement protocol, follow-up interval, and patient case-mix that could not be fully explained by meta-regression. For the metformin analysis, confounding by diabetes severity, renal function, HbA1c values (not reported in any included study), and concomitant antidiabetic and cardiovascular therapies cannot be excluded. A further and arguably more important source of residual confounding is blood pressure. Arterial hypertension and its treatment are among the best-established determinants of aneurysm growth and of the risk of dissection and rupture [20], yet blood pressure values, the proportion of patients with controlled hypertension and the use of antihypertensive drugs were reported inconsistently across the included studies and were rarely balanced between exposed and unexposed groups. In the largest cohort contributing to the mortality analysis, antihypertensive treatment was itself associated with better survival to a degree comparable with statins [43], which illustrates how closely these exposures travel together in routine care. The associations reported here therefore cannot be separated from differences in blood pressure control between the groups being compared, and none of the included studies was designed to do so. Also, two studies did not report aneurysm location and were excluded from location-specific subgroup analyses; sensitivity analyses confirmed this did not materially alter pooled estimates. TAA was markedly underrepresented (4 studies), preventing firm conclusions for this entity, and the apparent benefit of statins in TAA should be considered preliminary. Additionally, women comprised fewer than 20% of participants overall, precluding sex-specific analyses and limiting generalizability to women (a relevant gap given known sex differences in aneurysm biology and outcomes), acknowledged in line with SAGER guidelines. Finally, grey literature was not systematically searched, and the review was restricted to English, French, and Spanish publications, introducing potential publication bias that could not be fully excluded despite nonsignificant Egger’s test results.

## 5. Conclusions

Circulating lipid fractions were not consistently associated with aneurysm progression, likely reflecting methodological limitations and the inability of systemic lipid measurements to capture local aortic wall biology. In contrast, statin and metformin use were consistently associated with slower aneurysm growth and improved clinical outcomes, particularly in AAA; however, these findings are derived from heterogeneous observational data, the prediction interval around the statin estimate includes no effect, and differences in blood pressure control between the groups compared could not be accounted for, so they should be considered hypothesis-generating. Adequately powered randomized trials with phenotype-specific stratification are required to establish causality and define the clinical role of these therapies.

## Figures and Tables

**Table 1:** Studies evaluating the association of statins and metformin with clinical outcomes in thoracic and abdominal aortic aneurysms.

**Thoracic aorta (TAA)**								
**Study (year)**		**Design**	**Aortic segment**	***N***	**Treatment exposure**	**Follow-up**	**Outcome(s) assessed**	**Main outcome findings**
Jovin et al. (2012) [37]		Retrospective cohort with multivariable and propensity-score adjustment	TAA (ascending, arch, descending, thoracoabdominal)	649	Statin therapy vs. no statin	Median 3.6 years (up to 10 years)	All-cause mortality; composite of death, rupture, dissection, or repair	Statins were associated with a lower risk of the composite outcome (adjusted HR 0.58, 95% CI 0.42–0.80), mainly through fewer aneurysm repairs; no independent association with all-cause mortality (adjusted HR 1.03, 95% CI 0.62–1.72).
Stein et al. (2013) [38]		Retrospective cohort (single-center database)	Thoracic aorta (root, ascending/arch, descending/thoracoabdominal)	1560	Statin therapy vs. no statin	Up to 10 years	Death, dissection, rupture; need for surgical repair	Statins were associated with fewer adverse aortic events and a reduced need for surgical repair.
Tazaki et al. (2014) [39]		Multicenter observational registry (CREDO-Kyoto PCI/CABG cohort)	Aortic aneurysm or dissection (predominantly thoracic) in patients with CAD	699	Statin therapy at discharge vs. no statin	Median ~5 years	Composite of all-cause death, myocardial infarction, or stroke	Statins were associated with a lower risk of major cardiovascular events (adjusted HR 0.71, 95% CI 0.51–0.99)
Angeloni et al. (2015) [27]		Retrospective cohort with propensity-score matching	Ascending thoracic aortic aneurysm	658	Statin therapy vs. no statin	3 years	Composite of death, aortic dissection/rupture, or surgical repair	Statins were associated with improved freedom from the composite outcome at 3 years (HR 0.69, 95% CI 0.47–1.01; borderline statistical significance)
**Abdominal aorta (AAA)**								
	**Study (year)**	**Design**	**Aortic segment**	***N***	**Treatment exposure**	**Follow-up**	**Outcome(s) assessed**	**Main outcome findings**
**Statins**	Sukhija et al. (2006) [40]	Retrospective observational cohort	Infrarenal abdominal aortic aneurysm (non-operated)	130	Statin therapy vs. no statin	~45 months	All-cause mortality	Mortality was lower among statin-treated patients compared with non-users (5% vs. 16%)
	Mosorin et al. (2008) [41]	Retrospective observational cohort	Small infrarenal AAA	121	Statin therapy vs. no statin	Mean 3.6 years	Freedom from aneurysm repair or rupture	Statins were associated with a significantly lower risk of aneurysm repair or rupture.
	Gokani et al. (2015) [42]	Retrospective analysis of prospectively collected data	Large infrarenal AAA	983	Statin use vs. no statin	Cross-sectional at presentation	AAA rupture (ruptured vs. elective presentation)	Statins were independently associated with lower odds of AAA rupture at presentation.
	Bahia et al. (2016) [43]	Retrospective cohort (UK primary care database, THIN) with G-computation	AAA	12,485	Statin therapy vs. no statin	Study period 2000–2012	All-cause mortality	Statin use was associated with lower all-cause mortality (HR 0.80, 95% CI 0.77–0.83)
	Skovbo et al. (2025) [34]	Prospective cohort from two population-based screening trials, linked to national registries	Infrarenal AAA (30–55 mm)	998	Statin dose (defined daily doses)	5 years	Aneurysm repair; composite of repair, rupture and all-cause death	Each doubling of statin dose was associated with a lower risk of repair (HR 0.82, 95% CI 0.70–0.97) and of the composite outcome (HR 0.83, 95% CI 0.73–0.94)
**Metformin**	Golledge et al. (2017) [44]	Prospective observational study (three cohorts; ultrasound and CT surveillance)	Infrarenal AAA	1697	Metformin vs. no metformin	Mean 2.9–3.6 years	Combined incidence of AAA repair or rupture-related mortality	Metformin was associated with a reduced likelihood of AAA-related events across all cohorts
	Sutton et al. (2020) [36]	Nationwide retrospective cohort (VA database; multistate model)	Infrarenal AAA	123,440	Metformin-exposed diabetics vs. non-exposed diabetics and non-diabetics	Up to 19 years	AAA surgery; all-cause death; death after surgery	Metformin was associated with a lower risk of death within 10 years after AAA diagnosis and a lower risk of surgery compared with non-diabetics; diabetics without metformin had the highest mortality risk

AAA, abdominal aortic aneurysm; CABG, coronary artery bypass grafting; CAD, coronary artery disease; HR, hazard ratio; PCI, percutaneous coronary intervention; TAA, thoracic aortic aneurysm; VA, Veterans Affairs.

**Table 2:** Studies evaluating the association of statins and metformin with thoracic and abdominal aortic aneurysm growth rates.

**Thoracic aorta (TAA)**										
**Study (year)**		**Design**	**Aortic segment**	***N***	**Age (years), mean**	**Male (%)**	**Baseline diameter (mean)**	**Follow-up**	**Growth rate (mm/year)**	**Main outcome findings**
Jovin et al. (2012) [37]		Retrospective cohort study with multivariable and propensity adjustment	Thoracic aorta (ascending, arch, descending, thoracoabdominal)	649 (214 with ≥2 imaging studies)	70	63	51.6 mm	Median 3.6 years (up to 10 years)	Statin: 0.9 mm/year; No statin: 1.2 mm/year (*p* = 0.005; NS with instrumental-variable estimation)	Lower growth with statins by linear estimation; not significant with instrumental-variable estimation
Stein et al. (2013) [38]		Retrospective cohort study (single-center database)	Thoracic aorta (root, ascending + arch, descending + thoracoabdominal)	1560	62–65	61–65	45 mm	Up to 10 years	No statin: 1.72 mm/year; Statin: 0.88 mm/year (NS)	A trend toward lower aneurysm growth with statins, without statistical significance
Angeloni et al. (2015) [27]		Retrospective propensity-matched cohort study	Ascending aorta	658 (329 statin/329 no statin)	66	~61	39 mm	3 years (100% follow-up)	Statin: 0.95 mm/year; No statin: 1.27 mm/year (*p* < 0.0001)	Statins were associated with a significantly reduced growth rate of the ascending aorta.
**Abdominal aorta (AAA)**										
	**Study (year)**	**Design**	**Aortic segment**	***N***	**Age (years), mean**	**Male (%)**	**Baseline diameter (mean)**	**Follow-up**	**Growth rate (mm/year)**	**Main outcome findings**
**Statins**	Lindholt et al. (2001) [45]	Prospective cohort	Abdominal aorta	117	NR	100	<50 mm	Mean 2.5 y	Mean 2.6	Lipid levels were not associated with expansion
	Schouten et al. (2006) [46]	Retrospective cohort	Infrarenal AAA	150	69	88	Mean 38 mm	Median 3.1 y	Statin: ~2.0; No statin: ~3.6 (*p* = 0.006)	Statin use is associated with lower AAA growth
	Sukhija et al. (2006) [40]	Retrospective cohort	AAA (non-operated)	130	Mean 67	83	Mean 4.5–4.6 cm	Mean 23–24 mo	No growth in statin users; increase in non-users	Statins were associated with the prevention of AAA enlargement
	Mosorin et al. (2008) [41]	Retrospective cohort	Small AAA	121	Mean 70	89	Mean 39 mm	Mean 3.6 y	Statin: 1.9; No statin: 2.6 (NS)	Lower growth with statins
	Schlösser et al. (2008) [47]	Prospective cohort	Small infrarenal AAA	230	Mean 66	90	Mean 38.8–41 mm	Median 3.3 y	−1.2 vs. non-users (*p* ≈ 0.02)	Lipid-lowering therapy (mainly statins) was associated with reduced AAA growth.
	Karlsson et al. (2009) [48]	Prospective cohort	Small AAA	213	Median 71	76.5	Median 4.0 cm	≥18 mo	Statin: 1.6; No statin: 2.5 (*p* = 0.008)	Statin use was associated with significantly lower AAA expansion
	Ferguson et al. (2010) [49]	Multicenter prospective cohort	Small infrarenal AAA	652	Median 73	94	Median 33 mm	Median 5 y	Median 1.1; no difference by statin use (NS)	Statin prescription and LDL levels were not independently associated with AAA growth.
	Sweeting et al. (2010) [50]	Large prospective cohort (UKSAT)	Small infrarenal AAA	1701	Mean ~69	78	Mean 43 mm	Mean 1.9 y	Overall 2.83; statins −0.9 (NS)	Statins showed a nonsignificant trend toward lower growth
	Karrowni et al. (2011) [51]	Retrospective cohort	Small AAA	211	Mean 69–70	67	Mean 4.1 cm	Mean 1.0 y	Statin: 0.9; No statin: 3.2 (*p* < 0.0001)	Statin therapy was associated with markedly lower AAA growth
	Periard et al. (2012) [52]	Prospective cohort	Small infrarenal AAA	94	Mean 69	88	Mean 39.3 mm	Mean 38.5 mo	Statin: 2.91; No statin: 4.37 (*p* = 0.01)	Statin therapy was associated with a significant reduction in AAA expansion
	Skovbo et al. (2025) [34]	Prospective cohort (two population-based screening trials)	Infrarenal AAA (30–55 mm)	998	Median 69.5	100	Median 35.4 mm	5 y	−0.22 per defined daily dose/day (95% CI −0.39 to −0.06)	Higher statin dose was associated with slower growth and fewer aortic events
	Lederle et al. (2015) [53]	Retrospective multicenter cohort (imaging reports from three centers) with propensity analysis	AAA (≥30 mm)	2428	NR	NR	NR	Mean 3.4 y	Overall 2.0; no significant difference by statin use	Statin use was not associated with AAA enlargement after propensity adjustment
**Metformin**	Thompson et al. (2010) [54]	Retrospective analysis of prospectively collected screening data	Infrarenal AAA (≤55 mm)	1231	Median 67 (IQR 65–71)	94	Median 35 (IQR 31–42)	Median 3.2 y (IQR 1.7–6.0)	Median 1.43 (adjusted); ≥2.0 associated with events	Diabetes was independently associated with slower AAA growth.
	Golledge et al. (2017) [44]	Prospective observational study (three cohorts; ultrasound and CT)	Infrarenal AAA (≥30 mm)	1697	Mean ~73	~86	Mean 36.9–40.9	Mean 2.9–3.6 y	Metformin: ~1.0–1.4; No metformin: ~1.6–2.6	Metformin was consistently associated with reduced growth.
	Itoga et al. (2019) [55]	Retrospective cohort (nationwide VA database)	Infrarenal AAA	13,834	Mean 69.8 ± 7.8	99.4	Mean 38.0 ± 7.1	Mean 4.2 ± 2.6 y	Metformin: 1.2 ± 1.9; No metformin: 1.5 ± 2.2 (adjusted −0.20 to −0.23; *p* < 0.001)	Metformin was independently associated with a significantly lower growth rate.
	Unosson et al. (2021) [56]	Prospective multicenter cohort	Small infrarenal AAA	526	Mean 69.1 ± 5.6	95	Mean 38.0 ± 6.1	Mean 3.2 ± 1.7 y	Non-T2DM: 2.3 ± 2.2; T2DM + metformin: 1.1 ± 1.1	Metformin use was independently associated with reduced growth
	Gellatly et al. (2024) [57]	Large prospective cohort (UK Aneurysm Growth Study)	Small infrarenal AAA (≥30 mm)	3670	Mean 69.5	100	Mean 37.3	Median ~5 y	Adjusted −0.38 to −0.56 vs. non-users (*p* < 0.001)	Metformin was strongly and independently associated with slower growth
	Bobadilla-Rosado et al. (2024) [58]	Retrospective observational cohort (CT-based)	Infrarenal AAA	72	Mean 75 ± 9.1	72.2	Median 39.8 (IQR 33.6–55.7)	Median 30 mo (IQR 16–55)	Metformin: stable; No metformin: ≈ +2.2 mm over follow-up	Metformin was associated with diameter stability, whereas non-users showed significant enlargement
	Otify et al. (2026) [35]	Retrospective propensity-matched cohort (AAA screening and surveillance program)	Infrarenal AAA	986	NR	84.7	Mean 39.3 mm	Mean 4.6–5.2 y	Metformin 1.40 (95% CI 1.05–1.88); neither metformin nor ACEi/ARB 1.94 (95% CI 1.78–2.12)	Metformin and ACEi/ARB therapy were independently associated with slower growth, with the greatest effect in combination
	Eilenberg et al. (2025) [29]	Randomized, double-blind, placebo-controlled trial (MetAAA)	Infrarenal AAA (30–49 mm), without diabetes	58	NR	NR	30–49 mm (inclusion range)	18 months	Metformin − placebo 0.16 mm at 12 months (95% CI −0.70 to 1.02)	No significant difference in aneurysm growth; the recruitment target was not reached

AAA, abdominal aortic aneurysm; ACEi, angiotensin-converting enzyme inhibitor; ARB, angiotensin receptor blocker; IQR, interquartile range; LDL, low-density lipoprotein; NR, not reported; NS, not significant; T2DM, type 2 diabetes mellitus; TAA, thoracic aortic aneurysm; UKSAT, UK Small Aneurysm Trial; VA, Veterans Affairs. Mean differences pooled in Figures 3 and 4 were taken as reported by each study (adjusted where available) and may differ from the crude group rates shown.

## Data Availability

The original contributions presented in this study are included in the article and its Supplementary Materials. Further inquiries can be directed to the corresponding authors.

## Supplementary Materials

The following supporting information can be downloaded at: https://www.mdpi.com/article/10.3390/life16101765/s1, File S1: Search strategies; Figure S1: Risk of bias assessment of the included studies (ROBINS-I); Figure S2: Funnel plot for the association between statin use and aneurysm growth; Figure S3: Funnel plot for the association between metformin use and aneurysm growth; Table S1: GRADE assessment of the certainty of evidence and outcome definitions by study; Table S2: Completed PRISMA 2020 checklist.

## Abbreviations

The following abbreviations are used in this manuscript:

AAA	abdominal aortic aneurysm
AAE	acute aortic events
apoB	apolipoprotein B
CI	confidence interval
DM	diabetes mellitus
GRADE	Grading of Recommendations Assessment, Development and Evaluation
HDL-C	high-density lipoprotein cholesterol
HR	hazard ratio
IQR	interquartile range
LDL-C	low-density lipoprotein cholesterol
MD	mean difference
OR	odds ratio
PRISMA	Preferred Reporting Items for Systematic Reviews and Meta-Analyses
REML	restricted maximum likelihood
ROBINS-I	Risk Of Bias In Non-randomized Studies of Interventions
TAA	thoracic aortic aneurysm
