# Supplementary material for: Circulating Lipids, Statins, and Metformin in Thoracic and Abdominal Aortic Aneurysm: A Systematic Review and Meta-Analysis
Source: Life (Basel). 2026 Oct 21;16(10):1765. doi: 10.3390/life16101765 (PMC13706600; doi:10.3390/life16101765)
Supplement: Supplementary file 1 [file life-16-01765-s001.zip › File_S1_Search_strategies.pdf]

File S1. Search strategies

Search strategies were run on 24 March 2025.

The search was updated in Ovid MEDLINE on 3 October 2026 using the same concepts and the same eligibility criteria; it retrieved 110 additional records, which were screened in duplicate. The updated MEDLINE search is reported in the main article (Section 2.1 and Figure 1).

Ovid MEDLINE(R) <1946 to March 21, 2025>

#	Searches	Results	Type
1	exp Hypolipidemic Agents/	171886	Advanced
2	exp Cholesterol/	179877	Advanced
3	exp Hyperlipidemias/	72524	Advanced
4	antihyperlipemic*.mp.	23	Advanced
5	Antilip?emic*.mp.	331	Advanced
6	(hypolipidemic adj2 (agent? or drug?)).mp.	17399	Advanced
7	(anticholesteremic adj2 (agent? or drug?)).mp.	17757	Advanced
8	(antihypertriglyceridic adj2 (agent? or drug?)).mp.	0	Advanced
9	(antilip?emia adj2 (agent? or drug?)).mp.	0	Advanced
10	(antilipidemic adj2 (agent? or drug?)).mp.	87	Advanced
11	(hypolip?emic adj2 (agent? or drug?)).mp.	166	Advanced
12	(hypotriglyceridic adj2 (agent? or drug?)).mp.	0	Advanced
13	(lipid depressing adj2 (agent? or drug?)).mp.	0	Advanced
14	(lipid lowering adj2 (agent? or drug?)).mp.	5987	Advanced
15	Anticholesteremic?.mp.	17767	Advanced
16	(cholesterol adj2 inhibitor?).mp.	1603	Advanced
17	(hypocholester?emic adj2 (agent? or drug?)).mp.	72	Advanced
18	(Hypocholesterol?emic adj2 (agent? or drug?)).mp.	490	Advanced

19	(antihypercholesteremic adj2 (agent? or drug?)).mp.	2	Advanced
20	hmg coa reductase inhibitor?.mp.	4212	Advanced
21	hmg coenzyme a reductase inhibitor?.mp.	43	Advanced
22	hydroxymethylglutaryl coa reductase inhibitor?.mp.	36590	Advanced
23	hydroxymethylglutaryl-coa inhibitor?.mp.	4	Advanced
24	hydroxymethylglutaryl-coenzyme a inhibitor?.mp.	8	Advanced
25	statin?.mp.	48914	Advanced
26	pcsk9 inhibitor?.mp.	2322	Advanced
27	fatty acid synthesis inhibitor?.mp.	135	Advanced
28	lipocaic?.mp.	98	Advanced
29	(lipotropic adj2 (agent? or drug? or factor?)).mp.	1001	Advanced
30	Cholesterol.mp.	313261	Advanced
31	Epicholesterol.mp.	80	Advanced
32	Hyperlip?emia?.mp.	2872	Advanced
33	Hyperlipid?emia?.mp.	49608	Advanced
34	Lipemia?.mp.	2100	Advanced
35	Lipid?emia?.mp.	464	Advanced
36	Hypercholester?emia?.mp.	1321	Advanced
37	Hypercholesterol?emia?.mp.	47907	Advanced
38	Hypercholesterin?emia.mp.	192	Advanced
39	Hypertriglycerid?emia?.mp.	16599	Advanced
40	Cholesteremia.mp.	74	Advanced
41	Cholesterinemia.mp.	38	Advanced
42	Cholesterolemia.mp.	1054	Advanced
43	Atorvastatin.mp.	10441	Advanced
44	a 2581175.mp.	0	Advanced

45	a2581175.mp.	0	Advanced
46	amicor.mp.	0	Advanced
47	antorcin.mp.	0	Advanced
48	apo−atorva.mp.	0	Advanced
49	arkas.mp.	2	Advanced
50	artas.mp.	44	Advanced
51	ascord.mp.	0	Advanced
52	astator.mp.	0	Advanced
53	atacor.mp.	1	Advanced
54	atofast.mp.	0	Advanced
55	ator.mp.	124	Advanced
56	ator−chol.mp.	0	Advanced
57	atorab.mp.	0	Advanced
58	atorbir.mp.	0	Advanced
59	atorcor.mp.	0	Advanced
60	atoridor.mp.	0	Advanced
61	atorin.mp.	0	Advanced
62	atoris.mp.	1	Advanced
63	atorlip.mp.	0	Advanced
64	atormax.mp.	0	Advanced
65	atorstad.mp.	0	Advanced
66	atorstan.mp.	0	Advanced
67	atorstat.mp.	0	Advanced
68	atorvadivid.mp.	0	Advanced
69	atorvagen.mp.	0	Advanced
70	atorval.mp.	0	Advanced

71	atorvalan.mp.	0	Advanced
72	atorvalet.mp.	0	Advanced
73	atorvaliq.mp.	0	Advanced
74	atorvanox.mp.	0	Advanced
75	atorvasterol.mp.	0	Advanced
76	atorvastine.mp.	0	Advanced
77	atorvox.mp.	0	Advanced
78	atorzem.mp.	0	Advanced
79	atostat.mp.	0	Advanced
80	atostin.mp.	0	Advanced
81	atovans.mp.	0	Advanced
82	atovarol.mp.	0	Advanced
83	atractin.mp.	1	Advanced
84	atraven.mp.	0	Advanced
85	atrocell.mp.	0	Advanced
86	atrost.mp.	0	Advanced
87	atrox.mp.	841	Advanced
88	calipra.mp.	0	Advanced
89	card−ok.mp.	0	Advanced
90	cardiostyl.mp.	0	Advanced
91	cardyl.mp.	0	Advanced
92	ci 981.mp.	177	Advanced
93	ci981.mp.	1	Advanced
94	decholest.mp.	0	Advanced
95	delipost.mp.	0	Advanced
96	gletor.mp.	0	Advanced

97	glustar.mp.	0	Advanced
98	lambrinex.mp.	0	Advanced
99	larus.mp.	1080	Advanced
100	latrovin.mp.	0	Advanced
101	lipibec.mp.	0	Advanced
102	lipigan.mp.	0	Advanced
103	lipimed.mp.	0	Advanced
104	lipistad.mp.	0	Advanced
105	lipitor.mp.	203	Advanced
106	lipovast.mp.	0	Advanced
107	liprimar.mp.	4	Advanced
108	liptin.mp.	2	Advanced
109	liptonorm.mp.	2	Advanced
110	lorvaten.mp.	0	Advanced
111	lowlipen.mp.	0	Advanced
112	nelibat.mp.	0	Advanced
113	obradon.mp.	0	Advanced
114	omegastatin.mp.	0	Advanced
115	orbeos.mp.	0	Advanced
116	orvasta.mp.	0	Advanced
117	pd 13429838a.mp.	0	Advanced
118	pd13429838a.mp.	0	Advanced
119	prevencor.mp.	0	Advanced
120	provicard.mp.	0	Advanced
121	rafitin.mp.	0	Advanced
122	rotacor.mp.	1	Advanced

123	rotova.mp.	0	Advanced
124	"s 05153".mp.	0	Advanced
125	s05153.mp.	0	Advanced
126	sortis.mp.	35	Advanced
127	statorva.mp.	0	Advanced
128	storvas.mp.	2	Advanced
129	tahor.mp.	1	Advanced
130	thervan.mp.	0	Advanced
131	torvacard.mp.	0	Advanced
132	torvachol.mp.	0	Advanced
133	torvacol.mp.	0	Advanced
134	torvalipin.mp.	0	Advanced
135	torvaplus.mp.	0	Advanced
136	torvarin.mp.	0	Advanced
137	torvas.mp.	0	Advanced
138	torvast.mp.	0	Advanced
139	torvastin.mp.	0	Advanced
140	torvatec.mp.	0	Advanced
141	torvazin.mp.	0	Advanced
142	totalip.mp.	0	Advanced
143	tulip.mp.	668	Advanced
144	vastat.mp.	0	Advanced
145	vastatin.mp.	15	Advanced
146	vastazor.mp.	0	Advanced
147	voredanin.mp.	0	Advanced
148	xarator.mp.	0	Advanced

149	xolister−all.mp.	0	Advanced
150	ym 548.mp.	0	Advanced
151	ym548.mp.	0	Advanced
152	zarastin.mp.	0	Advanced
153	zarator.mp.	0	Advanced
154	coupet.mp.	0	Advanced
155	crestor.mp.	70	Advanced
156	epri.mp.	180	Advanced
157	ezallor.mp.	5	Advanced
158	"hgp 0816".mp.	0	Advanced
159	hgp0816.mp.	0	Advanced
160	mertenil.mp.	3	Advanced
161	provisacor.mp.	0	Advanced
162	ropitor.mp.	1	Advanced
163	rostat.mp.	0	Advanced
164	rosudia.mp.	0	Advanced
165	rosumop.mp.	0	Advanced
166	rosuvador.mp.	0	Advanced
167	rosuvas.mp.	3	Advanced
168	rosuvastatin.mp.	4316	Advanced
169	rosuvastatina.mp.	12	Advanced
170	rosuvastatine.mp.	19	Advanced
171	roswera.mp.	0	Advanced
172	roxera.mp.	0	Advanced
173	rozuva−teva.mp.	0	Advanced
174	s 4522.mp.	1	Advanced

175	s4522.mp.	1	Advanced
176	simestat.mp.	0	Advanced
177	sorvasta.mp.	0	Advanced
178	visacor.mp.	14	Advanced
179	xeter.mp.	0	Advanced
180	zahron.mp.	0	Advanced
181	zaranta.mp.	0	Advanced
182	zd 4522.mp.	0	Advanced
183	zd4522.mp.	5	Advanced
184	alipza.mp.	0	Advanced
185	itavastatin.mp.	7	Advanced
186	lippiza.mp.	0	Advanced
187	livalo.mp.	21	Advanced
188	livazo.mp.	2	Advanced
189	nikita.mp.	20	Advanced
190	nisvastatin.mp.	8	Advanced
191	nk 104.mp.	39	Advanced
192	nk104.mp.	0	Advanced
193	nks 104.mp.	2	Advanced
194	nks104.mp.	0	Advanced
195	p 872441.mp.	2	Advanced
196	p872441.mp.	0	Advanced
197	pitava.mp.	6	Advanced
198	pitavastatin.mp.	1136	Advanced
199	redevant.mp.	0	Advanced
200	ribar.mp.	7	Advanced

201	trolise.mp.	0	Advanced
202	vezepra.mp.	0	Advanced
203	zypitamag.mp.	6	Advanced
204	aplactin.mp.	0	Advanced
205	astin.mp.	39	Advanced
206	bristacol.mp.	0	Advanced
207	cholespar.mp.	0	Advanced
208	cs 514.mp.	35	Advanced
209	cs514.mp.	0	Advanced
210	dehypotin protect.mp.	0	Advanced
211	elisor.mp.	3	Advanced
212	epatostantin.mp.	0	Advanced
213	eptastatin.mp.	5	Advanced
214	eptastatine.mp.	0	Advanced
215	kenstatin.mp.	0	Advanced
216	lipemol.mp.	0	Advanced
217	lipidal.mp.	0	Advanced
218	liplat.mp.	0	Advanced
219	lipostat.mp.	28	Advanced
220	liprevil.mp.	0	Advanced
221	maxudin.mp.	0	Advanced
222	mevalotin.mp.	8	Advanced
223	minuscol.mp.	0	Advanced
224	novales.mp.	1	Advanced
225	prareduct.mp.	0	Advanced
226	prascolend.mp.	0	Advanced

227	prastan.mp.	0	Advanced
228	prasterol.mp.	0	Advanced
229	prava.mp.	92	Advanced
230	pravachol.mp.	30	Advanced
231	pravacol.mp.	1	Advanced
232	pravalam.mp.	0	Advanced
233	pravalich.mp.	0	Advanced
234	pravaselect.mp.	0	Advanced
235	pravasin.mp.	0	Advanced
236	pravasine.mp.	0	Advanced
237	pravastatin.mp.	4970	Advanced
238	pravastatine.mp.	25	Advanced
239	pravator.mp.	0	Advanced
240	pravyl.mp.	1	Advanced
241	rms 431.mp.	1	Advanced
242	sanaprav.mp.	0	Advanced
243	selectin.mp.	23516	Advanced
244	selektine.mp.	1	Advanced
245	selipran.mp.	0	Advanced
246	sq 31000.mp.	1	Advanced
247	sq31000.mp.	0	Advanced
248	stanidine.mp.	0	Advanced
249	vasopran.mp.	0	Advanced
250	vasten.mp.	7	Advanced
251	versatab.mp.	0	Advanced
252	xipral.mp.	0	Advanced

253	alcosin.mp.	0	Advanced
254	antichol.mp.	0	Advanced
255	apo−simva.mp.	0	Advanced
256	avastinee.mp.	0	Advanced
257	belmalip.mp.	0	Advanced
258	cholemed.mp.	0	Advanced
259	cholestat.mp.	0	Advanced
260	clinfar.mp.	0	Advanced
261	colastatina.mp.	0	Advanced
262	coledis.mp.	0	Advanced
263	colemin.mp.	0	Advanced
264	colemin forte.mp.	0	Advanced
265	colestricon.mp.	0	Advanced
266	corolin.mp.	0	Advanced
267	corsim.mp.	2	Advanced
268	covastin.mp.	0	Advanced
269	denan.mp.	2	Advanced
270	epistatin.mp.	1	Advanced
271	esvat.mp.	0	Advanced
272	eucor.mp.	1	Advanced
273	flolipid.mp.	6	Advanced
274	glipal.mp.	0	Advanced
275	ifistatin.mp.	0	Advanced
276	ipramid.mp.	0	Advanced
277	jabastatina.mp.	0	Advanced
278	kavelor.mp.	0	Advanced

279	klonastin.mp.	0	Advanced
280	kolestevan.mp.	0	Advanced
281	kymazol.mp.	0	Advanced
282	l 644128.mp.	0	Advanced
283	l644128.mp.	0	Advanced
284	labistatin.mp.	0	Advanced
285	lepur.mp.	0	Advanced
286	lip−down.mp.	12	Advanced
287	lipaxin.mp.	0	Advanced
288	lipcut.mp.	0	Advanced
289	lipecor.mp.	0	Advanced
290	lipex.mp.	11	Advanced
291	lipinorm.mp.	0	Advanced
292	lipomin.mp.	2	Advanced
293	liponorm.mp.	1	Advanced
294	liporex.mp.	0	Advanced
295	lipovas.mp.	0	Advanced
296	lipterra.mp.	0	Advanced
297	lodales.mp.	1	Advanced
298	lowcholid.mp.	0	Advanced
299	medipo.mp.	3	Advanced
300	medistatin.mp.	0	Advanced
301	mersivas.mp.	0	Advanced
302	"mk 0733".mp.	0	Advanced
303	mk 733.mp.	39	Advanced
304	mk0733.mp.	0	Advanced

305	mk733.mp.	1	Advanced
306	nezatin.mp.	0	Advanced
307	nitastin.mp.	0	Advanced
308	nivelipol.mp.	0	Advanced
309	nor−vastina.mp.	0	Advanced
310	normofat.mp.	1	Advanced
311	nyzoc.mp.	0	Advanced
312	omistat.mp.	0	Advanced
313	orovas.mp.	0	Advanced
314	pantok.mp.	0	Advanced
315	placol.mp.	2	Advanced
316	pravostin.mp.	0	Advanced
317	priacin.mp.	0	Advanced
318	protecta.mp.	8	Advanced
319	ranzolont.mp.	0	Advanced
320	raptor.mp.	2073	Advanced
321	rechol.mp.	0	Advanced
322	redusterol.mp.	0	Advanced
323	rendapid.mp.	0	Advanced
324	simar.mp.	12	Advanced
325	simbado.mp.	0	Advanced
326	simbatrix.mp.	0	Advanced
327	simcard.mp.	3	Advanced
328	simchol.mp.	0	Advanced
329	simcovas.mp.	0	Advanced
330	simorion.mp.	0	Advanced

331	simovil.mp.	1	Advanced
332	simratio.mp.	0	Advanced
333	simtan.mp.	0	Advanced
334	simtin.mp.	0	Advanced
335	simva.mp.	60	Advanced
336	simvabeta.mp.	0	Advanced
337	simvacard.mp.	0	Advanced
338	simvachol.mp.	0	Advanced
339	simvacol.mp.	0	Advanced
340	simvacor.mp.	0	Advanced
341	simvador.mp.	1	Advanced
342	simvadura.mp.	0	Advanced
343	simvagen.mp.	0	Advanced
344	simvahex.mp.	0	Advanced
345	simvahexal.mp.	0	Advanced
346	simvalid.mp.	0	Advanced
347	simvalimit.mp.	0	Advanced
348	simvalord.mp.	0	Advanced
349	simvasim.mp.	0	Advanced
350	simvastar.mp.	0	Advanced
351	simvastatin.mp.	11446	Advanced
352	simvastatina.mp.	55	Advanced
353	simvastatine.mp.	59	Advanced
354	simvasterol.mp.	0	Advanced
355	simvata.mp.	0	Advanced
356	simvatin.mp.	2	Advanced

357	simvax.mp.	0	Advanced
358	simvor.mp.	1	Advanced
359	simvotin.mp.	1	Advanced
360	sincol.mp.	0	Advanced
361	sinpor.mp.	0	Advanced
362	sintenal.mp.	0	Advanced
363	sinvacor.mp.	0	Advanced
364	sinvalip.mp.	0	Advanced
365	sinvastatin.mp.	7	Advanced
366	sinvinolin.mp.	0	Advanced
367	sivastin.mp.	0	Advanced
368	sivatin.mp.	0	Advanced
369	starzoco.mp.	0	Advanced
370	stasiva.mp.	0	Advanced
371	statinum.mp.	0	Advanced
372	stativer.mp.	0	Advanced
373	synvinolin.mp.	19	Advanced
374	torio.mp.	18	Advanced
375	tremital.mp.	0	Advanced
376	valemia.mp.	0	Advanced
377	vasilip.mp.	7	Advanced
378	vasotenal.mp.	0	Advanced
379	vastan.mp.	4	Advanced
380	vastatin.mp.	15	Advanced
381	vastin.mp.	0	Advanced
382	vazim.mp.	0	Advanced

383	velastatin.mp.	0	Advanced
384	velkastatin.mp.	0	Advanced
385	velostatin.mp.	0	Advanced
386	veristin.mp.	0	Advanced
387	veritrat.mp.	0	Advanced
388	vidastat.mp.	0	Advanced
389	ximve.mp.	0	Advanced
390	xipocol.mp.	0	Advanced
391	zeplan.mp.	0	Advanced
392	zimmex.mp.	0	Advanced
393	zocor.mp.	116	Advanced
394	zocord.mp.	0	Advanced
395	zorced.mp.	0	Advanced
396	zosta.mp.	0	Advanced
397	zovar.mp.	0	Advanced
398	zovast.mp.	0	Advanced
399	altocor.mp.	0	Advanced
400	altoprev.mp.	9	Advanced
401	artein.mp.	0	Advanced
402	belvas.mp.	0	Advanced
403	birotin.mp.	0	Advanced
404	cholestra.mp.	2	Advanced
405	cysin.mp.	0	Advanced
406	ellanco.mp.	0	Advanced
407	elstatin.mp.	0	Advanced
408	l 654969.mp.	0	Advanced

409	lipdip.mp.	0	Advanced
410	lipivas.mp.	0	Advanced
411	lofacol.mp.	0	Advanced
412	lomar.mp.	0	Advanced
413	lostatin.mp.	0	Advanced
414	lovacel.mp.	0	Advanced
415	lovacol.mp.	0	Advanced
416	lovahexal.mp.	0	Advanced
417	lovalip.mp.	0	Advanced
418	lovalord.mp.	0	Advanced
419	lovastan.mp.	0	Advanced
420	lovastatin.mp.	6087	Advanced
421	lovasterol.mp.	0	Advanced
422	lovastin.mp.	5	Advanced
423	lovatadin.mp.	0	Advanced
424	lowachol.mp.	0	Advanced
425	lozutin.mp.	0	Advanced
426	medostatin.mp.	0	Advanced
427	mevacor.mp.	45	Advanced
428	meverstin.mp.	0	Advanced
429	mevinacor.mp.	2	Advanced
430	"mk 0803".mp.	0	Advanced
431	mk 803.mp.	6	Advanced
432	mk0803.mp.	0	Advanced
433	mk803.mp.	1	Advanced
434	monacolin K.mp.	230	Advanced

435	monakolin k.mp.	3	Advanced
436	msd 803.mp.	0	Advanced
437	neolipid.mp.	5	Advanced
438	nergadan.mp.	0	Advanced
439	ovasta.mp.	0	Advanced
440	rodatin.mp.	0	Advanced
441	rovacor.mp.	0	Advanced
442	taucor.mp.	1	Advanced
443	almastatin.mp.	0	Advanced
444	canef.mp.	0	Advanced
445	cardiol.mp.	518	Advanced
446	cranoc.mp.	0	Advanced
447	digardil.mp.	0	Advanced
448	digaril.mp.	0	Advanced
449	fluindostatin.mp.	4	Advanced
450	fluvastatin.mp.	2134	Advanced
451	fluvastatina.mp.	6	Advanced
452	fluvastatine.mp.	9	Advanced
453	fractal.mp.	8044	Advanced
454	leposit prolib.mp.	0	Advanced
455	lescol.mp.	83	Advanced
456	leucol.mp.	0	Advanced
457	lipaxan.mp.	0	Advanced
458	lipaxin.mp.	0	Advanced
459	liposit.mp.	0	Advanced
460	lochol.mp.	1	Advanced

461	locol.mp.	4	Advanced
462	lymetel.mp.	0	Advanced
463	primesin.mp.	0	Advanced
464	primexin.mp.	0	Advanced
465	sri 62320.mp.	1	Advanced
466	sri62320.mp.	0	Advanced
467	vaditon.mp.	0	Advanced
468	vaditon prolib.mp.	0	Advanced
469	vastin.mp.	0	Advanced
470	xu 62 320.mp.	8	Advanced
471	xu 62320.mp.	0	Advanced
472	xu62320.mp.	0	Advanced
473	anj 900.mp.	0	Advanced
474	anj900.mp.	0	Advanced
475	apophage.mp.	0	Advanced
476	aron.mp.	136	Advanced
477	benofomin.mp.	0	Advanced
478	dabex.mp.	0	Advanced
479	denkaform.mp.	0	Advanced
480	deson.mp.	0	Advanced
481	dextin.mp.	0	Advanced
482	diabetase.mp.	0	Advanced
483	diabetase s.mp.	0	Advanced
484	diabetformin.mp.	0	Advanced
485	diabetmin.mp.	2	Advanced
486	diabetosan.mp.	1	Advanced

487	diabex.mp.	3	Advanced
488	diafat.mp.	0	Advanced
489	diaformin.mp.	1	Advanced
490	diaformina.mp.	0	Advanced
491	diametin.mp.	0	Advanced
492	diamin.mp.	28	Advanced
493	dianben.mp.	0	Advanced
494	diformin.mp.	4	Advanced
495	dimefor.mp.	0	Advanced
496	dimethylbiguanide.mp.	141	Advanced
497	dimethyldiguanide.mp.	12	Advanced
498	dmgg.mp.	1	Advanced
499	dybis.mp.	0	Advanced
500	"efb 0027".mp.	0	Advanced
501	efb0027.mp.	0	Advanced
502	eraphage.mp.	0	Advanced
503	espa–formin.mp.	0	Advanced
504	euform retard.mp.	0	Advanced
505	fluamine.mp.	0	Advanced
506	flumamine.mp.	2	Advanced
507	fornidd.mp.	0	Advanced
508	fortamet.mp.	0	Advanced
509	glafornil.mp.	0	Advanced
510	glibudon.mp.	0	Advanced
511	glifage.mp.	0	Advanced
512	gliguanid.mp.	0	Advanced

513	glucaminol.mp.	0	Advanced
514	glucofage.mp.	0	Advanced
515	glucofago.mp.	0	Advanced
516	glucoform.mp.	0	Advanced
517	glucoformin.mp.	0	Advanced
518	glucohexal.mp.	0	Advanced
519	glucoless.mp.	0	Advanced
520	glucomet.mp.	1	Advanced
521	glucomin.mp.	0	Advanced
522	glucomine.mp.	0	Advanced
523	gluconil.mp.	0	Advanced
524	glucophage.mp.	134	Advanced
525	glucostop.mp.	0	Advanced
526	glucotika.mp.	0	Advanced
527	gludepatic.mp.	0	Advanced
528	glufor.mp.	0	Advanced
529	gluformin.mp.	1	Advanced
530	glukophage.mp.	0	Advanced
531	glumeformin.mp.	0	Advanced
532	glumet.mp.	2	Advanced
533	glumetza.mp.	10	Advanced
534	glupa.mp.	0	Advanced
535	glustress.mp.	0	Advanced
536	glyciphage.mp.	1	Advanced
537	glycomet.mp.	1	Advanced
538	glycon.mp.	98	Advanced

539	glycoran.mp.	0	Advanced
540	glyformin.mp.	1	Advanced
541	glymet.mp.	8	Advanced
542	haurymellin.mp.	0	Advanced
543	hipoglucin.mp.	0	Advanced
544	i−max.mp.	278	Advanced
545	islotin.mp.	0	Advanced
546	jesacrin.mp.	0	Advanced
547	juformin.mp.	0	Advanced
548	la 6023.mp.	10	Advanced
549	la6023.mp.	0	Advanced
550	lyomet.mp.	0	Advanced
551	maformin.mp.	0	Advanced
552	meglucon.mp.	0	Advanced
553	meguan.mp.	3	Advanced
554	melbin.mp.	2	Advanced
555	melformin.mp.	1	Advanced
556	mellittin.mp.	1	Advanced
557	merckformin.mp.	0	Advanced
558	mescorit.mp.	0	Advanced
559	metaformin.mp.	6	Advanced
560	metfogamma.mp.	0	Advanced
561	metfoliquid geriasan.mp.	0	Advanced
562	metforal.mp.	0	Advanced
563	metformax.mp.	2	Advanced
564	metformin.mp.	27435	Advanced

565	metformina.mp.	163	Advanced
566	metformine.mp.	251	Advanced
567	methformin.mp.	1	Advanced
568	metiguanide.mp.	1	Advanced
569	metomin.mp.	0	Advanced
570	metphormin.mp.	0	Advanced
571	miformin.mp.	0	Advanced
572	dimethylguanylguanide.mp.	0	Advanced
573	dimethylguanylguanidine.mp.	4	Advanced
574	dimethyldiguanide.mp.	12	Advanced
575	dimethyl biguanidine.mp.	0	Advanced
576	dimethylbiguanide.mp.	141	Advanced
577	dimethylbiguanidine.mp.	4	Advanced
578	dimethyldiguanide.mp.	12	Advanced
579	dimethylguanylguanidine.mp.	4	Advanced
580	neoform.mp.	5	Advanced
581	newmet.mp.	0	Advanced
582	nndg.mp.	0	Advanced
583	reglus−500.mp.	0	Advanced
584	riomet.mp.	5	Advanced
585	riomet er.mp.	0	Advanced
586	risidon.mp.	0	Advanced
587	rudimet.mp.	0	Advanced
588	siamformet.mp.	0	Advanced
589	siofor.mp.	4	Advanced
590	thiabet.mp.	0	Advanced

591	vimetrol.mp.	0	Advanced
592	walaphage.mp.	0	Advanced
593	absorcol.mp.	0	Advanced
594	adezop.mp.	0	Advanced
595	azibe.mp.	0	Advanced
596	carditimib.mp.	0	Advanced
597	cexado.mp.	0	Advanced
598	cildar.mp.	0	Advanced
599	coltowan.mp.	0	Advanced
600	corintus.mp.	0	Advanced
601	delipid.mp.	4	Advanced
602	egitim.mp.	83	Advanced
603	elanix.mp.	0	Advanced
604	erezel.mp.	0	Advanced
605	esetin.mp.	0	Advanced
606	etibax.mp.	0	Advanced
607	ezdivule.mp.	0	Advanced
608	ezedoc.mp.	0	Advanced
609	ezegelan.mp.	0	Advanced
610	ezegros.mp.	0	Advanced
611	ezehron.mp.	0	Advanced
612	ezelip.mp.	0	Advanced
613	ezemed.mp.	0	Advanced
614	ezen.mp.	297	Advanced
615	ezetad.mp.	0	Advanced
616	ezetebmib.mp.	0	Advanced

617	ezetib.mp.	0	Advanced
618	ezetiben.mp.	0	Advanced
619	ezetimib.mp.	56	Advanced
620	ezetimibe.mp.	4191	Advanced
621	ezetrol.mp.	23	Advanced
622	ezetymib.mp.	1	Advanced
623	ezgal.mp.	0	Advanced
624	ezicor.mp.	0	Advanced
625	ezoleta.mp.	0	Advanced
626	hgp 1404.mp.	0	Advanced
627	hgp1404.mp.	0	Advanced
628	intestat.mp.	0	Advanced
629	kobey.mp.	0	Advanced
630	lipegis.mp.	0	Advanced
631	lipobon.mp.	0	Advanced
632	mibecol.mp.	0	Advanced
633	mibezet.mp.	0	Advanced
634	noveze.mp.	0	Advanced
635	noxetib.mp.	0	Advanced
636	olufsent.mp.	0	Advanced
637	quver.mp.	2	Advanced
638	sch 58235.mp.	16	Advanced
639	sch58235.mp.	4	Advanced
640	strami.mp.	1	Advanced
641	symezet.mp.	0	Advanced
642	tezzimi.mp.	0	Advanced

643	tritilz.mp.	0	Advanced
644	trizibe.mp.	0	Advanced
645	viemm.mp.	0	Advanced
646	vyeve.mp.	0	Advanced
647	ylerse.mp.	0	Advanced
648	zertya.mp.	0	Advanced
649	zetaxyl.mp.	0	Advanced
650	zetia.mp.	49	Advanced
651	zetiraf.mp.	0	Advanced
652	zetratak.mp.	0	Advanced
653	zetratak−s.mp.	0	Advanced
654	zient.mp.	6	Advanced
655	alirocumab.mp.	899	Advanced
656	praluent.mp.	40	Advanced
657	regn 727.mp.	2	Advanced
658	regn727.mp.	22	Advanced
659	sar 236553.mp.	1	Advanced
660	sar236553.mp.	18	Advanced
661	evolucumab.mp.	6	Advanced
662	amg 145.mp.	32	Advanced
663	amg145.mp.	9	Advanced
664	repatha.mp.	40	Advanced
665	"sal 003".mp.	3	Advanced
666	sal003.mp.	11	Advanced
667	aln 60212.mp.	1	Advanced
668	aln pcssc.mp.	4	Advanced

669 aln60212.mp.	0	Advanced
670 inclisiran.mp.	332	Advanced
671 kjx 839.mp.	1	Advanced
672 kjx839.mp.	0	Advanced
673 leqvio.mp.	14	Advanced
674 antara.mp.	14	Advanced
675 apo−feno−micro.mp.	0	Advanced
676 apteor.mp.	0	Advanced
677 aterolis.mp.	0	Advanced
678 biofibrat.mp.	0	Advanced
679 bisterol sr.mp.	0	Advanced
680 catalip.mp.	0	Advanced
681 climage.mp.	1	Advanced
682 controlip.mp.	1	Advanced
683 durafenat.mp.	0	Advanced
684 elipsia.mp.	0	Advanced
685 evothyl.mp.	0	Advanced
686 febira.mp.	0	Advanced
687 fegenor.mp.	0	Advanced
688 felosma.mp.	0	Advanced
689 fenardin.mp.	0	Advanced
690 fenobrat.mp.	0	Advanced
691 fenobrate.mp.	1	Advanced
692 fenochol.mp.	0	Advanced
693 fenofanton.mp.	0	Advanced
694 fenofibrate.mp.	4062	Advanced

695	fenofix.mp.	0	Advanced
696	fenogal.mp.	1	Advanced
697	fenoglide.mp.	3	Advanced
698	fenolip.mp.	0	Advanced
699	fenomax.mp.	0	Advanced
700	fenosup lidose.mp.	0	Advanced
701	fenoswiss.mp.	0	Advanced
702	fenox.mp.	4	Advanced
703	fibrafen.mp.	0	Advanced
704	fulcro.mp.	5	Advanced
705	fulcrosupra.mp.	0	Advanced
706	grofibrat.mp.	0	Advanced
707	"grs 001".mp.	0	Advanced
708	grs001.mp.	0	Advanced
709	hyperchol.mp.	3	Advanced
710	katalip.mp.	1	Advanced
711	lexemin.mp.	0	Advanced
712	lifenoz−u.mp.	0	Advanced
713	lipanthyl.mp.	63	Advanced
714	lipanthyl.mp.	63	Advanced
715	lipanthylnano.mp.	2	Advanced
716	lipantil.mp.	12	Advanced
717	lipantyl.mp.	1	Advanced
718	liparison.mp.	0	Advanced
719	lipcor.mp.	0	Advanced
720	liperial.mp.	0	Advanced

721	lipidax.mp.	0	Advanced
722	lipidil.mp.	12	Advanced
723	lipilfen.mp.	1	Advanced
724	lipilo.mp.	1	Advanced
725	lipirex.mp.	0	Advanced
726	lipoclar.mp.	0	Advanced
727	lipofen.mp.	5	Advanced
728	lipofene.mp.	0	Advanced
729	lipofib.mp.	0	Advanced
730	lipolin.mp.	0	Advanced
731	liposit.mp.	0	Advanced
732	lipsin.mp.	0	Advanced
733	livesan ge.mp.	0	Advanced
734	lofibra.mp.	0	Advanced
735	nopid 200.mp.	0	Advanced
736	normalip.mp.	1	Advanced
737	nubrex.mp.	0	Advanced
738	procetofen.mp.	20	Advanced
739	procetofenate.mp.	0	Advanced
740	procetofene.mp.	28	Advanced
741	procetofen.mp.	20	Advanced
742	qualipantyl.mp.	0	Advanced
743	rapidil.mp.	0	Advanced
744	redose 200.mp.	0	Advanced
745	rorit.mp.	0	Advanced
746	rp 1824.mp.	0	Advanced

747	rp1824.mp.	0	Advanced
748	secalip.mp.	2	Advanced
749	sigurtil.mp.	0	Advanced
750	supralip.mp.	0	Advanced
751	suprelip.mp.	0	Advanced
752	trichol.mp.	0	Advanced
753	tricor.mp.	22	Advanced
754	triglide.mp.	5	Advanced
755	trolip.mp.	0	Advanced
756	xafenor.mp.	0	Advanced
757	zerlubron.mp.	0	Advanced
758	ziglip.mp.	0	Advanced
759	zigotrig.mp.	0	Advanced
760	zumafib.mp.	0	Advanced
761	ausgem.mp.	0	Advanced
762	bolutol.mp.	0	Advanced
763	brozil.mp.	0	Advanced
764	chlorestrol.mp.	0	Advanced
765	cholespid.mp.	0	Advanced
766	ci 719.mp.	207	Advanced
767	ci719.mp.	5	Advanced
768	clearol.mp.	0	Advanced
769	decrelip.mp.	0	Advanced
770	detrichol.mp.	0	Advanced
771	elmogan.mp.	0	Advanced
772	fetinor.mp.	0	Advanced

773	fibralip.mp.	0	Advanced
774	fibrocit.mp.	0	Advanced
775	gedum.mp.	0	Advanced
776	gemfi.mp.	0	Advanced
777	gemfibril.mp.	0	Advanced
778	gemfibromax.mp.	0	Advanced
779	gemfibrozil.mp.	2225	Advanced
780	gemfibrozil.mp.	2225	Advanced
781	gemfibrozilo.mp.	10	Advanced
782	gemhexal.mp.	0	Advanced
783	gemizol.mp.	0	Advanced
784	gemlipid.mp.	0	Advanced
785	gemnpid.mp.	0	Advanced
786	gemzil.mp.	0	Advanced
787	genfibrozil.mp.	4	Advanced
788	gevilon.mp.	13	Advanced
789	gevilon uno.mp.	0	Advanced
790	gozid.mp.	0	Advanced
791	grifogemzilo.mp.	0	Advanced
792	hidil.mp.	1	Advanced
793	hipolixan.mp.	0	Advanced
794	ipolipid.mp.	0	Advanced
795	jezil.mp.	0	Advanced
796	lanaterom.mp.	0	Advanced
797	lifibron.mp.	0	Advanced
798	lipazil.mp.	0	Advanced

799	lipidys.mp.	0	Advanced
800	lipigem.mp.	0	Advanced
801	lipira.mp.	0	Advanced
802	lipison.mp.	0	Advanced
803	lipistorol.mp.	0	Advanced
804	lipizyl.mp.	0	Advanced
805	lipofor.mp.	0	Advanced
806	lipolo.mp.	0	Advanced
807	lipostorol.mp.	0	Advanced
808	lipox gemfi.mp.	0	Advanced
809	lipozid.mp.	0	Advanced
810	lipozil.mp.	0	Advanced
811	lipur.mp.	3	Advanced
812	litarek.mp.	0	Advanced
813	lopid.mp.	46	Advanced
814	lowin.mp.	5	Advanced
815	low−lip.mp.	15	Advanced
816	manobrozil.mp.	0	Advanced
817	mariston.mp.	0	Advanced
818	mersikol.mp.	0	Advanced
819	normolip.mp.	1	Advanced
820	panazil.mp.	0	Advanced
821	pilder.mp.	3	Advanced
822	polyxit.mp.	0	Advanced
823	progemzal.mp.	0	Advanced
824	q8x02027x3.mp.	0	Advanced

825	recozil.mp.	0	Advanced
826	reducel.mp.	2	Advanced
827	regulip.mp.	0	Advanced
828	synbrozil.mp.	0	Advanced
829	trialmin.mp.	0	Advanced
830	triglizil.mp.	0	Advanced
831	uragem.mp.	0	Advanced
832	zilop.mp.	0	Advanced
833	or/1-832	580753	Advanced
834	exp aneurysm, ruptured/	20770	Advanced
835	exp aortic aneurysm/	64253	Advanced
836	exp aortic dissection/	22532	Advanced
837	aneurysm?.mp.	159313	Advanced
838	aortic rupture*.mp.	11510	Advanced
839	loeys-dietz.mp.	738	Advanced
840	aortic dissection?.mp.	26307	Advanced
841	aorta dissection?.mp.	190	Advanced
842	aortic dilatation?.mp.	1256	Advanced
843	aortic ectasia?.mp.	239	Advanced
844	exp Aorta/su [Surgery]	27438	Advanced
845	((aorta or aortic) and (surgery or surgical)).mp.	151487	Advanced
846	(Bentall adj2 (repair or replacement or surgery)).mp.	123	Advanced
847	(David adj2 (repair or surgery)).mp.	19	Advanced
848	(aortic root adj2 (repair or replacement)).mp.	1893	Advanced
849	(Yacoub adj2 (repair or surgery)).mp.	0	Advanced
850	(hemiyacoub adj2 (repair or surgery)).mp.	0	Advanced

851	frozen elephant trunk surgery.mp.	12	Advanced
852	(hemiarch adj2 (repair or replacement)).mp.	352	Advanced
853	Marfan Syndrome/	6935	Advanced
854	(marfan* adj2 syndrome*).mp.	8483	Advanced
855	aortic h?ematoma.mp.	97	Advanced
856	aortic ulcer*.mp.	497	Advanced
857	tevar.mp.	2632	Advanced
858	evar.mp.	5144	Advanced
859	aortic endoprosthesis.mp.	58	Advanced
860	(aortic adj2 replacement).mp.	31156	Advanced
861	aortic growth.mp.	259	Advanced
862	or/834-861	272138	Advanced
863	833 and 862	3337	Advanced
864	animals/ not (animals/ and humans/)	5285392	Advanced
865	863 not 864	2920	Advanced
866	limit 865 to (english or french or spanish)	2716	Advanced
867	remove duplicates from 866	2715	Advanced

Embase <1974 to 2025 March 21>

#	Searches	Results	Type
1	exp antilipemic agent/	406142	Advanced
2	exp cholesterol/	409536	Advanced
3	exp hyperlipidemia/	215963	Advanced
4	antihyperlipemic*.tw,kf.	46	Advanced
5	Antilip?emic*.tw,kf.	537	Advanced
6	(hypolipidemic adj2 (agent? or drug?)).tw,kf.	2329	Advanced

7	(anticholesteremic adj2 (agent? or drug?)).tw,kf.	69	Advanced
8	(antihypertriglyceridic adj2 (agent? or drug?)).tw,kf.	0	Advanced
9	(antilip?emia adj2 (agent? or drug?)).tw,kf.	0	Advanced
10	(antilipidemic adj2 (agent? or drug?)).tw,kf.	155	Advanced
11	(hypolip?emic adj2 (agent? or drug?)).tw,kf.	241	Advanced
12	(hypotriglyceridic adj2 (agent? or drug?)).tw,kf.	0	Advanced
13	(lipid depressing adj2 (agent? or drug?)).tw,kf.	0	Advanced
14	(lipid lowering adj2 (agent? or drug?)).tw,kf.	10165	Advanced
15	Anticholesteremic?.tw,kf.	88	Advanced
16	(cholesterol adj2 inhibitor?).tw,kf.	2249	Advanced
17	(hypocholester?emic adj2 (agent? or drug?)).tw,kf.	27	Advanced
18	(Hypocholesterol?emic adj2 (agent? or drug?)).tw,kf.	597	Advanced
19	(antihypercholesteremic adj2 (agent? or drug?)).tw,kf.	2	Advanced
20	hmg coa reductase inhibitor?.tw,kf.	6837	Advanced
21	hmg coenzyme a reductase inhibitor?.tw,kf.	54	Advanced
22	hydroxymethylglutaryl coa reductase inhibitor?.tw,kf.	835	Advanced
23	hydroxymethylglutaryl-coa inhibitor?.tw,kf.	6	Advanced
24	hydroxymethylglutaryl-coenzyme a inhibitor?.tw,kf.	12	Advanced
25	statin?.tw,kf.	95596	Advanced
26	pcsk9 inhibitor?.tw,kf.	3797	Advanced
27	fatty acid synthesis inhibitor?.tw,kf.	65	Advanced
28	lipocaic?.tw,kf.	6	Advanced
29	(lipotropic adj2 (agent? or drug? or factor?)).tw,kf.	58	Advanced
30	Cholesterol.tw,kf.	395326	Advanced
31	Epicholesterol.tw,kf.	80	Advanced
32	Hyperlip?emia?.tw,kf.	2606	Advanced

33	Hyperlipid?emia?.tw,kf.	62928	Advanced
34	Lipemia?.tw,kf.	2735	Advanced
35	Lipid?emia?.tw,kf.	995	Advanced
36	Hypercholester?emia?.tw,kf.	760	Advanced
37	Hypercholesterol?emia?.tw,kf.	54935	Advanced
38	Hypercholesterin?emia.tw,kf.	304	Advanced
39	Hypertriglycerid?emia?.tw,kf.	24784	Advanced
40	Cholesteremia.tw,kf.	30	Advanced
41	Cholesterinemia.tw,kf.	7	Advanced
42	Cholesterolemia.tw,kf.	1543	Advanced
43	Atorvastatin.tw,kf.	18995	Advanced
44	a 2581175.tw,kf.	0	Advanced
45	a2581175.tw,kf.	0	Advanced
46	amicor.tw,kf.	0	Advanced
47	antorcin.tw,kf.	0	Advanced
48	apo−atorva.tw,kf.	0	Advanced
49	arkas.tw,kf.	5	Advanced
50	artas.tw,kf.	74	Advanced
51	ascord.tw,kf.	0	Advanced
52	astator.tw,kf.	0	Advanced
53	atacor.tw,kf.	10	Advanced
54	atofast.tw,kf.	1	Advanced
55	ator.tw,kf.	335	Advanced
56	ator−chol.tw,kf.	0	Advanced
57	atorab.tw,kf.	0	Advanced
58	atorbir.tw,kf.	0	Advanced

59	atorcor.tw,kf.	1	Advanced
60	atoridor.tw,kf.	0	Advanced
61	atorin.tw,kf.	5	Advanced
62	atoris.tw,kf.	23	Advanced
63	atorlip.tw,kf.	12	Advanced
64	atormax.tw,kf.	0	Advanced
65	atorstad.tw,kf.	0	Advanced
66	atorstan.tw,kf.	0	Advanced
67	atorstat.tw,kf.	1	Advanced
68	atorvadivid.tw,kf.	0	Advanced
69	atorvagen.tw,kf.	0	Advanced
70	atorval.tw,kf.	0	Advanced
71	atorvalan.tw,kf.	1	Advanced
72	atorvalet.tw,kf.	0	Advanced
73	atorvaliq.tw,kf.	0	Advanced
74	atorvanox.tw,kf.	0	Advanced
75	atorvasterol.tw,kf.	2	Advanced
76	atorvastine.tw,kf.	0	Advanced
77	atorvox.tw,kf.	8	Advanced
78	atorzem.tw,kf.	0	Advanced
79	atostat.tw,kf.	1	Advanced
80	atostin.tw,kf.	0	Advanced
81	atovans.tw,kf.	0	Advanced
82	atovarol.tw,kf.	0	Advanced
83	atractin.tw,kf.	1	Advanced
84	atraven.tw,kf.	0	Advanced

85	atrocell.tw,kf.	0	Advanced
86	atrost.tw,kf.	0	Advanced
87	atrox.tw,kf.	973	Advanced
88	calipra.tw,kf.	0	Advanced
89	card−ok.tw,kf.	0	Advanced
90	cardiostyl.tw,kf.	0	Advanced
91	cardyl.tw,kf.	7	Advanced
92	ci 981.tw,kf.	283	Advanced
93	ci981.tw,kf.	4	Advanced
94	decholest.tw,kf.	0	Advanced
95	delipost.tw,kf.	0	Advanced
96	gletor.tw,kf.	0	Advanced
97	glustar.tw,kf.	0	Advanced
98	lambrinex.tw,kf.	0	Advanced
99	larus.tw,kf.	1236	Advanced
100	latrovin.tw,kf.	0	Advanced
101	lipibec.tw,kf.	0	Advanced
102	lipigan.tw,kf.	0	Advanced
103	lipimed.tw,kf.	0	Advanced
104	lipistad.tw,kf.	0	Advanced
105	lipitor.tw,kf.	2233	Advanced
106	lipovast.tw,kf.	1	Advanced
107	liprimar.tw,kf.	17	Advanced
108	liptin.tw,kf.	14	Advanced
109	liptonorm.tw,kf.	3	Advanced
110	lorvaten.tw,kf.	0	Advanced

111	lowlipen.tw,kf.	0	Advanced
112	nelibat.tw,kf.	0	Advanced
113	obradon.tw,kf.	0	Advanced
114	omegastatin.tw,kf.	0	Advanced
115	orbeos.tw,kf.	0	Advanced
116	orvasta.tw,kf.	0	Advanced
117	pd 13429838a.tw,kf.	0	Advanced
118	pd13429838a.tw,kf.	0	Advanced
119	prevencor.tw,kf.	5	Advanced
120	provicard.tw,kf.	0	Advanced
121	rafitin.tw,kf.	0	Advanced
122	rotacor.tw,kf.	1	Advanced
123	rotova.tw,kf.	0	Advanced
124	"s 05153".tw,kf.	0	Advanced
125	s05153.tw,kf.	0	Advanced
126	sortis.tw,kf.	213	Advanced
127	statorva.tw,kf.	0	Advanced
128	storvas.tw,kf.	9	Advanced
129	tahor.tw,kf.	66	Advanced
130	thervan.tw,kf.	0	Advanced
131	torvacard.tw,kf.	8	Advanced
132	torvachol.tw,kf.	0	Advanced
133	torvacol.tw,kf.	0	Advanced
134	torvalipin.tw,kf.	0	Advanced
135	torvaplus.tw,kf.	0	Advanced
136	torvarin.tw,kf.	0	Advanced

137	torvas.tw,kf.	0	Advanced
138	torvast.tw,kf.	20	Advanced
139	torvastin.tw,kf.	0	Advanced
140	torvatec.tw,kf.	0	Advanced
141	torvazin.tw,kf.	0	Advanced
142	totalip.tw,kf.	4	Advanced
143	tulip.tw,kf.	1172	Advanced
144	vastat.tw,kf.	3	Advanced
145	vastatin.tw,kf.	92	Advanced
146	vastazor.tw,kf.	0	Advanced
147	voredanin.tw,kf.	0	Advanced
148	xarator.tw,kf.	3	Advanced
149	xolister−all.tw,kf.	0	Advanced
150	ym 548.tw,kf.	6	Advanced
151	ym548.tw,kf.	7	Advanced
152	zarastin.tw,kf.	0	Advanced
153	zarator.tw,kf.	13	Advanced
154	coupet.tw,kf.	0	Advanced
155	crestor.tw,kf.	1040	Advanced
156	epri.tw,kf.	304	Advanced
157	ezallor.tw,kf.	10	Advanced
158	"hgp 0816".tw,kf.	0	Advanced
159	hgp0816.tw,kf.	0	Advanced
160	mertenil.tw,kf.	4	Advanced
161	provisacor.tw,kf.	1	Advanced
162	ropitor.tw,kf.	4	Advanced

163	rostat.tw,kf.	0	Advanced
164	rosudia.tw,kf.	0	Advanced
165	rosumop.tw,kf.	0	Advanced
166	rosuvador.tw,kf.	0	Advanced
167	rosuvas.tw,kf.	13	Advanced
168	rosuvastatin.tw,kf.	8462	Advanced
169	rosuvastatina.tw,kf.	11	Advanced
170	rosuvastatine.tw,kf.	24	Advanced
171	roswera.tw,kf.	2	Advanced
172	roxera.tw,kf.	1	Advanced
173	rozuva−teva.tw,kf.	0	Advanced
174	s 4522.tw,kf.	7	Advanced
175	s4522.tw,kf.	0	Advanced
176	simestat.tw,kf.	2	Advanced
177	sorvasta.tw,kf.	0	Advanced
178	visacor.tw,kf.	24	Advanced
179	xeter.tw,kf.	1	Advanced
180	zahron.tw,kf.	0	Advanced
181	zaranta.tw,kf.	0	Advanced
182	zd 4522.tw,kf.	39	Advanced
183	zd4522.tw,kf.	5	Advanced
184	alipza.tw,kf.	4	Advanced
185	itavastatin.tw,kf.	9	Advanced
186	lippiza.tw,kf.	1	Advanced
187	livalo.tw,kf.	176	Advanced
188	livazo.tw,kf.	11	Advanced

189	nikita.tw,kf.	93	Advanced
190	nisvastatin.tw,kf.	9	Advanced
191	nk 104.tw,kf.	126	Advanced
192	nk104.tw,kf.	3	Advanced
193	nks 104.tw,kf.	6	Advanced
194	nks104.tw,kf.	0	Advanced
195	p 872441.tw,kf.	3	Advanced
196	p872441.tw,kf.	0	Advanced
197	pitava.tw,kf.	23	Advanced
198	pitavastatin.tw,kf.	2001	Advanced
199	redevant.tw,kf.	2	Advanced
200	ribar.tw,kf.	6	Advanced
201	trolise.tw,kf.	1	Advanced
202	vezepra.tw,kf.	1	Advanced
203	zypitamag.tw,kf.	12	Advanced
204	aplactin.tw,kf.	4	Advanced
205	astin.tw,kf.	66	Advanced
206	bristacol.tw,kf.	3	Advanced
207	cholespar.tw,kf.	0	Advanced
208	cs 514.tw,kf.	80	Advanced
209	cs514.tw,kf.	0	Advanced
210	dehypotin protect.tw,kf.	0	Advanced
211	elisor.tw,kf.	76	Advanced
212	epatostantin.tw,kf.	0	Advanced
213	eptastatin.tw,kf.	24	Advanced
214	eptastatine.tw,kf.	1	Advanced

215	kenstatin.tw,kf.	0	Advanced
216	lipemol.tw,kf.	4	Advanced
217	lipidal.tw,kf.	3	Advanced
218	liplat.tw,kf.	4	Advanced
219	lipostat.tw,kf.	121	Advanced
220	liprevil.tw,kf.	57	Advanced
221	maxudin.tw,kf.	0	Advanced
222	mevalotin.tw,kf.	114	Advanced
223	minuscol.tw,kf.	0	Advanced
224	novales.tw,kf.	5	Advanced
225	prareduct.tw,kf.	3	Advanced
226	prascolend.tw,kf.	0	Advanced
227	prastan.tw,kf.	3	Advanced
228	prasterol.tw,kf.	3	Advanced
229	prava.tw,kf.	48	Advanced
230	pravachol.tw,kf.	676	Advanced
231	pravacol.tw,kf.	13	Advanced
232	pravalam.tw,kf.	0	Advanced
233	pravalich.tw,kf.	0	Advanced
234	pravaselect.tw,kf.	6	Advanced
235	pravasin.tw,kf.	102	Advanced
236	pravasine.tw,kf.	14	Advanced
237	pravastatin.tw,kf.	6447	Advanced
238	pravastatine.tw,kf.	33	Advanced
239	pravator.tw,kf.	3	Advanced
240	pravyl.tw,kf.	0	Advanced

241	rms 431.tw,kf.	1	Advanced
242	sanaprav.tw,kf.	5	Advanced
243	selectin.tw,kf.	30135	Advanced
244	selektine.tw,kf.	34	Advanced
245	selipran.tw,kf.	43	Advanced
246	sq 31000.tw,kf.	14	Advanced
247	sq31000.tw,kf.	0	Advanced
248	stanidine.tw,kf.	0	Advanced
249	vasopran.tw,kf.	0	Advanced
250	vasten.tw,kf.	74	Advanced
251	versatab.tw,kf.	0	Advanced
252	xipral.tw,kf.	0	Advanced
253	alcosin.tw,kf.	0	Advanced
254	antichol.tw,kf.	3	Advanced
255	apo−simva.tw,kf.	0	Advanced
256	avastinee.tw,kf.	0	Advanced
257	belmalip.tw,kf.	0	Advanced
258	cholemed.tw,kf.	0	Advanced
259	cholestat.tw,kf.	3	Advanced
260	clinfar.tw,kf.	2	Advanced
261	colastatina.tw,kf.	0	Advanced
262	coledis.tw,kf.	0	Advanced
263	colemin.tw,kf.	3	Advanced
264	colemin forte.tw,kf.	0	Advanced
265	colestricon.tw,kf.	0	Advanced
266	corolin.tw,kf.	0	Advanced

267	corsim.tw,kf.	3	Advanced
268	covastin.tw,kf.	1	Advanced
269	denan.tw,kf.	60	Advanced
270	epistatin.tw,kf.	6	Advanced
271	esvat.tw,kf.	2	Advanced
272	eucor.tw,kf.	8	Advanced
273	flolipid.tw,kf.	10	Advanced
274	glipal.tw,kf.	0	Advanced
275	ifistatin.tw,kf.	0	Advanced
276	ipramid.tw,kf.	0	Advanced
277	jabastatina.tw,kf.	0	Advanced
278	kavelor.tw,kf.	0	Advanced
279	klonastin.tw,kf.	0	Advanced
280	kolestevan.tw,kf.	0	Advanced
281	kymazol.tw,kf.	0	Advanced
282	l 644128.tw,kf.	0	Advanced
283	l644128.tw,kf.	0	Advanced
284	labistatin.tw,kf.	0	Advanced
285	lepur.tw,kf.	4	Advanced
286	lip−down.tw,kf.	15	Advanced
287	lipaxin.tw,kf.	0	Advanced
288	lipcut.tw,kf.	0	Advanced
289	lipecor.tw,kf.	0	Advanced
290	lipex.tw,kf.	70	Advanced
291	lipinorm.tw,kf.	5	Advanced
292	lipomin.tw,kf.	3	Advanced

293	liponorm.tw,kf.	6	Advanced
294	liporex.tw,kf.	0	Advanced
295	lipovas.tw,kf.	29	Advanced
296	lipterra.tw,kf.	0	Advanced
297	lodales.tw,kf.	68	Advanced
298	lowcholid.tw,kf.	0	Advanced
299	medipo.tw,kf.	6	Advanced
300	medistatin.tw,kf.	0	Advanced
301	mersivas.tw,kf.	0	Advanced
302	"mk 0733".tw,kf.	5	Advanced
303	mk 733.tw,kf.	85	Advanced
304	mk0733.tw,kf.	0	Advanced
305	mk733.tw,kf.	1	Advanced
306	nezatin.tw,kf.	0	Advanced
307	nitastin.tw,kf.	0	Advanced
308	nivelipol.tw,kf.	0	Advanced
309	nor−vastina.tw,kf.	0	Advanced
310	normofat.tw,kf.	1	Advanced
311	nyzoc.tw,kf.	0	Advanced
312	omistat.tw,kf.	0	Advanced
313	orovas.tw,kf.	0	Advanced
314	pantok.tw,kf.	2	Advanced
315	placol.tw,kf.	6	Advanced
316	pravostin.tw,kf.	0	Advanced
317	priacin.tw,kf.	0	Advanced
318	protecta.tw,kf.	44	Advanced

319	ranzolont.tw,kf.	1	Advanced
320	raptor.tw,kf.	3238	Advanced
321	rechol.tw,kf.	0	Advanced
322	redusterol.tw,kf.	0	Advanced
323	rendapid.tw,kf.	0	Advanced
324	simar.tw,kf.	19	Advanced
325	simbado.tw,kf.	0	Advanced
326	simbatrix.tw,kf.	0	Advanced
327	simcard.tw,kf.	5	Advanced
328	simchol.tw,kf.	0	Advanced
329	simcovas.tw,kf.	0	Advanced
330	simorion.tw,kf.	0	Advanced
331	simovil.tw,kf.	6	Advanced
332	simratio.tw,kf.	0	Advanced
333	simtan.tw,kf.	0	Advanced
334	simtin.tw,kf.	2	Advanced
335	simva.tw,kf.	155	Advanced
336	simvabeta.tw,kf.	1	Advanced
337	simvacard.tw,kf.	13	Advanced
338	simvachol.tw,kf.	2	Advanced
339	simvacol.tw,kf.	2	Advanced
340	simvacor.tw,kf.	9	Advanced
341	simvador.tw,kf.	7	Advanced
342	simvadura.tw,kf.	1	Advanced
343	simvagen.tw,kf.	1	Advanced
344	simvahex.tw,kf.	1	Advanced

345	simvahexal.tw,kf.	10	Advanced
346	simvalid.tw,kf.	0	Advanced
347	simvalimit.tw,kf.	0	Advanced
348	simvalord.tw,kf.	0	Advanced
349	simvasim.tw,kf.	0	Advanced
350	simvastar.tw,kf.	3	Advanced
351	simvastatin.tw,kf.	17404	Advanced
352	simvastatina.tw,kf.	12	Advanced
353	simvastatine.tw,kf.	81	Advanced
354	simvasterol.tw,kf.	2	Advanced
355	simvata.tw,kf.	0	Advanced
356	simvatin.tw,kf.	3	Advanced
357	simvax.tw,kf.	3	Advanced
358	simvor.tw,kf.	17	Advanced
359	simvotin.tw,kf.	8	Advanced
360	sincol.tw,kf.	0	Advanced
361	sinpor.tw,kf.	0	Advanced
362	sintenal.tw,kf.	0	Advanced
363	sinvacor.tw,kf.	13	Advanced
364	sinvalip.tw,kf.	0	Advanced
365	sinvastatin.tw,kf.	44	Advanced
366	sinvinolin.tw,kf.	1	Advanced
367	sivastin.tw,kf.	5	Advanced
368	sivatin.tw,kf.	0	Advanced
369	starzoco.tw,kf.	0	Advanced
370	stasiva.tw,kf.	0	Advanced

371	statinum.tw,kf.	0	Advanced
372	stativer.tw,kf.	0	Advanced
373	synvinolin.tw,kf.	38	Advanced
374	torio.tw,kf.	4	Advanced
375	tremital.tw,kf.	0	Advanced
376	valemia.tw,kf.	0	Advanced
377	vasilip.tw,kf.	25	Advanced
378	vasotenal.tw,kf.	0	Advanced
379	vastan.tw,kf.	8	Advanced
380	vastatin.tw,kf.	92	Advanced
381	vastin.tw,kf.	18	Advanced
382	vazim.tw,kf.	0	Advanced
383	velastatin.tw,kf.	0	Advanced
384	velkastatin.tw,kf.	0	Advanced
385	velostatin.tw,kf.	0	Advanced
386	veristin.tw,kf.	0	Advanced
387	veritrat.tw,kf.	0	Advanced
388	vidastat.tw,kf.	0	Advanced
389	ximve.tw,kf.	0	Advanced
390	xipocol.tw,kf.	0	Advanced
391	zeplan.tw,kf.	0	Advanced
392	zimmex.tw,kf.	1	Advanced
393	zocor.tw,kf.	2030	Advanced
394	zocord.tw,kf.	26	Advanced
395	zorced.tw,kf.	1	Advanced
396	zosta.tw,kf.	4	Advanced

397	zovar.tw,kf.	0	Advanced
398	zovast.tw,kf.	0	Advanced
399	altocor.tw,kf.	46	Advanced
400	altoprev.tw,kf.	72	Advanced
401	artein.tw,kf.	5	Advanced
402	belvas.tw,kf.	0	Advanced
403	birotin.tw,kf.	0	Advanced
404	cholestra.tw,kf.	4	Advanced
405	cysin.tw,kf.	0	Advanced
406	ellanco.tw,kf.	0	Advanced
407	elstatin.tw,kf.	1	Advanced
408	l 654969.tw,kf.	19	Advanced
409	lipdip.tw,kf.	0	Advanced
410	lipivas.tw,kf.	5	Advanced
411	lofacol.tw,kf.	0	Advanced
412	lomar.tw,kf.	1	Advanced
413	lostatin.tw,kf.	5	Advanced
414	lovacel.tw,kf.	0	Advanced
415	lovacol.tw,kf.	5	Advanced
416	lovahexal.tw,kf.	0	Advanced
417	lovalip.tw,kf.	1	Advanced
418	lovalord.tw,kf.	0	Advanced
419	lovastan.tw,kf.	0	Advanced
420	lovastatin.tw,kf.	5751	Advanced
421	lovasterol.tw,kf.	1	Advanced
422	lovastin.tw,kf.	18	Advanced

423	lovatadin.tw,kf.	0	Advanced
424	lowachol.tw,kf.	0	Advanced
425	lozutin.tw,kf.	0	Advanced
426	medostatin.tw,kf.	4	Advanced
427	mevacor.tw,kf.	825	Advanced
428	meverstin.tw,kf.	0	Advanced
429	mevinacor.tw,kf.	100	Advanced
430	"mk 0803".tw,kf.	2	Advanced
431	mk 803.tw,kf.	32	Advanced
432	mk0803.tw,kf.	0	Advanced
433	mk803.tw,kf.	1	Advanced
434	monacolin K.tw,kf.	370	Advanced
435	monakolin k.tw,kf.	7	Advanced
436	msd 803.tw,kf.	1	Advanced
437	neolipid.tw,kf.	10	Advanced
438	nergadan.tw,kf.	6	Advanced
439	ovasta.tw,kf.	0	Advanced
440	rodatin.tw,kf.	0	Advanced
441	rovacor.tw,kf.	1	Advanced
442	taucor.tw,kf.	4	Advanced
443	almastatin.tw,kf.	1	Advanced
444	canef.tw,kf.	30	Advanced
445	cardiol.tw,kf.	2990	Advanced
446	cranoc.tw,kf.	35	Advanced
447	digardil.tw,kf.	0	Advanced
448	digaril.tw,kf.	0	Advanced

449	fluindostatin.tw,kf.	6	Advanced
450	fluvastatin.tw,kf.	3073	Advanced
451	fluvastatina.tw,kf.	0	Advanced
452	fluvastatine.tw,kf.	10	Advanced
453	fractal.tw,kf.	14482	Advanced
454	leposit prolib.tw,kf.	0	Advanced
455	lescol.tw,kf.	756	Advanced
456	leucol.tw,kf.	0	Advanced
457	lipaxan.tw,kf.	1	Advanced
458	lipaxin.tw,kf.	0	Advanced
459	liposit.tw,kf.	1	Advanced
460	lochol.tw,kf.	7	Advanced
461	locol.tw,kf.	53	Advanced
462	lymetel.tw,kf.	2	Advanced
463	primesin.tw,kf.	1	Advanced
464	primexin.tw,kf.	0	Advanced
465	sri 62320.tw,kf.	6	Advanced
466	sri62320.tw,kf.	0	Advanced
467	vaditon.tw,kf.	0	Advanced
468	vaditon prolib.tw,kf.	0	Advanced
469	vastin.tw,kf.	18	Advanced
470	xu 62 320.tw,kf.	9	Advanced
471	xu 62320.tw,kf.	26	Advanced
472	xu62320.tw,kf.	0	Advanced
473	anj 900.tw,kf.	0	Advanced
474	anj900.tw,kf.	0	Advanced

475	apophage.tw,kf.	0	Advanced
476	aron.tw,kf.	271	Advanced
477	benofomin.tw,kf.	0	Advanced
478	dabex.tw,kf.	7	Advanced
479	denkaform.tw,kf.	0	Advanced
480	deson.tw,kf.	1	Advanced
481	dextin.tw,kf.	2	Advanced
482	diabetase.tw,kf.	3	Advanced
483	diabetase s.tw,kf.	0	Advanced
484	diabetformin.tw,kf.	0	Advanced
485	diabetmin.tw,kf.	14	Advanced
486	diabetosan.tw,kf.	2	Advanced
487	diabex.tw,kf.	57	Advanced
488	diafat.tw,kf.	0	Advanced
489	diaformin.tw,kf.	24	Advanced
490	diaformina.tw,kf.	0	Advanced
491	diametin.tw,kf.	0	Advanced
492	diamin.tw,kf.	27	Advanced
493	dianben.tw,kf.	27	Advanced
494	diformin.tw,kf.	24	Advanced
495	dimefor.tw,kf.	10	Advanced
496	dimethylbiguanide.tw,kf.	154	Advanced
497	dimethyldiguanide.tw,kf.	11	Advanced
498	dmgg.tw,kf.	1	Advanced
499	dybis.tw,kf.	0	Advanced
500	"efb 0027".tw,kf.	0	Advanced

501	efb0027.tw,kf.	1	Advanced
502	eraphage.tw,kf.	0	Advanced
503	espa−formin.tw,kf.	0	Advanced
504	euform retard.tw,kf.	0	Advanced
505	fluamine.tw,kf.	0	Advanced
506	flumamine.tw,kf.	3	Advanced
507	fornidd.tw,kf.	0	Advanced
508	fortamet.tw,kf.	75	Advanced
509	glafornil.tw,kf.	4	Advanced
510	glibudon.tw,kf.	0	Advanced
511	glifage.tw,kf.	35	Advanced
512	gliguanid.tw,kf.	0	Advanced
513	glucaminol.tw,kf.	0	Advanced
514	glucofage.tw,kf.	3	Advanced
515	glucofago.tw,kf.	0	Advanced
516	glucoform.tw,kf.	0	Advanced
517	glucoformin.tw,kf.	8	Advanced
518	glucohexal.tw,kf.	5	Advanced
519	glucoless.tw,kf.	1	Advanced
520	glucomet.tw,kf.	10	Advanced
521	glucomin.tw,kf.	2	Advanced
522	glucomine.tw,kf.	0	Advanced
523	gluconil.tw,kf.	2	Advanced
524	glucophage.tw,kf.	1909	Advanced
525	glucostop.tw,kf.	1	Advanced
526	glucotika.tw,kf.	0	Advanced

527	gludepatic.tw,kf.	0	Advanced
528	glufor.tw,kf.	0	Advanced
529	gluformin.tw,kf.	9	Advanced
530	glukophage.tw,kf.	0	Advanced
531	glumeformin.tw,kf.	0	Advanced
532	glumet.tw,kf.	5	Advanced
533	glumetza.tw,kf.	92	Advanced
534	glupa.tw,kf.	2	Advanced
535	glustress.tw,kf.	0	Advanced
536	glyciphage.tw,kf.	25	Advanced
537	glycomet.tw,kf.	33	Advanced
538	glycon.tw,kf.	130	Advanced
539	glycoran.tw,kf.	9	Advanced
540	glyformin.tw,kf.	1	Advanced
541	glymet.tw,kf.	10	Advanced
542	haurymellin.tw,kf.	3	Advanced
543	hipoglucin.tw,kf.	0	Advanced
544	i−max.tw,kf.	308	Advanced
545	islotin.tw,kf.	0	Advanced
546	jesacrin.tw,kf.	0	Advanced
547	juformin.tw,kf.	1	Advanced
548	la 6023.tw,kf.	0	Advanced
549	la6023.tw,kf.	0	Advanced
550	lyomet.tw,kf.	0	Advanced
551	maformin.tw,kf.	0	Advanced
552	meglucon.tw,kf.	7	Advanced

553	meguan.tw,kf.	7	Advanced
554	melbin.tw,kf.	12	Advanced
555	melformin.tw,kf.	7	Advanced
556	mellittin.tw,kf.	1	Advanced
557	merckformin.tw,kf.	2	Advanced
558	mescorit.tw,kf.	8	Advanced
559	metaformin.tw,kf.	10	Advanced
560	metfogamma.tw,kf.	8	Advanced
561	metfoliquid geriasan.tw,kf.	2	Advanced
562	metforal.tw,kf.	26	Advanced
563	metformax.tw,kf.	24	Advanced
564	metformin.tw,kf.	53473	Advanced
565	metformina.tw,kf.	72	Advanced
566	metformine.tw,kf.	292	Advanced
567	methformin.tw,kf.	8	Advanced
568	metiguanide.tw,kf.	5	Advanced
569	metomin.tw,kf.	3	Advanced
570	metphormin.tw,kf.	3	Advanced
571	miformin.tw,kf.	0	Advanced
572	dimethylguanylguanide.tw,kf.	0	Advanced
573	dimethylguanylguanidine.tw,kf.	3	Advanced
574	dimethyldiguanide.tw,kf.	11	Advanced
575	dimethyl biguanidine.tw,kf.	0	Advanced
576	dimethylbiguanide.tw,kf.	154	Advanced
577	dimethylbiguanidine.tw,kf.	10	Advanced
578	dimethyldiguanide.tw,kf.	11	Advanced

579	dimethylguanylguanidine.tw,kf.	3	Advanced
580	neoform.tw,kf.	10	Advanced
581	newmet.tw,kf.	1	Advanced
582	nndg.tw,kf.	2	Advanced
583	reglus−500.tw,kf.	0	Advanced
584	riomet.tw,kf.	61	Advanced
585	risidon.tw,kf.	4	Advanced
586	rudimet.tw,kf.	0	Advanced
587	siamformet.tw,kf.	0	Advanced
588	siofor.tw,kf.	46	Advanced
589	thiabet.tw,kf.	0	Advanced
590	vimetrol.tw,kf.	0	Advanced
591	walaphage.tw,kf.	0	Advanced
592	absorcol.tw,kf.	2	Advanced
593	adezop.tw,kf.	0	Advanced
594	azibe.tw,kf.	0	Advanced
595	carditimib.tw,kf.	0	Advanced
596	cexado.tw,kf.	0	Advanced
597	cildar.tw,kf.	0	Advanced
598	coltowan.tw,kf.	0	Advanced
599	corintus.tw,kf.	0	Advanced
600	delipid.tw,kf.	9	Advanced
601	egitim.tw,kf.	25	Advanced
602	elanix.tw,kf.	0	Advanced
603	erezel.tw,kf.	0	Advanced
604	esetin.tw,kf.	0	Advanced

605	etibax.tw,kf.	0	Advanced
606	ezdivule.tw,kf.	0	Advanced
607	ezedoc.tw,kf.	4	Advanced
608	ezegelan.tw,kf.	0	Advanced
609	ezegros.tw,kf.	0	Advanced
610	ezehron.tw,kf.	0	Advanced
611	ezelip.tw,kf.	0	Advanced
612	ezemed.tw,kf.	0	Advanced
613	ezen.tw,kf.	0	Advanced
614	ezetad.tw,kf.	0	Advanced
615	ezetebmib.tw,kf.	0	Advanced
616	ezetib.tw,kf.	2	Advanced
617	ezetiben.tw,kf.	0	Advanced
618	ezetimib.tw,kf.	75	Advanced
619	ezetimibe.tw,kf.	7819	Advanced
620	ezetrol.tw,kf.	265	Advanced
621	ezetymib.tw,kf.	0	Advanced
622	ezgal.tw,kf.	0	Advanced
623	ezicor.tw,kf.	0	Advanced
624	ezoleta.tw,kf.	0	Advanced
625	hgp 1404.tw,kf.	0	Advanced
626	hgp1404.tw,kf.	0	Advanced
627	intestat.tw,kf.	0	Advanced
628	kobey.tw,kf.	0	Advanced
629	lipegis.tw,kf.	0	Advanced
630	lipobon.tw,kf.	0	Advanced

631	mibecol.tw,kf.	0	Advanced
632	mibezet.tw,kf.	0	Advanced
633	noveze.tw,kf.	0	Advanced
634	noxetib.tw,kf.	0	Advanced
635	olufsent.tw,kf.	0	Advanced
636	quver.tw,kf.	2	Advanced
637	sch 58235.tw,kf.	64	Advanced
638	sch58235.tw,kf.	5	Advanced
639	strami.tw,kf.	1	Advanced
640	symezet.tw,kf.	0	Advanced
641	tezzimi.tw,kf.	0	Advanced
642	tritilz.tw,kf.	0	Advanced
643	trizibe.tw,kf.	0	Advanced
644	viemm.tw,kf.	0	Advanced
645	vyeve.tw,kf.	0	Advanced
646	ylerse.tw,kf.	0	Advanced
647	zertya.tw,kf.	0	Advanced
648	zetaxyl.tw,kf.	0	Advanced
649	zetia.tw,kf.	409	Advanced
650	zetiraf.tw,kf.	0	Advanced
651	zetratak.tw,kf.	0	Advanced
652	zetratak−s.tw,kf.	0	Advanced
653	zient.tw,kf.	9	Advanced
654	alirocumab.tw,kf.	1704	Advanced
655	praluent.tw,kf.	190	Advanced
656	regn 727.tw,kf.	130	Advanced

657	regn727.tw,kf.	48	Advanced
658	sar 236553.tw,kf.	107	Advanced
659	sar236553.tw,kf.	43	Advanced
660	evolucumab.tw,kf.	27	Advanced
661	amg 145.tw,kf.	209	Advanced
662	amg145.tw,kf.	16	Advanced
663	repatha.tw,kf.	219	Advanced
664	"sal 003".tw,kf.	8	Advanced
665	sal003.tw,kf.	19	Advanced
666	aln 60212.tw,kf.	4	Advanced
667	aln pcssc.tw,kf.	15	Advanced
668	aln60212.tw,kf.	0	Advanced
669	inclisiran.tw,kf.	736	Advanced
670	kjx 839.tw,kf.	1	Advanced
671	kjx839.tw,kf.	0	Advanced
672	leqvio.tw,kf.	72	Advanced
673	antara.tw,kf.	58	Advanced
674	apo−feno−micro.tw,kf.	0	Advanced
675	apteor.tw,kf.	0	Advanced
676	aterolis.tw,kf.	0	Advanced
677	biofibrat.tw,kf.	0	Advanced
678	bisterol sr.tw,kf.	0	Advanced
679	catalip.tw,kf.	0	Advanced
680	climage.tw,kf.	0	Advanced
681	controlip.tw,kf.	2	Advanced
682	durafenat.tw,kf.	0	Advanced

683	elipsia.tw,kf.	0	Advanced
684	evothyl.tw,kf.	0	Advanced
685	febira.tw,kf.	0	Advanced
686	fegenor.tw,kf.	3	Advanced
687	felosma.tw,kf.	0	Advanced
688	fenardin.tw,kf.	1	Advanced
689	fenobrat.tw,kf.	1	Advanced
690	fenobrate.tw,kf.	4	Advanced
691	fenochol.tw,kf.	0	Advanced
692	fenofanton.tw,kf.	0	Advanced
693	fenofibrate.tw,kf.	6082	Advanced
694	fenofix.tw,kf.	3	Advanced
695	fenogal.tw,kf.	22	Advanced
696	fenoglide.tw,kf.	28	Advanced
697	fenolip.tw,kf.	1	Advanced
698	fenomax.tw,kf.	2	Advanced
699	fenosup lidose.tw,kf.	0	Advanced
700	fenoswiss.tw,kf.	0	Advanced
701	fenox.tw,kf.	14	Advanced
702	fibrafen.tw,kf.	0	Advanced
703	fulcro.tw,kf.	1	Advanced
704	fulcrosupra.tw,kf.	0	Advanced
705	grofibrat.tw,kf.	1	Advanced
706	"grs 001".tw,kf.	4	Advanced
707	grs001.tw,kf.	0	Advanced
708	hyperchol.tw,kf.	9	Advanced

709	katalip.tw,kf.	3	Advanced
710	lexemin.tw,kf.	1	Advanced
711	lifenoz−u.tw,kf.	0	Advanced
712	lipanthyl.tw,kf.	479	Advanced
713	lipanthyl.tw,kf.	479	Advanced
714	lipanthylnano.tw,kf.	3	Advanced
715	lipantil.tw,kf.	38	Advanced
716	lipantyl.tw,kf.	17	Advanced
717	liparison.tw,kf.	6	Advanced
718	lipcor.tw,kf.	2	Advanced
719	liperial.tw,kf.	0	Advanced
720	lipidax.tw,kf.	0	Advanced
721	lipidil.tw,kf.	91	Advanced
722	lipilfen.tw,kf.	2	Advanced
723	lipilo.tw,kf.	5	Advanced
724	lipirex.tw,kf.	26	Advanced
725	lipoclar.tw,kf.	4	Advanced
726	lipofen.tw,kf.	30	Advanced
727	lipofene.tw,kf.	0	Advanced
728	lipofib.tw,kf.	0	Advanced
729	lipolin.tw,kf.	0	Advanced
730	liposit.tw,kf.	1	Advanced
731	lipsin.tw,kf.	14	Advanced
732	livesan ge.tw,kf.	0	Advanced
733	lofibra.tw,kf.	29	Advanced
734	nopid 200.tw,kf.	0	Advanced

735	normalip.tw,kf.	46	Advanced
736	nubrex.tw,kf.	0	Advanced
737	procetofen.tw,kf.	44	Advanced
738	procetofenate.tw,kf.	0	Advanced
739	procetofene.tw,kf.	31	Advanced
740	procetofen.tw,kf.	44	Advanced
741	qualipantyl.tw,kf.	0	Advanced
742	rapidil.tw,kf.	0	Advanced
743	redose 200.tw,kf.	0	Advanced
744	rorit.tw,kf.	2	Advanced
745	rp 1824.tw,kf.	0	Advanced
746	rp1824.tw,kf.	0	Advanced
747	secalip.tw,kf.	22	Advanced
748	sigurtil.tw,kf.	2	Advanced
749	supralip.tw,kf.	9	Advanced
750	suprelip.tw,kf.	2	Advanced
751	trichol.tw,kf.	6	Advanced
752	tricor.tw,kf.	393	Advanced
753	triglide.tw,kf.	108	Advanced
754	trolip.tw,kf.	0	Advanced
755	xafenor.tw,kf.	0	Advanced
756	zerlubron.tw,kf.	0	Advanced
757	ziglip.tw,kf.	0	Advanced
758	zigotrig.tw,kf.	0	Advanced
759	zumafib.tw,kf.	0	Advanced
760	ausgem.tw,kf.	3	Advanced

761	bolutol.tw,kf.	2	Advanced
762	brozil.tw,kf.	2	Advanced
763	chlorestrol.tw,kf.	0	Advanced
764	cholespid.tw,kf.	0	Advanced
765	ci 719.tw,kf.	361	Advanced
766	ci719.tw,kf.	7	Advanced
767	clearol.tw,kf.	0	Advanced
768	decrelip.tw,kf.	2	Advanced
769	detrichol.tw,kf.	0	Advanced
770	elmogan.tw,kf.	1	Advanced
771	fetinor.tw,kf.	0	Advanced
772	fibralip.tw,kf.	0	Advanced
773	fibrocit.tw,kf.	0	Advanced
774	gedum.tw,kf.	0	Advanced
775	gemfi.tw,kf.	4	Advanced
776	gemfibril.tw,kf.	0	Advanced
777	gemfibromax.tw,kf.	0	Advanced
778	gemfibrozil.tw,kf.	2692	Advanced
779	gemfibrozil.tw,kf.	2692	Advanced
780	gemfibrozilo.tw,kf.	1	Advanced
781	gemhexal.tw,kf.	3	Advanced
782	gemizol.tw,kf.	0	Advanced
783	gemlipid.tw,kf.	1	Advanced
784	gemnpid.tw,kf.	0	Advanced
785	gemzil.tw,kf.	2	Advanced
786	genfibrozil.tw,kf.	7	Advanced

787	gevilon.tw,kf.	92	Advanced
788	gevilon uno.tw,kf.	0	Advanced
789	gozid.tw,kf.	0	Advanced
790	grifogemzilo.tw,kf.	0	Advanced
791	hidil.tw,kf.	2	Advanced
792	hipolixan.tw,kf.	0	Advanced
793	ipolipid.tw,kf.	0	Advanced
794	jezil.tw,kf.	5	Advanced
795	lanaterom.tw,kf.	0	Advanced
796	lifibron.tw,kf.	0	Advanced
797	lipazil.tw,kf.	2	Advanced
798	lipidys.tw,kf.	0	Advanced
799	lipigem.tw,kf.	0	Advanced
800	lipira.tw,kf.	0	Advanced
801	lipison.tw,kf.	0	Advanced
802	lipistorol.tw,kf.	0	Advanced
803	lipizyl.tw,kf.	0	Advanced
804	lipofor.tw,kf.	0	Advanced
805	lipolo.tw,kf.	0	Advanced
806	lipostorol.tw,kf.	0	Advanced
807	lipox gemfi.tw,kf.	0	Advanced
808	lipozid.tw,kf.	7	Advanced
809	lipozil.tw,kf.	0	Advanced
810	lipur.tw,kf.	56	Advanced
811	litarek.tw,kf.	2	Advanced
812	lopid.tw,kf.	512	Advanced

813	lowin.tw,kf.	77	Advanced
814	low−lip.tw,kf.	27	Advanced
815	manobrozil.tw,kf.	0	Advanced
816	mariston.tw,kf.	0	Advanced
817	mersikol.tw,kf.	0	Advanced
818	normolip.tw,kf.	1	Advanced
819	panazil.tw,kf.	0	Advanced
820	pilder.tw,kf.	3	Advanced
821	polyxit.tw,kf.	0	Advanced
822	progemzal.tw,kf.	0	Advanced
823	q8x02027x3.tw,kf.	0	Advanced
824	recozil.tw,kf.	0	Advanced
825	reducel.tw,kf.	1	Advanced
826	regulip.tw,kf.	0	Advanced
827	synbrozil.tw,kf.	0	Advanced
828	trialmin.tw,kf.	8	Advanced
829	triglizil.tw,kf.	0	Advanced
830	uragem.tw,kf.	0	Advanced
831	zilop.tw,kf.	0	Advanced
832	or/1-831	1084182	Advanced
833	aneurysm rupture/	18355	Advanced
834	exp aortic aneurysm/	76604	Advanced
835	exp aortic dissection/	32456	Advanced
836	aneurysm?.tw,kf.	175063	Advanced
837	aortic rupture*.tw,kf.	3618	Advanced
838	loeys-dietz.tw,kf.	1268	Advanced

839	aortic dissection?.tw,kf.	27015	Advanced
840	aorta dissection?.tw,kf.	439	Advanced
841	aortic dilatation?.tw,kf.	2429	Advanced
842	aortic ectasia?.tw,kf.	329	Advanced
843	exp aorta/su [Surgery]	6751	Advanced
844	exp aortic surgery/	64598	Advanced
845	((aorta or aortic) and (surgery or surgical)).tw,kf.	132719	Advanced
846	(Bentall adj2 (repair or replacement or surgery)).tw,kf.	295	Advanced
847	(David adj2 (repair or surgery)).tw,kf.	38	Advanced
848	(aortic root adj2 (repair or replacement)).tw,kf.	2943	Advanced
849	(Yacoub adj2 (repair or surgery)).tw,kf.	8	Advanced
850	(hemiyacoub adj2 (repair or surgery)).tw,kf.	0	Advanced
851	frozen elephant trunk surgery.tw,kf.	33	Advanced
852	(hemiarch adj2 (repair or replacement)).tw,kf.	548	Advanced
853	Marfan Syndrome/	12489	Advanced
854	(marfan* adj2 syndrome*).tw,kf.	9851	Advanced
855	aortic h?ematoma.tw,kf.	182	Advanced
856	aortic ulcer*.tw,kf.	907	Advanced
857	tevar.tw,kf.	5176	Advanced
858	evar.tw,kf.	9579	Advanced
859	aortic endoprosthesis.tw,kf.	131	Advanced
860	(aortic adj2 replacement).tw,kf.	46569	Advanced
861	aortic growth.tw,kf.	482	Advanced
862	or/833-861	353595	Advanced
863	832 and 862	12213	Advanced

864	limit 863 to (conference abstract or conference paper or "conference review" or conference proceeding)	2883	Advanced
865	863 not 864	9330	Advanced
866	(exp animals/ or exp animal experimentation/ or nonhuman/) not ((exp animals/ or exp animal experimentation/ or nonhuman/) and exp human/)	7712329	Advanced
867	865 not 866	8804	Advanced
868	limit 867 to (english or french or spanish)	8526	Advanced

EBM Reviews - Cochrane Central Register of Controlled Trials

#	Searches	Results	Type
1	exp Hypolipidemic Agents/	15300	Advanced
2	exp Cholesterol/	12794	Advanced
3	exp Hyperlipidemias/	7966	Advanced
4	antihyperlipemic*.mp.	5	Advanced
5	Antilip?emic*.mp.	398	Advanced
6	(hypolipidemic adj2 (agent? or drug?)).mp.	2084	Advanced
7	(anticholesteremic adj2 (agent? or drug?)).mp.	3282	Advanced
8	(antihypertriglyceridic adj2 (agent? or drug?)).mp.	0	Advanced
9	(antilip?emia adj2 (agent? or drug?)).mp.	0	Advanced
10	(antilipidemic adj2 (agent? or drug?)).mp.	13	Advanced
11	(hypolip?emic adj2 (agent? or drug?)).mp.	34	Advanced
12	(hypotriglyceridic adj2 (agent? or drug?)).mp.	0	Advanced
13	(lipid depressing adj2 (agent? or drug?)).mp.	0	Advanced
14	(lipid lowering adj2 (agent? or drug?)).mp.	1175	Advanced
15	Anticholesteremic?.mp.	3282	Advanced
16	(cholesterol adj2 inhibitor?).mp.	187	Advanced

17	(hypocholester?emic adj2 (agent? or drug?)).mp.	1	Advanced
18	(Hypocholesterol?emic adj2 (agent? or drug?)).mp.	107	Advanced
19	(antihypercholesteremic adj2 (agent? or drug?)).mp.	0	Advanced
20	hmg coa reductase inhibitor?.mp.	879	Advanced
21	hmg coenzyme a reductase inhibitor?.mp.	11	Advanced
22	hydroxymethylglutaryl coa reductase inhibitor?.mp.	4973	Advanced
23	hydroxymethylglutaryl-coa inhibitor?.mp.	0	Advanced
24	hydroxymethylglutaryl-coenzyme a inhibitor?.mp.	1	Advanced
25	statin?.mp.	12078	Advanced
26	pcsk9 inhibitor?.mp.	550	Advanced
27	fatty acid synthesis inhibitor?.mp.	1	Advanced
28	lipocaic?.mp.	1	Advanced
29	(lipotropic adj2 (agent? or drug? or factor?)).mp.	22	Advanced
30	Cholesterol.mp.	44704	Advanced
31	Epicholesterol.mp.	0	Advanced
32	Hyperlip?emia?.mp.	342	Advanced
33	Hyperlipid?emia?.mp.	7314	Advanced
34	Lipemia?.mp.	470	Advanced
35	Lipid?emia?.mp.	160	Advanced
36	Hypercholester?emia?.mp.	83	Advanced
37	Hypercholesterol?emia?.mp.	8664	Advanced
38	Hypercholesterin?emia.mp.	6	Advanced
39	Hypertriglycerid?emia?.mp.	2634	Advanced
40	Cholesteremia.mp.	3	Advanced
41	Cholesterinemia.mp.	0	Advanced
42	Cholesterolemia.mp.	108	Advanced

43	Atorvastatin.mp.	6158	Advanced
44	a 2581175.mp.	0	Advanced
45	a2581175.mp.	0	Advanced
46	amicor.mp.	0	Advanced
47	antorcin.mp.	0	Advanced
48	apo−atorva.mp.	0	Advanced
49	arkas.mp.	0	Advanced
50	artas.mp.	16	Advanced
51	ascord.mp.	0	Advanced
52	astator.mp.	0	Advanced
53	atacor.mp.	0	Advanced
54	atofast.mp.	0	Advanced
55	ator.mp.	21	Advanced
56	ator−chol.mp.	0	Advanced
57	atorab.mp.	0	Advanced
58	atorbir.mp.	0	Advanced
59	atorcor.mp.	0	Advanced
60	atoridor.mp.	0	Advanced
61	atorin.mp.	0	Advanced
62	atoris.mp.	2	Advanced
63	atorlip.mp.	2	Advanced
64	atormax.mp.	0	Advanced
65	atorstad.mp.	0	Advanced
66	atorstan.mp.	0	Advanced
67	atorstat.mp.	0	Advanced
68	atorvadivid.mp.	0	Advanced

69	atorvagen.mp.	0	Advanced
70	atorval.mp.	0	Advanced
71	atorvalan.mp.	0	Advanced
72	atorvalet.mp.	0	Advanced
73	atorvaliq.mp.	0	Advanced
74	atorvanox.mp.	0	Advanced
75	atorvasterol.mp.	0	Advanced
76	atorvastine.mp.	0	Advanced
77	atorvox.mp.	0	Advanced
78	atorzem.mp.	0	Advanced
79	atostat.mp.	0	Advanced
80	atostin.mp.	0	Advanced
81	atovans.mp.	0	Advanced
82	atovarol.mp.	0	Advanced
83	atractin.mp.	0	Advanced
84	atraven.mp.	0	Advanced
85	atrocell.mp.	0	Advanced
86	atrost.mp.	0	Advanced
87	atrox.mp.	20	Advanced
88	calipra.mp.	0	Advanced
89	card−ok.mp.	0	Advanced
90	cardiostyl.mp.	0	Advanced
91	cardyl.mp.	5	Advanced
92	ci 981.mp.	40	Advanced
93	ci981.mp.	0	Advanced
94	decholest.mp.	0	Advanced

95	delipost.mp.	0	Advanced
96	gletor.mp.	0	Advanced
97	glustar.mp.	0	Advanced
98	lambrinex.mp.	0	Advanced
99	larus.mp.	1	Advanced
100	latrovin.mp.	0	Advanced
101	lipibec.mp.	0	Advanced
102	lipigan.mp.	0	Advanced
103	lipimed.mp.	0	Advanced
104	lipistad.mp.	1	Advanced
105	lipitor.mp.	161	Advanced
106	lipovast.mp.	0	Advanced
107	liprimar.mp.	1	Advanced
108	liptin.mp.	1	Advanced
109	liptonorm.mp.	0	Advanced
110	lorvaten.mp.	0	Advanced
111	lowlipen.mp.	0	Advanced
112	nelibat.mp.	0	Advanced
113	obradon.mp.	0	Advanced
114	omegastatin.mp.	0	Advanced
115	orbeos.mp.	0	Advanced
116	orvasta.mp.	0	Advanced
117	pd 13429838a.mp.	0	Advanced
118	pd13429838a.mp.	0	Advanced
119	prevencor.mp.	2	Advanced
120	provicard.mp.	0	Advanced

121	rafitin.mp.	0	Advanced
122	rotacor.mp.	0	Advanced
123	rotova.mp.	0	Advanced
124	"s 05153".mp.	0	Advanced
125	s05153.mp.	1	Advanced
126	sortis.mp.	18	Advanced
127	statorva.mp.	0	Advanced
128	storvas.mp.	0	Advanced
129	tahor.mp.	2	Advanced
130	thervan.mp.	0	Advanced
131	torvacard.mp.	0	Advanced
132	torvachol.mp.	0	Advanced
133	torvacol.mp.	0	Advanced
134	torvalipin.mp.	0	Advanced
135	torvaplus.mp.	0	Advanced
136	torvarin.mp.	0	Advanced
137	torvas.mp.	0	Advanced
138	torvast.mp.	9	Advanced
139	torvastin.mp.	0	Advanced
140	torvatec.mp.	0	Advanced
141	torvazin.mp.	0	Advanced
142	totalip.mp.	1	Advanced
143	tulip.mp.	137	Advanced
144	vastat.mp.	0	Advanced
145	vastatin.mp.	11	Advanced
146	vastazor.mp.	0	Advanced

147	voredanin.mp.	0	Advanced
148	xarator.mp.	0	Advanced
149	xolister–all.mp.	0	Advanced
150	ym 548.mp.	0	Advanced
151	ym548.mp.	0	Advanced
152	zarastin.mp.	0	Advanced
153	zarator.mp.	6	Advanced
154	coupet.mp.	0	Advanced
155	crestor.mp.	144	Advanced
156	epri.mp.	1	Advanced
157	ezallor.mp.	0	Advanced
158	"hgp 0816".mp.	0	Advanced
159	hgp0816.mp.	8	Advanced
160	mertenil.mp.	1	Advanced
161	provisacor.mp.	0	Advanced
162	ropitor.mp.	0	Advanced
163	rostat.mp.	0	Advanced
164	rosudia.mp.	0	Advanced
165	rosumop.mp.	0	Advanced
166	rosuvador.mp.	0	Advanced
167	rosuvas.mp.	7	Advanced
168	rosuvastatin.mp.	3018	Advanced
169	rosuvastatina.mp.	13	Advanced
170	rosuvastatine.mp.	12	Advanced
171	roswera.mp.	0	Advanced
172	roxera.mp.	0	Advanced

173	rozuva–teva.mp.	0	Advanced
174	s 4522.mp.	0	Advanced
175	s4522.mp.	0	Advanced
176	simestat.mp.	0	Advanced
177	sorvasta.mp.	1	Advanced
178	visacor.mp.	10	Advanced
179	xeter.mp.	0	Advanced
180	zahron.mp.	0	Advanced
181	zaranta.mp.	0	Advanced
182	zd 4522.mp.	1	Advanced
183	zd4522.mp.	25	Advanced
184	alipza.mp.	0	Advanced
185	itavastatin.mp.	1	Advanced
186	lippiza.mp.	0	Advanced
187	livalo.mp.	28	Advanced
188	livazo.mp.	3	Advanced
189	nikita.mp.	1	Advanced
190	nisvastatin.mp.	0	Advanced
191	nk 104.mp.	37	Advanced
192	nk104.mp.	0	Advanced
193	nks 104.mp.	0	Advanced
194	nks104.mp.	0	Advanced
195	p 872441.mp.	0	Advanced
196	p872441.mp.	0	Advanced
197	pitava.mp.	1	Advanced
198	pitavastatin.mp.	587	Advanced

199	redevant.mp.	0	Advanced
200	ribar.mp.	0	Advanced
201	trolise.mp.	0	Advanced
202	vezepra.mp.	0	Advanced
203	zypitamag.mp.	0	Advanced
204	aplactin.mp.	0	Advanced
205	astin.mp.	12	Advanced
206	bristacol.mp.	0	Advanced
207	cholespar.mp.	0	Advanced
208	cs 514.mp.	10	Advanced
209	cs514.mp.	0	Advanced
210	dehypotin protect.mp.	0	Advanced
211	elisor.mp.	2	Advanced
212	epatostantin.mp.	0	Advanced
213	eptastatin.mp.	1	Advanced
214	eptastatine.mp.	0	Advanced
215	kenstatin.mp.	0	Advanced
216	lipemol.mp.	0	Advanced
217	lipidal.mp.	0	Advanced
218	liplat.mp.	0	Advanced
219	lipostat.mp.	2	Advanced
220	liprevil.mp.	0	Advanced
221	maxudin.mp.	0	Advanced
222	mevalotin.mp.	5	Advanced
223	minuscol.mp.	0	Advanced
224	novales.mp.	0	Advanced

225	prareduct.mp.	1	Advanced
226	prascolend.mp.	0	Advanced
227	prastan.mp.	0	Advanced
228	prasterol.mp.	0	Advanced
229	prava.mp.	8	Advanced
230	pravachol.mp.	17	Advanced
231	pravacol.mp.	0	Advanced
232	pravalam.mp.	0	Advanced
233	pravalich.mp.	0	Advanced
234	pravaselect.mp.	0	Advanced
235	pravasin.mp.	0	Advanced
236	pravasine.mp.	0	Advanced
237	pravastatin.mp.	2052	Advanced
238	pravastatine.mp.	17	Advanced
239	pravator.mp.	0	Advanced
240	pravyl.mp.	0	Advanced
241	rms 431.mp.	0	Advanced
242	sanaprav.mp.	0	Advanced
243	selectin.mp.	2204	Advanced
244	selektine.mp.	0	Advanced
245	selipran.mp.	0	Advanced
246	sq 31000.mp.	0	Advanced
247	sq31000.mp.	0	Advanced
248	stanidine.mp.	0	Advanced
249	vasopran.mp.	0	Advanced
250	vasten.mp.	2	Advanced

251	versatab.mp.	0	Advanced
252	xipral.mp.	0	Advanced
253	alcosin.mp.	0	Advanced
254	antichol.mp.	1	Advanced
255	apo−simva.mp.	0	Advanced
256	avastinee.mp.	0	Advanced
257	belmalip.mp.	0	Advanced
258	cholemed.mp.	0	Advanced
259	cholestat.mp.	0	Advanced
260	clinfar.mp.	1	Advanced
261	colastatina.mp.	0	Advanced
262	coledis.mp.	0	Advanced
263	colemin.mp.	2	Advanced
264	colemin forte.mp.	1	Advanced
265	colestricon.mp.	0	Advanced
266	corolin.mp.	0	Advanced
267	corsim.mp.	0	Advanced
268	covastin.mp.	0	Advanced
269	denan.mp.	0	Advanced
270	epistatin.mp.	1	Advanced
271	esvat.mp.	0	Advanced
272	eucor.mp.	1	Advanced
273	flolipid.mp.	0	Advanced
274	glipal.mp.	0	Advanced
275	ifistatin.mp.	0	Advanced
276	ipramid.mp.	0	Advanced

277	jabastatina.mp.	0	Advanced
278	kavelor.mp.	0	Advanced
279	klonastin.mp.	0	Advanced
280	kolestevan.mp.	0	Advanced
281	kymazol.mp.	0	Advanced
282	l 644128.mp.	0	Advanced
283	l644128.mp.	0	Advanced
284	labistatin.mp.	0	Advanced
285	lepur.mp.	0	Advanced
286	lip−down.mp.	0	Advanced
287	lipaxin.mp.	0	Advanced
288	lipcut.mp.	0	Advanced
289	lipecor.mp.	0	Advanced
290	lipex.mp.	0	Advanced
291	lipinorm.mp.	0	Advanced
292	lipomin.mp.	0	Advanced
293	liponorm.mp.	0	Advanced
294	liporex.mp.	0	Advanced
295	lipovas.mp.	0	Advanced
296	lipterra.mp.	0	Advanced
297	lodales.mp.	1	Advanced
298	lowcholid.mp.	0	Advanced
299	medipo.mp.	0	Advanced
300	medistatin.mp.	0	Advanced
301	mersivas.mp.	0	Advanced
302	"mk 0733".mp.	3	Advanced

303	mk 733.mp.	18	Advanced
304	mk0733.mp.	0	Advanced
305	mk733.mp.	1	Advanced
306	nezatin.mp.	0	Advanced
307	nitastin.mp.	0	Advanced
308	nivelipol.mp.	0	Advanced
309	nor−vastina.mp.	0	Advanced
310	normofat.mp.	1	Advanced
311	nyzoc.mp.	0	Advanced
312	omistat.mp.	0	Advanced
313	orovas.mp.	0	Advanced
314	pantok.mp.	0	Advanced
315	placol.mp.	1	Advanced
316	pravostin.mp.	0	Advanced
317	priacin.mp.	0	Advanced
318	protecta.mp.	5	Advanced
319	ranzolont.mp.	0	Advanced
320	raptor.mp.	33	Advanced
321	rechol.mp.	0	Advanced
322	redusterol.mp.	0	Advanced
323	rendapid.mp.	0	Advanced
324	simar.mp.	1	Advanced
325	simbado.mp.	0	Advanced
326	simbatrix.mp.	0	Advanced
327	simcard.mp.	4	Advanced
328	simchol.mp.	0	Advanced

329	simcovas.mp.	0	Advanced
330	simorion.mp.	0	Advanced
331	simovil.mp.	0	Advanced
332	simratio.mp.	0	Advanced
333	simtan.mp.	0	Advanced
334	simtin.mp.	0	Advanced
335	simva.mp.	65	Advanced
336	simvabeta.mp.	0	Advanced
337	simvacard.mp.	0	Advanced
338	simvachol.mp.	0	Advanced
339	simvacol.mp.	0	Advanced
340	simvacor.mp.	0	Advanced
341	simvador.mp.	1	Advanced
342	simvadura.mp.	0	Advanced
343	simvagen.mp.	0	Advanced
344	simvahex.mp.	0	Advanced
345	simvahexal.mp.	2	Advanced
346	simvalid.mp.	0	Advanced
347	simvalimit.mp.	0	Advanced
348	simvalord.mp.	0	Advanced
349	simvasim.mp.	0	Advanced
350	simvastar.mp.	0	Advanced
351	simvastatin.mp.	4191	Advanced
352	simvastatina.mp.	43	Advanced
353	simvastatine.mp.	27	Advanced
354	simvasterol.mp.	0	Advanced

355	simvata.mp.	0	Advanced
356	simvatin.mp.	0	Advanced
357	simvax.mp.	0	Advanced
358	simvor.mp.	0	Advanced
359	simvotin.mp.	0	Advanced
360	sincol.mp.	0	Advanced
361	sinpor.mp.	0	Advanced
362	sintenal.mp.	0	Advanced
363	sinvacor.mp.	2	Advanced
364	sinvalip.mp.	0	Advanced
365	sinvastatin.mp.	6	Advanced
366	sinvinolin.mp.	0	Advanced
367	sivastin.mp.	6	Advanced
368	sivatin.mp.	0	Advanced
369	starzoco.mp.	0	Advanced
370	stasiva.mp.	0	Advanced
371	statinum.mp.	0	Advanced
372	stativer.mp.	0	Advanced
373	synvinolin.mp.	6	Advanced
374	torio.mp.	0	Advanced
375	tremital.mp.	0	Advanced
376	valemia.mp.	0	Advanced
377	vasilip.mp.	0	Advanced
378	vasotenal.mp.	0	Advanced
379	vastan.mp.	0	Advanced
380	vastatin.mp.	11	Advanced

381	vastin.mp.	0	Advanced
382	vazim.mp.	0	Advanced
383	velastatin.mp.	0	Advanced
384	velkastatin.mp.	0	Advanced
385	velostatin.mp.	0	Advanced
386	veristin.mp.	0	Advanced
387	veritrat.mp.	0	Advanced
388	vidastat.mp.	0	Advanced
389	ximve.mp.	0	Advanced
390	xipocol.mp.	0	Advanced
391	zeplan.mp.	0	Advanced
392	zimmex.mp.	0	Advanced
393	zocor.mp.	90	Advanced
394	zocord.mp.	6	Advanced
395	zorced.mp.	0	Advanced
396	zosta.mp.	0	Advanced
397	zovar.mp.	0	Advanced
398	zovast.mp.	0	Advanced
399	altocor.mp.	0	Advanced
400	altoprev.mp.	0	Advanced
401	artein.mp.	0	Advanced
402	belvas.mp.	0	Advanced
403	birotin.mp.	0	Advanced
404	cholestra.mp.	0	Advanced
405	cysin.mp.	0	Advanced
406	ellanco.mp.	0	Advanced

407	elstatin.mp.	0	Advanced
408	l 654969.mp.	0	Advanced
409	lipdip.mp.	0	Advanced
410	lipivas.mp.	1	Advanced
411	lofacol.mp.	0	Advanced
412	lomar.mp.	0	Advanced
413	lostatin.mp.	0	Advanced
414	lovacel.mp.	0	Advanced
415	lovacol.mp.	0	Advanced
416	lovahexal.mp.	0	Advanced
417	lovalip.mp.	0	Advanced
418	lovalord.mp.	0	Advanced
419	lovastan.mp.	0	Advanced
420	lovastatin.mp.	1007	Advanced
421	lovasterol.mp.	0	Advanced
422	lovastin.mp.	1	Advanced
423	lovatadin.mp.	0	Advanced
424	lowachol.mp.	0	Advanced
425	lozutin.mp.	0	Advanced
426	medostatin.mp.	0	Advanced
427	mevacor.mp.	16	Advanced
428	meverstin.mp.	0	Advanced
429	mevinacor.mp.	2	Advanced
430	"mk 0803".mp.	0	Advanced
431	mk 803.mp.	1	Advanced
432	mk0803.mp.	0	Advanced

433	mk803.mp.	0	Advanced
434	monacolin K.mp.	70	Advanced
435	monakolin k.mp.	2	Advanced
436	msd 803.mp.	0	Advanced
437	neolipid.mp.	0	Advanced
438	nergadan.mp.	0	Advanced
439	ovasta.mp.	0	Advanced
440	rodatin.mp.	0	Advanced
441	rovacor.mp.	0	Advanced
442	taucor.mp.	0	Advanced
443	almastatin.mp.	0	Advanced
444	canef.mp.	0	Advanced
445	cardiol.mp.	118	Advanced
446	cranoc.mp.	1	Advanced
447	digardil.mp.	0	Advanced
448	digaril.mp.	0	Advanced
449	fluindostatin.mp.	110	Advanced
450	fluvastatin.mp.	727	Advanced
451	fluvastatina.mp.	8	Advanced
452	fluvastatine.mp.	2	Advanced
453	fractal.mp.	235	Advanced
454	leposit prolib.mp.	0	Advanced
455	lescol.mp.	74	Advanced
456	leucol.mp.	0	Advanced
457	lipaxan.mp.	0	Advanced
458	lipaxin.mp.	0	Advanced

459	liposit.mp.	0	Advanced
460	lochol.mp.	1	Advanced
461	locol.mp.	9	Advanced
462	lymetel.mp.	0	Advanced
463	primesin.mp.	0	Advanced
464	primexin.mp.	0	Advanced
465	sri 62320.mp.	0	Advanced
466	sri62320.mp.	0	Advanced
467	vaditon.mp.	0	Advanced
468	vaditon prolib.mp.	0	Advanced
469	vastin.mp.	0	Advanced
470	xu 62 320.mp.	2	Advanced
471	xu 62320.mp.	0	Advanced
472	xu62320.mp.	0	Advanced
473	anj 900.mp.	0	Advanced
474	anj900.mp.	1	Advanced
475	apophage.mp.	0	Advanced
476	aron.mp.	21	Advanced
477	benofomin.mp.	0	Advanced
478	dabex.mp.	0	Advanced
479	denkaform.mp.	0	Advanced
480	deson.mp.	0	Advanced
481	dextin.mp.	0	Advanced
482	diabetase.mp.	0	Advanced
483	diabetase s.mp.	0	Advanced
484	diabetformin.mp.	0	Advanced

485	diabetmin.mp.	2	Advanced
486	diabetosan.mp.	0	Advanced
487	diabex.mp.	8	Advanced
488	diafat.mp.	0	Advanced
489	diaformin.mp.	1	Advanced
490	diaformina.mp.	0	Advanced
491	diametin.mp.	0	Advanced
492	diamin.mp.	1	Advanced
493	dianben.mp.	8	Advanced
494	diformin.mp.	3	Advanced
495	dimefor.mp.	1	Advanced
496	dimethylbiguanide.mp.	22	Advanced
497	dimethyldiguanide.mp.	8	Advanced
498	dmgg.mp.	2	Advanced
499	dybis.mp.	0	Advanced
500	"efb 0027".mp.	0	Advanced
501	efb0027.mp.	3	Advanced
502	eraphage.mp.	0	Advanced
503	espa−formin.mp.	0	Advanced
504	euform retard.mp.	0	Advanced
505	fluamine.mp.	0	Advanced
506	flumamine.mp.	1	Advanced
507	fornidd.mp.	0	Advanced
508	fortamet.mp.	4	Advanced
509	glafornil.mp.	1	Advanced
510	glibudon.mp.	0	Advanced

511	glifage.mp.	3	Advanced
512	gliguanid.mp.	0	Advanced
513	glucaminol.mp.	0	Advanced
514	glucofage.mp.	0	Advanced
515	glucofago.mp.	0	Advanced
516	glucoform.mp.	0	Advanced
517	glucoformin.mp.	0	Advanced
518	glucohexal.mp.	0	Advanced
519	glucoless.mp.	0	Advanced
520	glucomet.mp.	0	Advanced
521	glucomin.mp.	0	Advanced
522	glucomine.mp.	0	Advanced
523	gluconil.mp.	0	Advanced
524	glucophage.mp.	270	Advanced
525	glucostop.mp.	1	Advanced
526	glucotika.mp.	0	Advanced
527	gludepatic.mp.	0	Advanced
528	glufor.mp.	0	Advanced
529	gluformin.mp.	1	Advanced
530	glukophage.mp.	0	Advanced
531	glumeformin.mp.	0	Advanced
532	glumet.mp.	0	Advanced
533	glumetza.mp.	6	Advanced
534	glupa.mp.	0	Advanced
535	glustress.mp.	0	Advanced
536	glyciphage.mp.	0	Advanced

537	glycomet.mp.	5	Advanced
538	glycon.mp.	1	Advanced
539	glycoran.mp.	2	Advanced
540	glyformin.mp.	0	Advanced
541	glymet.mp.	0	Advanced
542	haurymellin.mp.	0	Advanced
543	hipoglucin.mp.	0	Advanced
544	i–max.mp.	22	Advanced
545	islotin.mp.	0	Advanced
546	jesacrin.mp.	0	Advanced
547	juformin.mp.	0	Advanced
548	la 6023.mp.	2	Advanced
549	la6023.mp.	0	Advanced
550	lyomet.mp.	0	Advanced
551	maformin.mp.	0	Advanced
552	meglucon.mp.	0	Advanced
553	meguan.mp.	0	Advanced
554	melbin.mp.	1	Advanced
555	melformin.mp.	0	Advanced
556	mellittin.mp.	0	Advanced
557	merckformin.mp.	0	Advanced
558	mescorit.mp.	0	Advanced
559	metaformin.mp.	5	Advanced
560	metfogamma.mp.	0	Advanced
561	metfoliquid geriasan.mp.	0	Advanced
562	metforal.mp.	10	Advanced

563	metformax.mp.	1	Advanced
564	metformin.mp.	14088	Advanced
565	metformina.mp.	90	Advanced
566	metformine.mp.	90	Advanced
567	methformin.mp.	1	Advanced
568	metiguanide.mp.	0	Advanced
569	metomin.mp.	0	Advanced
570	metphormin.mp.	0	Advanced
571	miformin.mp.	0	Advanced
572	dimethylguanylguanide.mp.	0	Advanced
573	dimethylguanylguanidine.mp.	1	Advanced
574	dimethyldiguanide.mp.	8	Advanced
575	dimethyl biguanidine.mp.	0	Advanced
576	dimethylbiguanide.mp.	22	Advanced
577	dimethylbiguanidine.mp.	0	Advanced
578	dimethyldiguanide.mp.	8	Advanced
579	dimethylguanylguanidine.mp.	1	Advanced
580	neoform.mp.	2	Advanced
581	newmet.mp.	0	Advanced
582	nndg.mp.	0	Advanced
583	reglus−500.mp.	0	Advanced
584	riomet.mp.	3	Advanced
585	riomet er.mp.	1	Advanced
586	risidon.mp.	2	Advanced
587	rudimet.mp.	0	Advanced
588	siamformet.mp.	0	Advanced

589	siofor.mp.	2	Advanced
590	thiabet.mp.	0	Advanced
591	vimetrol.mp.	0	Advanced
592	walaphage.mp.	0	Advanced
593	absorcol.mp.	0	Advanced
594	adezop.mp.	0	Advanced
595	azibe.mp.	0	Advanced
596	carditimib.mp.	0	Advanced
597	cexado.mp.	0	Advanced
598	cildar.mp.	0	Advanced
599	coltowan.mp.	0	Advanced
600	corintus.mp.	0	Advanced
601	delipid.mp.	4	Advanced
602	egitim.mp.	9	Advanced
603	elanix.mp.	0	Advanced
604	erezel.mp.	0	Advanced
605	esetin.mp.	0	Advanced
606	etibax.mp.	0	Advanced
607	ezdivule.mp.	0	Advanced
608	ezedoc.mp.	0	Advanced
609	ezegelan.mp.	0	Advanced
610	ezegros.mp.	0	Advanced
611	ezehron.mp.	1	Advanced
612	ezelip.mp.	0	Advanced
613	ezemed.mp.	0	Advanced
614	ezen.mp.	1	Advanced

615	ezetad.mp.	0	Advanced
616	ezetebmib.mp.	0	Advanced
617	ezetib.mp.	0	Advanced
618	ezetiben.mp.	0	Advanced
619	ezetimib.mp.	22	Advanced
620	ezetimibe.mp.	2053	Advanced
621	ezetrol.mp.	47	Advanced
622	ezetymib.mp.	0	Advanced
623	ezgal.mp.	0	Advanced
624	ezicor.mp.	0	Advanced
625	ezoleta.mp.	0	Advanced
626	hgp 1404.mp.	0	Advanced
627	hgp1404.mp.	1	Advanced
628	intestat.mp.	0	Advanced
629	kobey.mp.	0	Advanced
630	lipegis.mp.	0	Advanced
631	lipobon.mp.	0	Advanced
632	mibecol.mp.	0	Advanced
633	mibezet.mp.	0	Advanced
634	noveze.mp.	0	Advanced
635	noxetib.mp.	0	Advanced
636	olufsent.mp.	0	Advanced
637	quver.mp.	0	Advanced
638	sch 58235.mp.	18	Advanced
639	sch58235.mp.	1	Advanced
640	strami.mp.	0	Advanced

641	symezet.mp.	0	Advanced
642	tezzimi.mp.	0	Advanced
643	tritilz.mp.	0	Advanced
644	trizibe.mp.	0	Advanced
645	viemm.mp.	0	Advanced
646	vyeve.mp.	0	Advanced
647	ylerse.mp.	0	Advanced
648	zertya.mp.	0	Advanced
649	zetaxyl.mp.	0	Advanced
650	zetia.mp.	27	Advanced
651	zetiraf.mp.	0	Advanced
652	zetratak.mp.	0	Advanced
653	zetratak−s.mp.	0	Advanced
654	zient.mp.	0	Advanced
655	alirocumab.mp.	477	Advanced
656	praluent.mp.	13	Advanced
657	regn 727.mp.	2	Advanced
658	regn727.mp.	67	Advanced
659	sar 236553.mp.	0	Advanced
660	sar236553.mp.	66	Advanced
661	evolucumab.mp.	4	Advanced
662	amg 145.mp.	109	Advanced
663	amg145.mp.	12	Advanced
664	repatha.mp.	29	Advanced
665	"sal 003".mp.	0	Advanced
666	sal003.mp.	2	Advanced

667	aln 60212.mp.	5	Advanced
668	aln pcssc.mp.	8	Advanced
669	aln60212.mp.	0	Advanced
670	inclisiran.mp.	133	Advanced
671	kjx 839.mp.	0	Advanced
672	kjx839.mp.	12	Advanced
673	leqvio.mp.	6	Advanced
674	antara.mp.	8	Advanced
675	apo−feno−micro.mp.	0	Advanced
676	apteor.mp.	0	Advanced
677	aterolis.mp.	0	Advanced
678	biofibrat.mp.	0	Advanced
679	bisterol sr.mp.	0	Advanced
680	catalip.mp.	0	Advanced
681	climage.mp.	0	Advanced
682	controlip.mp.	0	Advanced
683	durafenat.mp.	0	Advanced
684	elipsia.mp.	1	Advanced
685	evothyl.mp.	0	Advanced
686	febira.mp.	0	Advanced
687	fegenor.mp.	0	Advanced
688	felosma.mp.	0	Advanced
689	fenardin.mp.	0	Advanced
690	fenobrat.mp.	0	Advanced
691	fenobrate.mp.	0	Advanced
692	fenochol.mp.	0	Advanced

693	fenofanton.mp.	0	Advanced
694	fenofibrate.mp.	1146	Advanced
695	fenofix.mp.	0	Advanced
696	fenogal.mp.	1	Advanced
697	fenoglide.mp.	0	Advanced
698	fenolip.mp.	0	Advanced
699	fenomax.mp.	0	Advanced
700	fenosup lidose.mp.	0	Advanced
701	fenoswiss.mp.	0	Advanced
702	fenox.mp.	0	Advanced
703	fibrafen.mp.	0	Advanced
704	fulcro.mp.	0	Advanced
705	fulcrosupra.mp.	0	Advanced
706	grofibrat.mp.	2	Advanced
707	"grs 001".mp.	4	Advanced
708	grs001.mp.	0	Advanced
709	hyperchol.mp.	0	Advanced
710	katalip.mp.	0	Advanced
711	lexemin.mp.	0	Advanced
712	lifenoz−u.mp.	0	Advanced
713	lipanthyl.mp.	21	Advanced
714	lipanthyl.mp.	21	Advanced
715	lipanthylnano.mp.	0	Advanced
716	lipantil.mp.	7	Advanced
717	lipantyl.mp.	1	Advanced
718	liparison.mp.	0	Advanced

719	lipcor.mp.	0	Advanced
720	liperial.mp.	1	Advanced
721	lipidax.mp.	0	Advanced
722	lipidil.mp.	14	Advanced
723	lipilfen.mp.	3	Advanced
724	lipilo.mp.	0	Advanced
725	lipirex.mp.	0	Advanced
726	lipoclar.mp.	1	Advanced
727	lipofen.mp.	0	Advanced
728	lipofene.mp.	0	Advanced
729	lipofib.mp.	0	Advanced
730	lipolin.mp.	0	Advanced
731	liposit.mp.	0	Advanced
732	lipsin.mp.	0	Advanced
733	livesan ge.mp.	0	Advanced
734	lofibra.mp.	2	Advanced
735	nopid 200.mp.	0	Advanced
736	normalip.mp.	0	Advanced
737	nubrex.mp.	0	Advanced
738	procetofen.mp.	7	Advanced
739	procetofenate.mp.	0	Advanced
740	procetofene.mp.	6	Advanced
741	procetofen.mp.	7	Advanced
742	qualipantyl.mp.	0	Advanced
743	rapidil.mp.	0	Advanced
744	redose 200.mp.	0	Advanced

745	rorit.mp.	0	Advanced
746	rp 1824.mp.	0	Advanced
747	rp1824.mp.	0	Advanced
748	secalip.mp.	0	Advanced
749	sigurtil.mp.	0	Advanced
750	supralip.mp.	2	Advanced
751	suprelip.mp.	0	Advanced
752	trichol.mp.	1	Advanced
753	tricor.mp.	16	Advanced
754	triglide.mp.	0	Advanced
755	trolip.mp.	0	Advanced
756	xafenor.mp.	1	Advanced
757	zerlubron.mp.	0	Advanced
758	ziglip.mp.	0	Advanced
759	zigotrig.mp.	0	Advanced
760	zumafib.mp.	0	Advanced
761	ausgem.mp.	0	Advanced
762	bolutol.mp.	0	Advanced
763	brozil.mp.	0	Advanced
764	chlorestrol.mp.	0	Advanced
765	cholespid.mp.	0	Advanced
766	ci 719.mp.	41	Advanced
767	ci719.mp.	1	Advanced
768	clearol.mp.	0	Advanced
769	decrelip.mp.	0	Advanced
770	detrichol.mp.	0	Advanced

771	elmogan.mp.	0	Advanced
772	fetinor.mp.	0	Advanced
773	fibralip.mp.	0	Advanced
774	fibrocit.mp.	0	Advanced
775	gedum.mp.	0	Advanced
776	gemfi.mp.	0	Advanced
777	gemfibril.mp.	0	Advanced
778	gemfibromax.mp.	0	Advanced
779	gemfibrozil.mp.	557	Advanced
780	gemfibrozil.mp.	557	Advanced
781	gemfibrozilo.mp.	8	Advanced
782	gemhexal.mp.	0	Advanced
783	gemizol.mp.	0	Advanced
784	gemlipid.mp.	0	Advanced
785	gemnpid.mp.	0	Advanced
786	gemzil.mp.	0	Advanced
787	genfibrozil.mp.	1	Advanced
788	gevilon.mp.	0	Advanced
789	gevilon uno.mp.	0	Advanced
790	gozid.mp.	0	Advanced
791	grifogemzilo.mp.	0	Advanced
792	hidil.mp.	0	Advanced
793	hipolixan.mp.	0	Advanced
794	ipolipid.mp.	0	Advanced
795	jezil.mp.	0	Advanced
796	lanaterom.mp.	0	Advanced

797	lifibron.mp.	0	Advanced
798	lipazil.mp.	0	Advanced
799	lipidys.mp.	0	Advanced
800	lipigem.mp.	0	Advanced
801	lipira.mp.	0	Advanced
802	lipison.mp.	0	Advanced
803	lipistorol.mp.	0	Advanced
804	lipizyl.mp.	0	Advanced
805	lipofor.mp.	0	Advanced
806	lipolo.mp.	0	Advanced
807	lipostorol.mp.	0	Advanced
808	lipox gemfi.mp.	0	Advanced
809	lipozid.mp.	0	Advanced
810	lipozil.mp.	0	Advanced
811	lipur.mp.	0	Advanced
812	litarek.mp.	0	Advanced
813	lopid.mp.	22	Advanced
814	lowin.mp.	8	Advanced
815	low−lip.mp.	0	Advanced
816	manobrozil.mp.	0	Advanced
817	mariston.mp.	0	Advanced
818	mersikol.mp.	0	Advanced
819	normolip.mp.	1	Advanced
820	panazil.mp.	0	Advanced
821	pilder.mp.	0	Advanced
822	polyxit.mp.	0	Advanced

823	progemzal.mp.	0	Advanced
824	q8x02027x3.mp.	0	Advanced
825	recozil.mp.	0	Advanced
826	reducel.mp.	0	Advanced
827	regulip.mp.	0	Advanced
828	synbrozil.mp.	0	Advanced
829	trialmin.mp.	0	Advanced
830	triglizil.mp.	0	Advanced
831	uragem.mp.	0	Advanced
832	zilop.mp.	0	Advanced
833	or/1-832	82038	Advanced
834	exp aneurysm, ruptured/	306	Advanced
835	exp aortic aneurysm/	1171	Advanced
836	exp aortic dissection/	19	Advanced
837	aneurysm?.mp.	5072	Advanced
838	aortic rupture*.mp.	156	Advanced
839	loeys-dietz.mp.	6	Advanced
840	aortic dissection?.mp.	580	Advanced
841	aorta dissection?.mp.	35	Advanced
842	aortic dilatation?.mp.	68	Advanced
843	aortic ectasia?.mp.	3	Advanced
844	exp Aorta/su [Surgery]	6	Advanced
845	((aorta or aortic) and (surgery or surgical)).mp.	7307	Advanced
846	(Bentall adj2 (repair or replacement or surgery)).mp.	9	Advanced
847	(David adj2 (repair or surgery)).mp.	1	Advanced
848	(aortic root adj2 (repair or replacement)).mp.	29	Advanced

849	(Yacoub adj2 (repair or surgery)).mp.	0	Advanced
850	(hemiyacoub adj2 (repair or surgery)).mp.	0	Advanced
851	frozen elephant trunk surgery.mp.	0	Advanced
852	(hemiarch adj2 (repair or replacement)).mp.	11	Advanced
853	Marfan Syndrome/	68	Advanced
854	(marfan* adj2 syndrome*).mp.	138	Advanced
855	aortic h?ematoma.mp.	3	Advanced
856	aortic ulcer*.mp.	8	Advanced
857	tevar.mp.	88	Advanced
858	evar.mp.	351	Advanced
859	aortic endoprosthesis.mp.	3	Advanced
860	(aortic adj2 replacement).mp.	2079	Advanced
861	aortic growth.mp.	13	Advanced
862	or/834-861	11445	Advanced
863	833 and 862	424	Advanced

EBM Reviews - Cochrane Database of Systematic Reviews <2005 to March 19, 2025>

#	Searches	Results	Type
1	antihyperlipemic*.mp.	2	Advanced
2	Antilip?emic*.mp.	7	Advanced
3	(hypolipidemic adj2 (agent? or drug?)).mp.	10	Advanced
4	(anticholesteremic adj2 (agent? or drug?)).mp.	13	Advanced
5	(antihypertriglyceridic adj2 (agent? or drug?)).mp.	0	Advanced
6	(antilip?emia adj2 (agent? or drug?)).mp.	0	Advanced
7	(antilipidemic adj2 (agent? or drug?)).mp.	0	Advanced
8	(hypolip?emic adj2 (agent? or drug?)).mp.	0	Advanced

9	(hypotriglyceridic adj2 (agent? or drug?)).mp.	0	Advanced
10	(lipid depressing adj2 (agent? or drug?)).mp.	0	Advanced
11	(lipid lowering adj2 (agent? or drug?)).mp.	65	Advanced
12	Anticholesteremic?.mp.	13	Advanced
13	(cholesterol adj2 inhibitor?).mp.	12	Advanced
14	(hypocholester?emic adj2 (agent? or drug?)).mp.	1	Advanced
15	(Hypocholesterol?emic adj2 (agent? or drug?)).mp.	2	Advanced
16	(antihypercholesteremic adj2 (agent? or drug?)).mp.	0	Advanced
17	hmg coa reductase inhibitor?.mp.	34	Advanced
18	hmg coenzyme a reductase inhibitor?.mp.	0	Advanced
19	hydroxymethylglutaryl coa reductase inhibitor?.mp.	43	Advanced
20	hydroxymethylglutaryl-coa inhibitor?.mp.	1	Advanced
21	hydroxymethylglutaryl-coenzyme a inhibitor?.mp.	1	Advanced
22	statin?.mp.	895	Advanced
23	pcsk9 inhibitor?.mp.	4	Advanced
24	fatty acid synthesis inhibitor?.mp.	0	Advanced
25	lipocaic?.mp.	0	Advanced
26	(lipotropic adj2 (agent? or drug? or factor?)).mp.	0	Advanced
27	Cholesterol.mp.	627	Advanced
28	Epicholesterol.mp.	1	Advanced
29	Hyperlip?emia?.mp.	33	Advanced
30	Hyperlipid?emia?.mp.	253	Advanced
31	Lipemia?.mp.	4	Advanced
32	Lipid?emia?.mp.	4	Advanced
33	Hypercholester?emia?.mp.	28	Advanced
34	Hypercholesterol?emia?.mp.	146	Advanced

35	Hypercholesterin?emia.mp.	0	Advanced
36	Hypertriglycerid?emia?.mp.	86	Advanced
37	Cholesteremia.mp.	0	Advanced
38	Cholesterinemia.mp.	0	Advanced
39	Cholesterolemia.mp.	1	Advanced
40	Atorvastatin.mp.	74	Advanced
41	a 2581175.mp.	0	Advanced
42	a2581175.mp.	0	Advanced
43	amicor.mp.	0	Advanced
44	antorcin.mp.	0	Advanced
45	apo−atorva.mp.	0	Advanced
46	arkas.mp.	0	Advanced
47	artas.mp.	0	Advanced
48	ascord.mp.	0	Advanced
49	astator.mp.	0	Advanced
50	atacor.mp.	0	Advanced
51	atofast.mp.	0	Advanced
52	ator.mp.	0	Advanced
53	ator−chol.mp.	0	Advanced
54	atorab.mp.	0	Advanced
55	atorbir.mp.	0	Advanced
56	atorcor.mp.	0	Advanced
57	atoridor.mp.	0	Advanced
58	atorin.mp.	0	Advanced
59	atoris.mp.	0	Advanced
60	atorlip.mp.	3	Advanced

61	atormax.mp.	0	Advanced
62	atorstad.mp.	0	Advanced
63	atorstan.mp.	0	Advanced
64	atorstat.mp.	0	Advanced
65	atorvadivid.mp.	0	Advanced
66	atorvagen.mp.	0	Advanced
67	atorval.mp.	0	Advanced
68	atorvalan.mp.	0	Advanced
69	atorvalet.mp.	0	Advanced
70	atorvaliq.mp.	0	Advanced
71	atorvanox.mp.	0	Advanced
72	atorvasterol.mp.	0	Advanced
73	atorvastine.mp.	0	Advanced
74	atorvox.mp.	0	Advanced
75	atorzem.mp.	0	Advanced
76	atostat.mp.	0	Advanced
77	atostin.mp.	0	Advanced
78	atovans.mp.	0	Advanced
79	atovarol.mp.	3	Advanced
80	atractin.mp.	0	Advanced
81	atraven.mp.	0	Advanced
82	atrocell.mp.	0	Advanced
83	atrost.mp.	0	Advanced
84	atrox.mp.	1	Advanced
85	calipra.mp.	0	Advanced
86	card−ok.mp.	0	Advanced

87	cardiostyl.mp.	0	Advanced
88	cardyl.mp.	3	Advanced
89	ci 981.mp.	16	Advanced
90	ci981.mp.	6	Advanced
91	decholest.mp.	0	Advanced
92	delipost.mp.	0	Advanced
93	gletor.mp.	0	Advanced
94	glustar.mp.	3	Advanced
95	lambrinex.mp.	0	Advanced
96	larus.mp.	0	Advanced
97	latrovin.mp.	0	Advanced
98	lipibec.mp.	5	Advanced
99	lipigan.mp.	0	Advanced
100	lipimed.mp.	0	Advanced
101	lipistad.mp.	0	Advanced
102	lipitor.mp.	12	Advanced
103	lipovast.mp.	0	Advanced
104	liprimar.mp.	2	Advanced
105	liptin.mp.	1	Advanced
106	liptonorm.mp.	3	Advanced
107	lorvaten.mp.	0	Advanced
108	lowlipen.mp.	3	Advanced
109	nelibat.mp.	0	Advanced
110	obradon.mp.	0	Advanced
111	omegastatin.mp.	0	Advanced
112	orbeos.mp.	0	Advanced

113	orvasta.mp.	0	Advanced
114	pd 13429838a.mp.	0	Advanced
115	pd13429838a.mp.	0	Advanced
116	prevencor.mp.	1	Advanced
117	provicard.mp.	0	Advanced
118	rafitin.mp.	0	Advanced
119	rotacor.mp.	0	Advanced
120	rotova.mp.	0	Advanced
121	"s 05153".mp.	0	Advanced
122	s05153.mp.	0	Advanced
123	sortis.mp.	6	Advanced
124	statorva.mp.	0	Advanced
125	storvas.mp.	3	Advanced
126	tahor.mp.	6	Advanced
127	thervan.mp.	0	Advanced
128	torvacard.mp.	0	Advanced
129	torvachol.mp.	0	Advanced
130	torvacol.mp.	0	Advanced
131	torvalipin.mp.	0	Advanced
132	torvaplus.mp.	0	Advanced
133	torvarin.mp.	0	Advanced
134	torvas.mp.	0	Advanced
135	torvast.mp.	6	Advanced
136	torvastin.mp.	0	Advanced
137	torvatec.mp.	0	Advanced
138	torvazin.mp.	0	Advanced

139	totalip.mp.	3	Advanced
140	tulip.mp.	6	Advanced
141	vastat.mp.	0	Advanced
142	vastatin.mp.	1	Advanced
143	vastazor.mp.	0	Advanced
144	voredanin.mp.	0	Advanced
145	xarator.mp.	3	Advanced
146	xolister−all.mp.	0	Advanced
147	ym 548.mp.	6	Advanced
148	ym548.mp.	6	Advanced
149	zarastin.mp.	0	Advanced
150	zarator.mp.	6	Advanced
151	coupet.mp.	0	Advanced
152	crestor.mp.	10	Advanced
153	epri.mp.	0	Advanced
154	ezallor.mp.	0	Advanced
155	"hgp 0816".mp.	0	Advanced
156	hgp0816.mp.	0	Advanced
157	mertenil.mp.	0	Advanced
158	provisacor.mp.	0	Advanced
159	ropitor.mp.	0	Advanced
160	rostat.mp.	0	Advanced
161	rosudia.mp.	0	Advanced
162	rosumop.mp.	0	Advanced
163	rosuvador.mp.	0	Advanced
164	rosuvas.mp.	3	Advanced

165	rosuvastatin.mp.	50	Advanced
166	rosuvastatina.mp.	0	Advanced
167	rosuvastatine.mp.	0	Advanced
168	roswera.mp.	0	Advanced
169	roxera.mp.	0	Advanced
170	rozuva−teva.mp.	0	Advanced
171	s 4522.mp.	6	Advanced
172	s4522.mp.	6	Advanced
173	simestat.mp.	0	Advanced
174	sorvasta.mp.	0	Advanced
175	visacor.mp.	3	Advanced
176	xeter.mp.	0	Advanced
177	zahron.mp.	0	Advanced
178	zaranta.mp.	0	Advanced
179	zd 4522.mp.	6	Advanced
180	zd4522.mp.	6	Advanced
181	alipza.mp.	3	Advanced
182	itavastatin.mp.	5	Advanced
183	lippiza.mp.	2	Advanced
184	livalo.mp.	7	Advanced
185	livazo.mp.	3	Advanced
186	nikita.mp.	5	Advanced
187	nisvastatin.mp.	5	Advanced
188	nk 104.mp.	6	Advanced
189	nk104.mp.	5	Advanced
190	nks 104.mp.	5	Advanced

191	nks104.mp.	5	Advanced
192	p 872441.mp.	2	Advanced
193	p872441.mp.	0	Advanced
194	pitava.mp.	7	Advanced
195	pitavastatin.mp.	28	Advanced
196	redevant.mp.	0	Advanced
197	ribar.mp.	4	Advanced
198	trolise.mp.	2	Advanced
199	vezepra.mp.	3	Advanced
200	zypitamag.mp.	2	Advanced
201	aplactin.mp.	1	Advanced
202	astin.mp.	36	Advanced
203	bristacol.mp.	5	Advanced
204	cholespar.mp.	3	Advanced
205	cs 514.mp.	6	Advanced
206	cs514.mp.	6	Advanced
207	dehypotin protect.mp.	0	Advanced
208	elisor.mp.	6	Advanced
209	epatostantin.mp.	5	Advanced
210	eptastatin.mp.	6	Advanced
211	eptastatine.mp.	3	Advanced
212	kenstatin.mp.	4	Advanced
213	lipemol.mp.	6	Advanced
214	lipidal.mp.	4	Advanced
215	liplat.mp.	6	Advanced
216	lipostat.mp.	9	Advanced

217	liprevil.mp.	4	Advanced
218	maxudin.mp.	1	Advanced
219	mevalotin.mp.	8	Advanced
220	minuscol.mp.	1	Advanced
221	novales.mp.	3	Advanced
222	prareduct.mp.	5	Advanced
223	prascolend.mp.	4	Advanced
224	prastan.mp.	3	Advanced
225	prasterol.mp.	1	Advanced
226	prava.mp.	4	Advanced
227	pravachol.mp.	10	Advanced
228	pravacol.mp.	6	Advanced
229	pravalam.mp.	1	Advanced
230	pravalich.mp.	1	Advanced
231	pravaselect.mp.	4	Advanced
232	pravasin.mp.	8	Advanced
233	pravasine.mp.	4	Advanced
234	pravastatin.mp.	50	Advanced
235	pravastatine.mp.	0	Advanced
236	pravator.mp.	3	Advanced
237	pravyl.mp.	3	Advanced
238	rms 431.mp.	5	Advanced
239	sanaprav.mp.	6	Advanced
240	selectin.mp.	25	Advanced
241	selektine.mp.	6	Advanced
242	selipran.mp.	4	Advanced

243	sq 31000.mp.	6	Advanced
244	sq31000.mp.	6	Advanced
245	stanidine.mp.	3	Advanced
246	vasopran.mp.	3	Advanced
247	vasten.mp.	3	Advanced
248	versatab.mp.	0	Advanced
249	xipral.mp.	4	Advanced
250	alcosin.mp.	0	Advanced
251	antichol.mp.	1	Advanced
252	apo−simva.mp.	0	Advanced
253	avastinee.mp.	3	Advanced
254	belmalip.mp.	1	Advanced
255	cholemed.mp.	0	Advanced
256	cholestat.mp.	3	Advanced
257	clinfar.mp.	3	Advanced
258	colastatina.mp.	3	Advanced
259	coledis.mp.	1	Advanced
260	colemin.mp.	1	Advanced
261	colemin forte.mp.	0	Advanced
262	colestricon.mp.	3	Advanced
263	corolin.mp.	1	Advanced
264	corsim.mp.	0	Advanced
265	covastin.mp.	3	Advanced
266	denan.mp.	5	Advanced
267	epistatin.mp.	5	Advanced
268	esvat.mp.	3	Advanced

269	eucor.mp.	2	Advanced
270	flolipid.mp.	1	Advanced
271	glipal.mp.	0	Advanced
272	ifistatin.mp.	3	Advanced
273	ipramid.mp.	0	Advanced
274	jabastatina.mp.	1	Advanced
275	kavelor.mp.	3	Advanced
276	klonastin.mp.	3	Advanced
277	kolestevan.mp.	3	Advanced
278	kymazol.mp.	0	Advanced
279	l 644128.mp.	3	Advanced
280	l644128.mp.	3	Advanced
281	labistatin.mp.	1	Advanced
282	lepur.mp.	0	Advanced
283	lip−down.mp.	0	Advanced
284	lipaxin.mp.	0	Advanced
285	lipcut.mp.	1	Advanced
286	lipecor.mp.	3	Advanced
287	lipex.mp.	7	Advanced
288	lipinorm.mp.	4	Advanced
289	lipomin.mp.	0	Advanced
290	liponorm.mp.	3	Advanced
291	liporex.mp.	0	Advanced
292	lipovas.mp.	7	Advanced
293	lipterra.mp.	0	Advanced
294	lodales.mp.	6	Advanced

295	lowcholid.mp.	0	Advanced
296	medipo.mp.	6	Advanced
297	medistatin.mp.	0	Advanced
298	mersivas.mp.	3	Advanced
299	"mk 0733".mp.	0	Advanced
300	mk 733.mp.	5	Advanced
301	mk0733.mp.	0	Advanced
302	mk733.mp.	5	Advanced
303	nezatin.mp.	0	Advanced
304	nitastin.mp.	0	Advanced
305	nivelipol.mp.	1	Advanced
306	nor−vastina.mp.	0	Advanced
307	normofat.mp.	3	Advanced
308	nyzoc.mp.	0	Advanced
309	omistat.mp.	0	Advanced
310	orovas.mp.	3	Advanced
311	pantok.mp.	2	Advanced
312	placol.mp.	0	Advanced
313	pravostin.mp.	0	Advanced
314	priacin.mp.	0	Advanced
315	protecta.mp.	0	Advanced
316	ranzolont.mp.	0	Advanced
317	raptor.mp.	3	Advanced
318	rechol.mp.	4	Advanced
319	redusterol.mp.	0	Advanced
320	rendapid.mp.	1	Advanced

321	simar.mp.	0	Advanced
322	simbado.mp.	3	Advanced
323	simbatrix.mp.	0	Advanced
324	simcard.mp.	3	Advanced
325	simchol.mp.	3	Advanced
326	simcovas.mp.	0	Advanced
327	simorion.mp.	0	Advanced
328	simovil.mp.	4	Advanced
329	simratio.mp.	0	Advanced
330	simtan.mp.	0	Advanced
331	simtin.mp.	3	Advanced
332	simva.mp.	1	Advanced
333	simvabeta.mp.	0	Advanced
334	simvacard.mp.	0	Advanced
335	simvachol.mp.	0	Advanced
336	simvacol.mp.	0	Advanced
337	simvacor.mp.	3	Advanced
338	simvador.mp.	0	Advanced
339	simvadura.mp.	0	Advanced
340	simvagen.mp.	0	Advanced
341	simvahex.mp.	3	Advanced
342	simvahexal.mp.	0	Advanced
343	simvalid.mp.	0	Advanced
344	simvalimit.mp.	0	Advanced
345	simvalord.mp.	3	Advanced
346	simvasim.mp.	0	Advanced

347	simvastar.mp.	3	Advanced
348	simvastatin.mp.	88	Advanced
349	simvastatina.mp.	1	Advanced
350	simvastatine.mp.	1	Advanced
351	simvasterol.mp.	0	Advanced
352	simvata.mp.	3	Advanced
353	simvatin.mp.	3	Advanced
354	simvax.mp.	0	Advanced
355	simvor.mp.	3	Advanced
356	simvotin.mp.	3	Advanced
357	sincol.mp.	0	Advanced
358	sinpor.mp.	0	Advanced
359	sintenal.mp.	0	Advanced
360	sinvacor.mp.	6	Advanced
361	sinvalip.mp.	0	Advanced
362	sinvastatin.mp.	3	Advanced
363	sinvinolin.mp.	4	Advanced
364	sivastin.mp.	3	Advanced
365	sivatin.mp.	0	Advanced
366	starzoco.mp.	3	Advanced
367	stasiva.mp.	0	Advanced
368	statinum.mp.	0	Advanced
369	stativer.mp.	0	Advanced
370	synvinolin.mp.	4	Advanced
371	torio.mp.	8	Advanced
372	tremital.mp.	0	Advanced

373	valemia.mp.	3	Advanced
374	vasilip.mp.	3	Advanced
375	vasotenal.mp.	3	Advanced
376	vastan.mp.	0	Advanced
377	vastatin.mp.	1	Advanced
378	vastin.mp.	2	Advanced
379	vazim.mp.	3	Advanced
380	velastatin.mp.	1	Advanced
381	velkastatin.mp.	0	Advanced
382	velostatin.mp.	2	Advanced
383	veristin.mp.	0	Advanced
384	veritrat.mp.	0	Advanced
385	vidastat.mp.	3	Advanced
386	ximve.mp.	0	Advanced
387	xipocol.mp.	0	Advanced
388	zeplan.mp.	0	Advanced
389	zimmex.mp.	3	Advanced
390	zocor.mp.	10	Advanced
391	zocord.mp.	4	Advanced
392	zorced.mp.	1	Advanced
393	zosta.mp.	1	Advanced
394	zovar.mp.	0	Advanced
395	zovast.mp.	3	Advanced
396	altocor.mp.	5	Advanced
397	altoprev.mp.	5	Advanced
398	artein.mp.	5	Advanced

399	belvas.mp.	3	Advanced
400	birotin.mp.	3	Advanced
401	cholestra.mp.	3	Advanced
402	cysin.mp.	3	Advanced
403	ellanco.mp.	3	Advanced
404	elstatin.mp.	3	Advanced
405	l 654969.mp.	3	Advanced
406	lipdip.mp.	3	Advanced
407	lipivas.mp.	4	Advanced
408	lofacol.mp.	3	Advanced
409	lomar.mp.	3	Advanced
410	lostatin.mp.	4	Advanced
411	lovacel.mp.	3	Advanced
412	lovacol.mp.	6	Advanced
413	lovahexal.mp.	1	Advanced
414	lovalip.mp.	4	Advanced
415	lovalord.mp.	3	Advanced
416	lovastan.mp.	3	Advanced
417	lovastatin.mp.	49	Advanced
418	lovasterol.mp.	3	Advanced
419	lovastin.mp.	3	Advanced
420	lovatadin.mp.	3	Advanced
421	lowachol.mp.	3	Advanced
422	lozutin.mp.	3	Advanced
423	medostatin.mp.	8	Advanced
424	mevacor.mp.	9	Advanced

425	meverstin.mp.	3	Advanced
426	mevinacor.mp.	8	Advanced
427	"mk 0803".mp.	2	Advanced
428	mk 803.mp.	5	Advanced
429	mk0803.mp.	4	Advanced
430	mk803.mp.	5	Advanced
431	monacolin K.mp.	1	Advanced
432	monakolin k.mp.	1	Advanced
433	msd 803.mp.	4	Advanced
434	neolipid.mp.	6	Advanced
435	nergadan.mp.	4	Advanced
436	ovasta.mp.	3	Advanced
437	rodatin.mp.	3	Advanced
438	rovacor.mp.	3	Advanced
439	taucor.mp.	3	Advanced
440	almastatin.mp.	0	Advanced
441	canef.mp.	6	Advanced
442	cardiol.mp.	10	Advanced
443	cranoc.mp.	7	Advanced
444	digardil.mp.	0	Advanced
445	digaril.mp.	0	Advanced
446	fluindostatin.mp.	12	Advanced
447	fluvastatin.mp.	44	Advanced
448	fluvastatina.mp.	0	Advanced
449	fluvastatine.mp.	0	Advanced
450	fractal.mp.	4	Advanced

451	leposit prolib.mp.	0	Advanced
452	lescol.mp.	12	Advanced
453	leucol.mp.	5	Advanced
454	lipaxan.mp.	0	Advanced
455	lipaxin.mp.	0	Advanced
456	liposit.mp.	2	Advanced
457	lochol.mp.	4	Advanced
458	locol.mp.	6	Advanced
459	lymetel.mp.	0	Advanced
460	primesin.mp.	0	Advanced
461	primexin.mp.	0	Advanced
462	sri 62320.mp.	4	Advanced
463	sri62320.mp.	4	Advanced
464	vaditon.mp.	0	Advanced
465	vaditon prolib.mp.	0	Advanced
466	vastin.mp.	2	Advanced
467	xu 62 320.mp.	2	Advanced
468	xu 62320.mp.	4	Advanced
469	xu62320.mp.	4	Advanced
470	anj 900.mp.	0	Advanced
471	anj900.mp.	0	Advanced
472	apophage.mp.	0	Advanced
473	aron.mp.	15	Advanced
474	benofomin.mp.	0	Advanced
475	dabex.mp.	0	Advanced
476	denkaform.mp.	0	Advanced

477	deson.mp.	0	Advanced
478	dextin.mp.	0	Advanced
479	diabetase.mp.	0	Advanced
480	diabetase s.mp.	0	Advanced
481	diabetformin.mp.	0	Advanced
482	diabetmin.mp.	0	Advanced
483	diabetosan.mp.	0	Advanced
484	diabex.mp.	0	Advanced
485	diafat.mp.	0	Advanced
486	diaformin.mp.	0	Advanced
487	diaformina.mp.	0	Advanced
488	diametin.mp.	0	Advanced
489	diamin.mp.	0	Advanced
490	dianben.mp.	0	Advanced
491	diformin.mp.	0	Advanced
492	dimefor.mp.	0	Advanced
493	dimethylbiguanide.mp.	0	Advanced
494	dimethyldiguanide.mp.	0	Advanced
495	dmgg.mp.	0	Advanced
496	dybis.mp.	0	Advanced
497	"efb 0027".mp.	0	Advanced
498	efb0027.mp.	0	Advanced
499	eraphage.mp.	0	Advanced
500	espa–formin.mp.	0	Advanced
501	euform retard.mp.	0	Advanced
502	fluamine.mp.	0	Advanced

503	flumamine.mp.	0	Advanced
504	fornidd.mp.	0	Advanced
505	fortamet.mp.	1	Advanced
506	glafornil.mp.	0	Advanced
507	glibudon.mp.	0	Advanced
508	glifage.mp.	0	Advanced
509	gliguanid.mp.	0	Advanced
510	glucaminol.mp.	0	Advanced
511	glucofage.mp.	0	Advanced
512	glucofago.mp.	0	Advanced
513	glucoform.mp.	0	Advanced
514	glucoformin.mp.	0	Advanced
515	glucohexal.mp.	0	Advanced
516	glucoless.mp.	0	Advanced
517	glucomet.mp.	0	Advanced
518	glucomin.mp.	0	Advanced
519	glucomine.mp.	0	Advanced
520	gluconil.mp.	0	Advanced
521	glucophage.mp.	7	Advanced
522	glucostop.mp.	0	Advanced
523	glucotika.mp.	0	Advanced
524	gludepatic.mp.	0	Advanced
525	glufor.mp.	0	Advanced
526	gluformin.mp.	0	Advanced
527	glukophage.mp.	0	Advanced
528	glumeformin.mp.	0	Advanced

529	glumet.mp.	0	Advanced
530	glumetza.mp.	1	Advanced
531	glupa.mp.	0	Advanced
532	glustress.mp.	0	Advanced
533	glyciphage.mp.	0	Advanced
534	glycomet.mp.	0	Advanced
535	glycon.mp.	0	Advanced
536	glycoran.mp.	0	Advanced
537	glyformin.mp.	0	Advanced
538	glymet.mp.	0	Advanced
539	haurymellin.mp.	0	Advanced
540	hipoglucin.mp.	0	Advanced
541	i−max.mp.	0	Advanced
542	islotin.mp.	0	Advanced
543	jesacrin.mp.	0	Advanced
544	juformin.mp.	0	Advanced
545	la 6023.mp.	0	Advanced
546	la6023.mp.	0	Advanced
547	lyomet.mp.	0	Advanced
548	maformin.mp.	0	Advanced
549	meglucon.mp.	0	Advanced
550	meguan.mp.	0	Advanced
551	melbin.mp.	0	Advanced
552	melformin.mp.	0	Advanced
553	mellittin.mp.	0	Advanced
554	merckformin.mp.	0	Advanced

555	mescorit.mp.	0	Advanced
556	metaformin.mp.	0	Advanced
557	metfogamma.mp.	0	Advanced
558	metfoliquid geriasan.mp.	0	Advanced
559	metforal.mp.	0	Advanced
560	metformax.mp.	0	Advanced
561	metformin.mp.	150	Advanced
562	metformina.mp.	1	Advanced
563	metformine.mp.	2	Advanced
564	methformin.mp.	0	Advanced
565	metiguanide.mp.	0	Advanced
566	metomin.mp.	0	Advanced
567	metphormin.mp.	0	Advanced
568	miformin.mp.	0	Advanced
569	dimethylguanylguanide.mp.	0	Advanced
570	dimethylguanylguanidine.mp.	6	Advanced
571	dimethyldiguanide.mp.	0	Advanced
572	dimethyl biguanidine.mp.	0	Advanced
573	dimethylbiguanide.mp.	0	Advanced
574	dimethylbiguanidine.mp.	2	Advanced
575	dimethyldiguanide.mp.	0	Advanced
576	dimethylguanylguanidine.mp.	6	Advanced
577	neoform.mp.	1	Advanced
578	newmet.mp.	0	Advanced
579	nndg.mp.	0	Advanced
580	reglus−500.mp.	0	Advanced

581	riomet.mp.	1	Advanced
582	riomet er.mp.	0	Advanced
583	risidon.mp.	0	Advanced
584	rudimet.mp.	0	Advanced
585	siamformet.mp.	0	Advanced
586	siofor.mp.	0	Advanced
587	thiabet.mp.	0	Advanced
588	vimetrol.mp.	0	Advanced
589	walaphage.mp.	0	Advanced
590	absorcol.mp.	0	Advanced
591	adezop.mp.	0	Advanced
592	azibe.mp.	0	Advanced
593	carditimib.mp.	0	Advanced
594	cexado.mp.	0	Advanced
595	cildar.mp.	0	Advanced
596	coltowan.mp.	0	Advanced
597	corintus.mp.	0	Advanced
598	delipid.mp.	0	Advanced
599	egitim.mp.	0	Advanced
600	elanix.mp.	0	Advanced
601	erezel.mp.	0	Advanced
602	esetin.mp.	0	Advanced
603	etibax.mp.	0	Advanced
604	ezdivule.mp.	0	Advanced
605	ezedoc.mp.	0	Advanced
606	ezegelan.mp.	0	Advanced

607	ezegros.mp.	0	Advanced
608	ezehron.mp.	0	Advanced
609	ezelip.mp.	0	Advanced
610	ezemed.mp.	0	Advanced
611	ezen.mp.	0	Advanced
612	ezetad.mp.	0	Advanced
613	ezetebmib.mp.	0	Advanced
614	ezetib.mp.	0	Advanced
615	ezetiben.mp.	0	Advanced
616	ezetimib.mp.	1	Advanced
617	ezetimibe.mp.	28	Advanced
618	ezetrol.mp.	1	Advanced
619	ezetymib.mp.	0	Advanced
620	ezgal.mp.	0	Advanced
621	ezicor.mp.	0	Advanced
622	ezoleta.mp.	0	Advanced
623	hgp 1404.mp.	0	Advanced
624	hgp1404.mp.	0	Advanced
625	intestat.mp.	0	Advanced
626	kobey.mp.	0	Advanced
627	lipegis.mp.	0	Advanced
628	lipobon.mp.	0	Advanced
629	mibecol.mp.	0	Advanced
630	mibezet.mp.	0	Advanced
631	noveze.mp.	0	Advanced
632	noxetib.mp.	0	Advanced

633	olufsent.mp.	0	Advanced
634	quver.mp.	0	Advanced
635	sch 58235.mp.	1	Advanced
636	sch58235.mp.	1	Advanced
637	strami.mp.	0	Advanced
638	symezet.mp.	0	Advanced
639	tezzimi.mp.	0	Advanced
640	tritilz.mp.	0	Advanced
641	trizibe.mp.	0	Advanced
642	viemm.mp.	0	Advanced
643	vyeve.mp.	0	Advanced
644	ylerse.mp.	0	Advanced
645	zertya.mp.	0	Advanced
646	zetaxyl.mp.	0	Advanced
647	zetia.mp.	1	Advanced
648	zetiraf.mp.	0	Advanced
649	zetratak.mp.	0	Advanced
650	zetratak−s.mp.	0	Advanced
651	zient.mp.	0	Advanced
652	alirocumab.mp.	3	Advanced
653	praluent.mp.	1	Advanced
654	regn 727.mp.	1	Advanced
655	regn727.mp.	1	Advanced
656	sar 236553.mp.	1	Advanced
657	sar236553.mp.	1	Advanced
658	evolucumab.mp.	0	Advanced

659	amg 145.mp.	1	Advanced
660	amg145.mp.	1	Advanced
661	repatha.mp.	1	Advanced
662	"sal 003".mp.	0	Advanced
663	sal003.mp.	0	Advanced
664	aln 60212.mp.	0	Advanced
665	aln pcssc.mp.	0	Advanced
666	aln60212.mp.	0	Advanced
667	inclisiran.mp.	0	Advanced
668	kjx 839.mp.	0	Advanced
669	kjx839.mp.	0	Advanced
670	leqvio.mp.	0	Advanced
671	antara.mp.	1	Advanced
672	apo−feno−micro.mp.	0	Advanced
673	apteor.mp.	0	Advanced
674	aterolis.mp.	0	Advanced
675	biofibrat.mp.	0	Advanced
676	bisterol sr.mp.	0	Advanced
677	catalip.mp.	2	Advanced
678	climage.mp.	0	Advanced
679	controlip.mp.	4	Advanced
680	durafenat.mp.	4	Advanced
681	elipsia.mp.	0	Advanced
682	evothyl.mp.	0	Advanced
683	febira.mp.	0	Advanced
684	fegenor.mp.	2	Advanced

685	felosma.mp.	0	Advanced
686	fenardin.mp.	0	Advanced
687	fenobrat.mp.	0	Advanced
688	fenobrate.mp.	2	Advanced
689	fenochol.mp.	0	Advanced
690	fenofanton.mp.	3	Advanced
691	fenofibrate.mp.	28	Advanced
692	fenofix.mp.	0	Advanced
693	fenogal.mp.	2	Advanced
694	fenoglide.mp.	1	Advanced
695	fenolip.mp.	2	Advanced
696	fenomax.mp.	0	Advanced
697	fenosup lidose.mp.	0	Advanced
698	fenoswiss.mp.	0	Advanced
699	fenox.mp.	0	Advanced
700	fibrafen.mp.	0	Advanced
701	fulcro.mp.	2	Advanced
702	fulcrosupra.mp.	0	Advanced
703	grofibrat.mp.	0	Advanced
704	"grs 001".mp.	0	Advanced
705	grs001.mp.	0	Advanced
706	hyperchol.mp.	0	Advanced
707	katalip.mp.	0	Advanced
708	lexemin.mp.	2	Advanced
709	lifenoz−u.mp.	0	Advanced
710	lipanthyl.mp.	4	Advanced

711	lipanthyl.mp.	4	Advanced
712	lipanthylnano.mp.	0	Advanced
713	lipantil.mp.	4	Advanced
714	lipantyl.mp.	0	Advanced
715	liparison.mp.	4	Advanced
716	lipcor.mp.	2	Advanced
717	liperial.mp.	0	Advanced
718	lipidax.mp.	2	Advanced
719	lipidil.mp.	3	Advanced
720	lipilfen.mp.	0	Advanced
721	lipilo.mp.	0	Advanced
722	lipirex.mp.	2	Advanced
723	lipoclar.mp.	2	Advanced
724	lipofen.mp.	3	Advanced
725	lipofene.mp.	2	Advanced
726	lipofib.mp.	0	Advanced
727	lipolin.mp.	0	Advanced
728	liposit.mp.	2	Advanced
729	lipsin.mp.	2	Advanced
730	livesan ge.mp.	0	Advanced
731	lofibra.mp.	3	Advanced
732	nopid 200.mp.	0	Advanced
733	normalip.mp.	4	Advanced
734	nubrex.mp.	0	Advanced
735	procetofen.mp.	5	Advanced
736	procetofenate.mp.	0	Advanced

737	procetofene.mp.	3	Advanced
738	procetofen.mp.	5	Advanced
739	qualipantyl.mp.	0	Advanced
740	rapidil.mp.	1	Advanced
741	redose 200.mp.	0	Advanced
742	rorit.mp.	0	Advanced
743	rp 1824.mp.	0	Advanced
744	rp1824.mp.	0	Advanced
745	secalip.mp.	4	Advanced
746	sigurtil.mp.	0	Advanced
747	supralip.mp.	4	Advanced
748	suprelip.mp.	0	Advanced
749	trichol.mp.	0	Advanced
750	tricor.mp.	4	Advanced
751	triglide.mp.	1	Advanced
752	trolip.mp.	0	Advanced
753	xafenor.mp.	0	Advanced
754	zerlubron.mp.	0	Advanced
755	ziglip.mp.	0	Advanced
756	zigotrig.mp.	0	Advanced
757	zumafib.mp.	0	Advanced
758	ausgem.mp.	2	Advanced
759	bolutol.mp.	2	Advanced
760	brozil.mp.	0	Advanced
761	chlorestrol.mp.	0	Advanced
762	cholespid.mp.	0	Advanced

763	ci 719.mp.	19	Advanced
764	ci719.mp.	3	Advanced
765	clearol.mp.	0	Advanced
766	decrelip.mp.	2	Advanced
767	detrichol.mp.	0	Advanced
768	elmogan.mp.	1	Advanced
769	fetinor.mp.	0	Advanced
770	fibralip.mp.	0	Advanced
771	fibrocit.mp.	1	Advanced
772	gedum.mp.	0	Advanced
773	gemfi.mp.	2	Advanced
774	gemfibril.mp.	1	Advanced
775	gemfibromax.mp.	1	Advanced
776	gemfibrozil.mp.	17	Advanced
777	gemfibrozil.mp.	17	Advanced
778	gemfibrozilo.mp.	0	Advanced
779	gemhexal.mp.	2	Advanced
780	gemizol.mp.	1	Advanced
781	gemlipid.mp.	1	Advanced
782	gemnpid.mp.	1	Advanced
783	gemzil.mp.	0	Advanced
784	genfibrozil.mp.	0	Advanced
785	gevilon.mp.	0	Advanced
786	gevilon uno.mp.	0	Advanced
787	gozid.mp.	1	Advanced
788	grifogemzilo.mp.	0	Advanced

789	hidil.mp.	1	Advanced
790	hipolixan.mp.	0	Advanced
791	ipolipid.mp.	1	Advanced
792	jezil.mp.	2	Advanced
793	lanaterom.mp.	0	Advanced
794	lifibron.mp.	0	Advanced
795	lipazil.mp.	2	Advanced
796	lipidys.mp.	1	Advanced
797	lipigem.mp.	0	Advanced
798	lipira.mp.	0	Advanced
799	lipison.mp.	1	Advanced
800	lipistorol.mp.	0	Advanced
801	lipizyl.mp.	0	Advanced
802	lipofor.mp.	1	Advanced
803	lipolo.mp.	0	Advanced
804	lipostorol.mp.	0	Advanced
805	lipox gemfi.mp.	1	Advanced
806	lipozid.mp.	1	Advanced
807	lipozil.mp.	1	Advanced
808	lipur.mp.	2	Advanced
809	litarek.mp.	2	Advanced
810	lopid.mp.	3	Advanced
811	lowin.mp.	1	Advanced
812	low−lip.mp.	0	Advanced
813	manobrozil.mp.	0	Advanced
814	mariston.mp.	1	Advanced

815	mersikol.mp.	0	Advanced
816	normolip.mp.	0	Advanced
817	panazil.mp.	0	Advanced
818	pilder.mp.	2	Advanced
819	polyxit.mp.	1	Advanced
820	progemzal.mp.	0	Advanced
821	q8x02027x3.mp.	0	Advanced
822	recozil.mp.	1	Advanced
823	reducel.mp.	0	Advanced
824	regulip.mp.	0	Advanced
825	synbrozil.mp.	1	Advanced
826	trialmin.mp.	2	Advanced
827	triglizil.mp.	0	Advanced
828	uragem.mp.	0	Advanced
829	zilop.mp.	0	Advanced
830	or/1-829	1709	Advanced
831	aneurysm?.ti,ab.	49	Advanced
832	aortic rupture*.ti,ab.	2	Advanced
833	loeys-dietz.ti,ab.	0	Advanced
834	aortic dissection?.ti,ab.	9	Advanced
835	aorta dissection?.ti,ab.	0	Advanced
836	aortic dilatation?.ti,ab.	1	Advanced
837	aortic ectasia?.ti,ab.	0	Advanced
838	((aorta or aortic) and (surgery or surgical)).ti,ab.	49	Advanced
839	(Bentall adj2 (repair or replacement or surgery)).ti,ab.	0	Advanced
840	(David adj2 (repair or surgery)).ti,ab.	0	Advanced

841	(aortic root adj2 (repair or replacement)).ti,ab.	0	Advanced
842	(Yacoub adj2 (repair or surgery)).ti,ab.	0	Advanced
843	(hemiyacoub adj2 (repair or surgery)).ti,ab.	0	Advanced
844	frozen elephant trunk surgery.ti,ab.	0	Advanced
845	(hemiarch adj2 (repair or replacement)).ti,ab.	0	Advanced
846	(marfan* adj2 syndrome*).ti,ab.	1	Advanced
847	aortic h?ematoma.ti,ab.	0	Advanced
848	aortic ulcer*.ti,ab.	0	Advanced
849	tevar.ti,ab.	8	Advanced
850	evar.ti,ab.	11	Advanced
851	aortic endoprosthesis.ti,ab.	0	Advanced
852	(aortic adj2 replacement).ti,ab.	6	Advanced
853	aortic growth.ti,ab.	0	Advanced
854	or/831-853	75	Advanced
855	830 and 854	15	Advanced
