# Supplementary material for: Circulating Lipids, Statins, and Metformin in Thoracic and Abdominal Aortic Aneurysm: A Systematic Review and Meta-Analysis
Source: Life (Basel). 2026 Oct 21;16(10):1765. doi: 10.3390/life16101765 (PMC13706600; doi:10.3390/life16101765)

SUPPLEMENTARY MATERIALS

Table S1. GRADE assessment of the certainty of evidence and outcome definitions by study. (A) Certainty of evidence for circulating lipid fractions and for statin and metformin use in relation to aneurysm-related outcomes.

№ of studies	Certainty assessment					Effect	Certainty	Importance
	Risk of bias	Inconsistency	Indirectness	Imprecision	Other considerations			
**Cholesterol and outcomes**								
0								CRITICAL
**Cholesterol and aneurysm growth**								
4	serious	serious	not serious	serious	none	No consistent association	⨁◯◯◯ Very low	IMPORTANT
**Statins and aortic events**								
5	serious	not serious	not serious	serious	none	Significant reduction in 4 articles<br>Non-significant reduction in 1 article	⨁⨁◯◯ Low	CRITICAL
**Statins and mortality**								
4	serious	not serious	not serious	not serious	none	Pooled HR 0.80 (95% CI 0.77–0.83); I^2 = 0.0%<br>1 study not pooled: OR 0.29, p = 0.049	⨁⨁◯◯ Low	CRITICAL
**Statins and aneurysm growth**								
12	serious	serious	not serious	not serious	none	Pooled MD −0.78 mm/year (95% CI −1.13 to −0.43); I^2 = 92.4%; 95% prediction interval −2.02 to 0.46	⨁⨁◯◯ Low	IMPORTANT

№ of studies	Certainty assessment					Effect	Certainty	Importance
	Risk of bias	Inconsistency	Indirectness	Imprecision	Other considerations			
**Metformin and outcomes**								
2	serious	not serious	not serious	serious	none	Significant reduction in mortality and aortic events	⊕◯◯◯ Very low	CRITICAL
**Metformin and aneurysm growth**								
6 (8 cohorts) + 1 RCT	serious	not serious	not serious	not serious	none	Observational cohorts: MD −0.34 mm/year (95% CI −0.48 to −0.20); I^2 = 46.3%. Including the randomized trial: MD −0.32 mm/year (95% CI −0.46 to −0.19)	⊕⊕◯◯ Low	IMPORTANT

(B) Outcome definitions and effect measures for the studies reporting clinical outcomes. Reference numbers refer to the main article.

Study	Aneurysm population	Exposure contrast	Outcome definition	Effect measure
**Statins**				
Angeloni et al. (2015) [27]	Ascending TAA	Statin vs. no statin	Composite of death, aortic dissection or rupture, or surgical repair at 3 years	HR
Jovin et al. (2012) [37]	TAA	Statin vs. no statin	All-cause death (crude HR, median 3.6 years); composite of death, rupture, dissection or repair	HR
Stein et al. (2013) [38]	TAA	Statin vs. no statin	Death, dissection or rupture; need for surgical repair	OR
Tazaki et al. (2014) [39]	Aortic aneurysm or dissection in patients undergoing coronary revascularization (31% with prior aneurysm repair)	Statin at discharge vs. no statin	Composite of all-cause death, myocardial infarction and stroke	Adjusted HR
Sukhija et al. (2006) [40]	Non-operated infrarenal AAA	Statin vs. no statin	All-cause mortality	OR
Mosorin et al. (2008) [41]	Small infrarenal AAA	Statin vs. no statin	Freedom from aneurysm repair or rupture	Unadjusted comparison only
Gokani et al. (2015) [42]	Large infrarenal AAA	Statin vs. no statin	Rupture at presentation (ruptured vs. elective presentation)	OR
Bahia et al. (2016) [43]	AAA	Statin vs. no statin	All-cause death ascertained from general-practice records, modeled with time-dependent covariates	HR
Skovbo et al. (2025) [34]	Infrarenal AAA 30–55 mm	Statin dose (defined daily doses)	Aneurysm repair; composite of repair, rupture and all-cause death	HR per doubling of dose

Study	Aneurysm population	Exposure contrast	Outcome definition	Effect measure
**Metformin**				
Golledge et al. (2017) [44]	Infrarenal AAA	Metformin vs. no metformin	Composite of AAA repair or rupture-related mortality	HR
Sutton et al. (2020) [36]	Infrarenal AAA	Metformin-exposed diabetics vs. non-exposed diabetics and non-diabetics	AAA surgery; all-cause death; death after surgery (multistate model)	HR

AAA, abdominal aortic aneurysm; HR, hazard ratio; OR, odds ratio; RCT, randomized controlled trial; TAA, thoracic aortic aneurysm.

Figure S1. Risk of bias of the included observational studies assessed with the ROBINS-I tool, by outcome. Green indicates low, yellow moderate, orange serious and red critical risk of bias. The domain of bias due to deviations from intended interventions was

considered not applicable to exposures recorded in routine care and is therefore not shown.

	Confounding	Participant selection	Classification of interventions	Missing data	Outcome measurement	Selection of the reported result	Overall
**Lipid fractions and aneurysm growth**							
Deeg 2016	Serious	Low	Low	Moderate	Low	Low	Serious
Ferguson 2010	Serious	Low	Low	Serious	Low	Low	Serious
Lindholt 2001	Serious	Low	Low	Serious	Low	Low	Serious
Schlösser 2008	Serious	Low	Low	Serious	Low	Low	Serious
**Statins and aortic events**							
Angeloni 2015	Moderate	Moderate	Low	Low	Low	Low	Moderate
Gokani 2015	Moderate	Serious	Low	Moderate	Moderate	Low	Serious
Jovin 2012	Moderate	Low	Low	Moderate	Moderate	Low	Moderate
Stein 2013	Moderate	Low	Low	Moderate	Moderate	Low	Moderate
**Statins and all-cause mortality**							
Bahia 2016	Moderate	Low	Low	Moderate	Low	Low	Moderate
Jovin 2012	Moderate	Low	Low	Moderate	Low	Low	Moderate
Sukhija 2006	Serious	Serious	Low	Low	Low	Low	Serious
Tazaki 2014	Moderate	Moderate	Low	Moderate	Low	Low	Moderate
**Statins and aneurysm growth**							
Angeloni 2015	Moderate	Moderate	Low	Low	Low	Low	Moderate
Karrowni 2011	Moderate	Low	Low	Moderate	Low	Low	Moderate
Lederle 2015	Moderate	Low	Low	Low	Low	Low	Moderate
Mosorin 2008	Serious	Low	Low	Moderate	Low	Low	Serious
Periard 2012	Moderate	Low	Low	Moderate	Low	Low	Moderate
Schlösser 2008	Serious	Low	Low	Serious	Low	Low	Serious
Jovin 2012	Serious	Low	Low	Moderate	Low	Low	Serious
Schouten 2006	Moderate	Low	Low	Moderate	Low	Low	Moderate
Sukhija 2006	Serious	Serious	Low	Low	Low	Low	Serious
Karlsson 2009	Serious	Moderate	Low	Low	Low	Low	Serious
Sweeting 2010	Moderate	Low	Low	Low	Low	Low	Moderate
Stein 2013	Serious	Low	Low	Moderate	Low	Low	Serious
**Metformin and clinical outcomes**							
Sutton 2020	Moderate	Moderate	Low	Moderate	Low	Low	Moderate
Golledge 2017	Moderate	Low	Low	Moderate	Low	Low	Moderate
**Metformin and aneurysm growth**							
Gellatly 2024	Moderate	Low	Low	Moderate	Low	Low	Moderate
Golledge 2017 (cohort 1)	Moderate	Low	Low	Moderate	Low	Low	Moderate
Golledge 2017 (cohort 2)	Moderate	Low	Low	Moderate	Low	Low	Moderate
Golledge 2017 (cohort 3)	Moderate	Low	Low	Moderate	Low	Low	Moderate
Itoga 2019	Moderate	Low	Low	Moderate	Low	Low	Moderate
Unosson 2021	Moderate	Low	Low	Moderate	Low	Low	Moderate
Bobadilla-Rosado 2024	Serious	Low	Low	Moderate	Low	Low	Serious
Thompson 2010	Moderate	Low	Low	Moderate	Low	Low	Moderate

Low   Moderate   Serious   Critical

Figure S2. Funnel plot for the association between statin use and aneurysm growth (12 studies). Egger's test p = 0.13. θIV, inverse-variance pooled estimate.

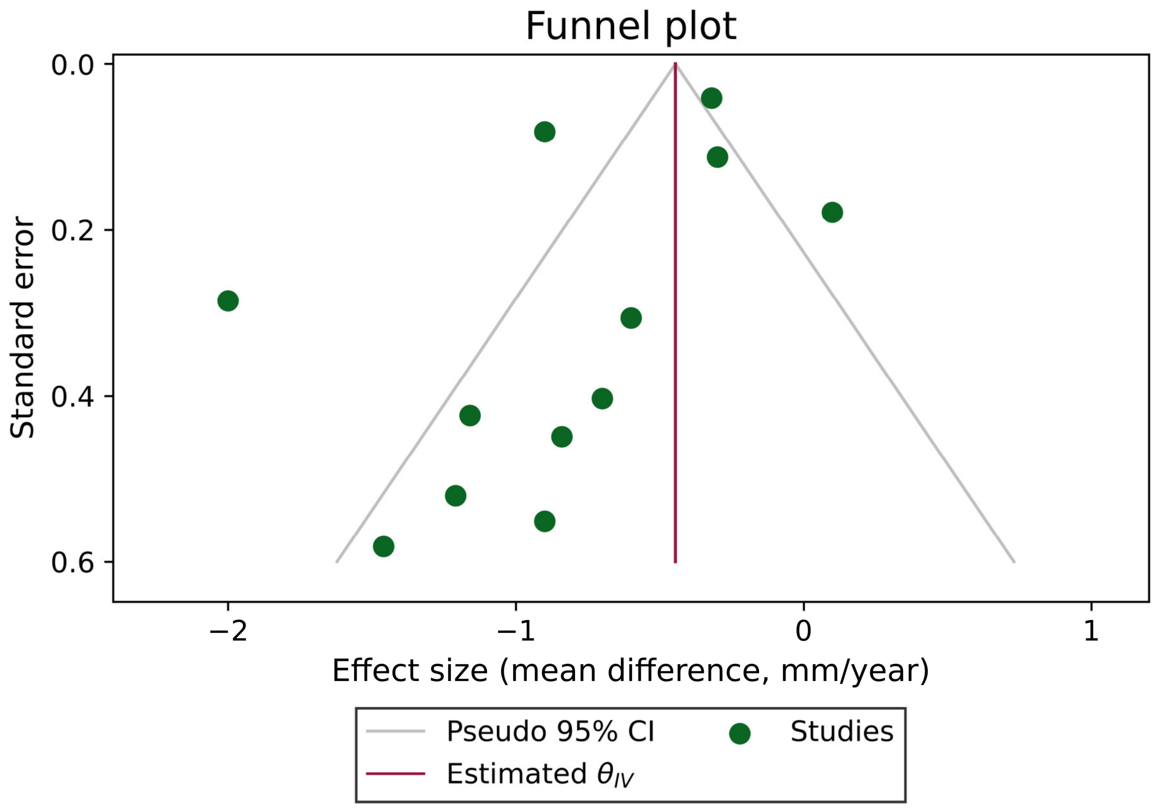

Figure S3. Funnel plot for the association between metformin use and aneurysm growth (8 observational cohorts from 6 studies). Egger's test p = 0.12. θIV, inverse-variance pooled estimate.

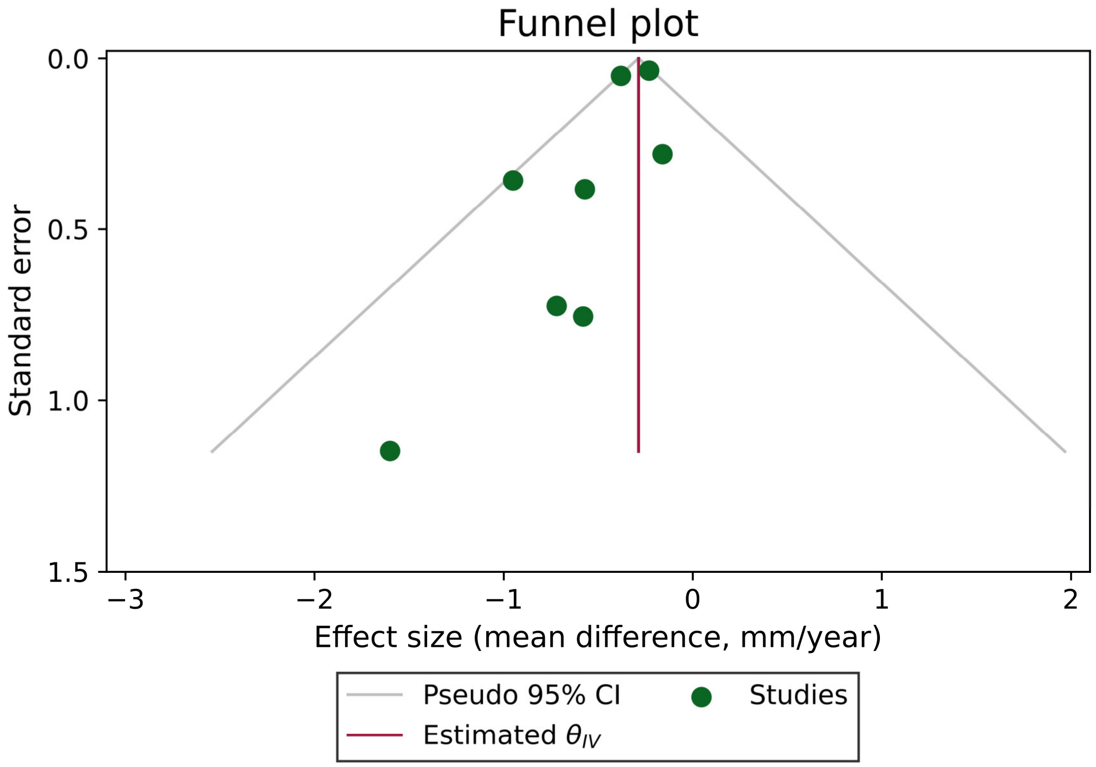

Supplement: Supplementary file 1 [file life-16-01765-s001.zip › Table S1 and Figures S1-S3.pdf]
